# Effect of Teaching Bayesian Methods Using Learning by Concept vs Learning by Example on Medical Students’ Ability to Estimate Probability of a Diagnosis: A Randomized Clinical Trial

**DOI:** 10.1001/jamanetworkopen.2019.18023

**Published:** 2019-12-20

**Authors:** John E. Brush, Mark Lee, Jonathan Sherbino, Judith C. Taylor-Fishwick, Geoffrey Norman

**Affiliations:** 1 Cardiology Division, Department of Internal Medicine, Eastern Virginia Medical School, Sentara Healthcare, Norfolk; 2 McMaster Education Research, Innovation and Theory Program, McMaster University, Hamilton, Ontario, Canada

## Abstract

**Importance:**

Clinicians use probability estimates to make a diagnosis. Teaching students to make more accurate probability estimates could improve the diagnostic process and, ultimately, the quality of medical care.

**Objective:**

To test whether novice clinicians can be taught to make more accurate bayesian revisions of diagnostic probabilities using teaching methods that apply either explicit conceptual instruction or repeated examples.

**Design, Setting, and Participants:**

A randomized clinical trial of 2 methods for teaching bayesian updating and diagnostic reasoning was performed. A web-based platform was used for consent, randomization, intervention, and testing of the effect of the intervention. Participants included 61 medical students at McMaster University and Eastern Virginia Medical School recruited from May 1 to September 30, 2018.

**Interventions:**

Students were randomized to (1) receive explicit conceptual instruction regarding diagnostic testing and bayesian revision (concept group), (2) exposure to repeated examples of cases with feedback regarding posttest probability (experience group), or (3) a control condition with no conceptual instruction or repeated examples.

**Main Outcomes and Measures:**

Students in all 3 groups were tested on their ability to update the probability of a diagnosis based on either negative or positive test results. Their probability revisions were compared with posttest probability revisions that were calculated using the Bayes rule and known test sensitivity and specificity.

**Results:**

Of the 61 participants, 22 were assigned to the concept group, 20 to the experience group, and 19 to the control group. Approximate age was 25 years. Two participants were first-year; 37, second-year; 12, third-year; and 10, fourth-year students. Mean (SE) probability estimates of students in the concept group were statistically significantly closer to calculated bayesian probability than the other 2 groups (concept, 0.4%; [0.7%]; experience, 3.5% [0.7%]; control, 4.3% [0.7%]; *P* < .001). Although statistically significant, the differences between groups were relatively modest, and students in all groups performed better than expected, based on prior reports in the literature.

**Conclusions and Relevance:**

The study showed a modest advantage for students who received theoretical instruction on bayesian concepts. All participants’ probability estimates were, on average, close to the bayesian calculation. These findings have implications for how to teach diagnostic reasoning to novice clinicians.

**Trial Registration:**

ClinicalTrials.gov identifier: NCT04130607

## Key Points

**Question:**

Can novice clinicians be taught to make more accurate bayesian revisions of diagnostic probabilities using teaching methods involving either explicit conceptual instruction or repeated examples?

**Findings:**

In this randomized clinical trial of 61 medical students, explicit conceptual instruction on bayesian reasoning and concepts significantly improved the accuracy of posttest probability estimation for novice clinicians, whereas exposure to repeated examples did not. The ability to estimate diagnostic probability was better than expected for all 3 experimental conditions (explicit instruction, repeated examples, and control).

**Meaning:**

Explicit theoretical instruction significantly improved bayesian revisions of diagnostic probabilities, which has implications for teaching diagnostic reasoning to novice clinicians.

## Teaser

This randomized clinical trial evaluates whether medical students can be taught to make more accurate bayesian revisions of diagnostic probabilities using learning by concept or learning by example.

## Introduction

To make a diagnosis, a clinician initially considers multiple diagnostic possibilities and then frequently performs testing to determine the most likely diagnosis.^1,2,3^ Ideally, the probability of a diagnosis is correctly revised based on the test result, the pretest probability, and the test characteristics (sensitivity and specificity). Mathematically, the optimal probability revision is determined through use of the Bayes rule.^4,5,6,7,8^

However, practicing clinicians rarely explicitly calculate probabilities; instead, if asked, they may provide a subjective probability estimate based on their experience. Several studies have reported that physicians’ subjective probability estimates can be highly inaccurate.^9,10^ Generally, probability estimates are usually too conservative (ie, not sufficiently changed by updating information compared with a bayesian calculation) or simply error prone.^11,12,13,14^

The aim of this study was to determine whether novice clinicians (ie, medical students) could be taught to accurately estimate diagnostic probabilities. We sought to compare explicit teaching of the concepts of probability revision based on bayesian analysis with a second intervention where students acquired relevant experience by exposure to repeated examples and feedback on their probability revision, and with a third control condition, to determine the effect of 2 kinds of instruction on the students’ ability to accurately estimate the posttest probability of a diagnosis.

## Methods

### Study Population

In this study, we evaluated the performance of medical students from McMaster University, Hamilton, Ontario, Canada, and Eastern Virginia Medical School, Norfolk. Students in their late second, third, and fourth year of medical school were recruited by email from May 1 to September 30, 2018. Participants gave informed consent by computer at the time of enrollment. Participants were given a small stipend to compensate for their time spent participating in the study. The study was approved by the Hamilton Integrated Research Ethics Board and the Eastern Virginia Medical School Institutional Review Board. The trial protocol is available in Supplement 1. This study followed the Consolidated Standards of Reporting Trials (CONSORT) reporting guideline.^15^

### Intervention

The study consisted of a learning phase containing 1 of 2 experimental conditions, a control condition, and a delayed assessment phase. During the learning phase, students were randomly allocated to 1 of 3 conditions: concept, experience, or control.

In the concept condition, students were shown an 18-minute instructional video in which they were introduced to the anchoring and adjusting heuristic as an intuitive equivalent to bayesian reasoning. They were taught to use this simplifying heuristic to help them use pretest probability (anchoring) and bayesian updating (adjusting) to estimate posttest probability. The video gave instruction on the concepts of base rates, sensitivity, specificity, and likelihood ratios and how these concepts could be used to help them calibrate their subjective probability estimates (Video). Students were also shown 1 example from each of the 3 diagnostic categories used in the experience condition.

**Video.** Concept Teaching Video Eighteen-minute video shown to participants randomized to the concept trial arm teaching the anchoring and adjusting heuristic as an intuitive equivalent to Bayesian reasoning.

In the experience condition, students worked through 9 written cases for each of 3 diagnostic categories and a corresponding diagnostic test: pulmonary embolus and d-dimer testing, congestive heart failure and chest radiograph, and acute coronary syndrome and troponin testing. For each case, participants were provided a history of the presenting illness, medical history, physical examination, and the results of the diagnostic test. In each case, participants received feedback on the most likely diagnosis in an effort to teach through repeated examples.

In the control condition, students were given an opportunity to briefly read about the same 3 diagnostic categories. They were given no examples or explicit instruction focused on bayesian logic.

During the assessment phase, students were given written cases containing clinical findings and asked to provide a pretest probability of the diagnosis. Then, they received the diagnostic test result (either positive or negative) and were asked to provide a posttest probability estimate of the diagnosis. To assess transfer of learning, students were tested using a set of cases from a new diagnosis (pneumonia), as well as sets of cases from the same 3 diagnoses that were used in the learning phase. All students were tested on a total of 20 cases: 4 cases from each of the diagnostic categories used in the learning condition for a total of 12 cases, 4 new cases of a new diagnostic category, and 4 filler cases, which were not analyzed. The role of the filler cases was to avoid the expectation that every condition would have been previously encountered. Test cases were designed to have a low, medium, and high pretest probability of the disease category and to include both positive and negative diagnostic test results. Because participants were not expected to know the sensitivity, specificity, and likelihood ratios of particular tests, these values were provided with the questions administered in the concept condition.

The study was performed via a web-based platform that provided the learning phase content, recorded the students’ responses, and timed students’ activity during the study (using LimeSurvey, an ethics-compliant survey service provided by McMaster University). When the learning phase was complete, students were asked to sign off and were told that the assessment phase would be available upon sign-in after a 24-hour lockout period. Students were expected to complete the entire study within a 72-hour time frame. All of the content provided to study participants and the test questions are available in the eAppendix in Supplement 2.

### Statistical Analysis

First, initial pretest and posttest probability estimates were analyzed using descriptive statistics to assess overall performance in revising probability estimates. Next, a score for subjective change from pretest probability to posttest was computed, and a bayesian change score was calculated using the student’s pretest probability estimate and the calculated posttest probability based on published estimates of sensitivity and specificity.^16^ The differences between subjective and bayesian change scores were then analyzed using a mixed-model analysis of variance with the experimental group as a between-subject factor and the case as a within-subject factor. A separate analysis was performed on the 12 cases with the same 3 diagnostic categories as in the learning phase (learning cases) and the 4 cases with a new diagnostic category (new cases) to determine the extent to which the learned skills were generalizable to a diagnosis and a diagnostic test result that had not been previously encountered. Separate analyses were conducted for revisions based on positive or negative diagnostice test results. Filler cases were not analyzed.

For the primary analysis, revisions resulting from positive and negative test results were combined by reversing the sign for the revisions from the negative test results. A mixed-model analysis of variance was again used, with 1 between-participant factor (intervention group, 3 levels) and 1 within-participant factor (case). Analysis was performed on all 16 cases, on the 12 cases with the same diagnosis as the learning phase, and on the 4 cases with a new diagnostic category. Results are reported as mean (SE). Timing of student responses by experimental group was also analyzed. Analyses were performed using SPSS, version 25 (IBM Corp). Two-tailed, unpaired *P* < .05 was considered statistically significant.

## Results

**Figure 1.**
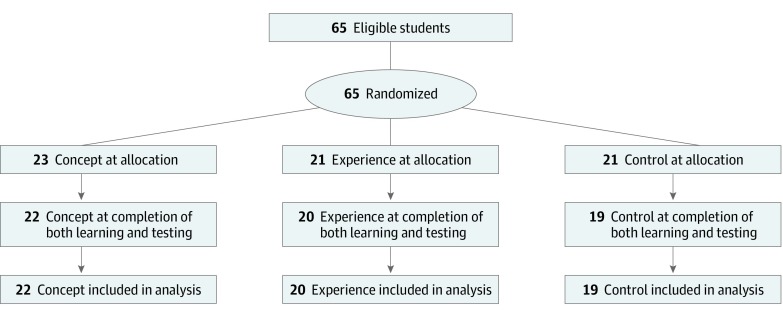
Participant Flow Diagram The process of randomization, study completion, and analysis for students allocated to the 3 conditions.

A total of 65 students were recruited: 43 at McMaster University and 22 at Eastern Virginia Medical School. Twenty-three participants were randomly allocated to the concept condition, 21 to the experience condition, and 21 to the control condition. Sixty-one students completed both the learning phase and the assessment phase and were included in the analysis. Participant characteristics are listed in Table 1, and a participant flow diagram^15^ is shown in Figure 1.

**Table 1.** Characteristics of Participants

Condition	Participants, No.	Approximate Age, y	No. of Participants by Year in School			
			First	Second	Third	Fourth
Concept	22	25	2	12	5	3
Experience	20	26	0	13	2	5
Control	19	25	0	12	5	2
Total	61	25	2	37	12	10

**Figure 2.**
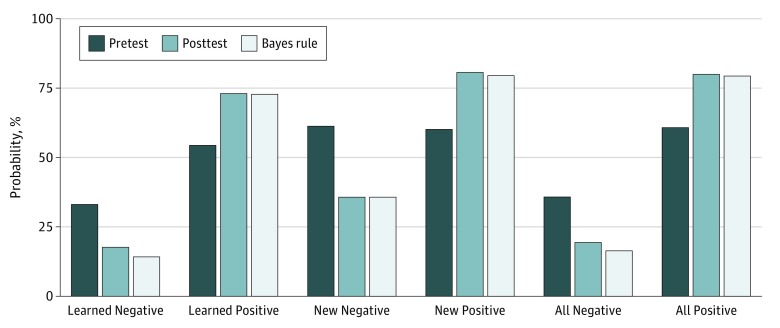
Participants’ Estimates of Pretest Probability, Posttest Probability, and the Calculated Probability Based on Bayes Rule Cases with negative test results and positive test results by whether the cases were learned cases or new cases, and all cases.

An initial descriptive analysis examined the mean estimates of pretest probability and subjective posttest probability, as well as the bayesian calculation of posttest probability across all groups. The intention was to verify that subjective revisions were directionally correct and conservative, as expected from previous research.^9,10,11^ As shown in Figure 2, study participants’ estimates of posttest probability were close to the calculated value using the Bayes rule. This close correspondence was seen in both learned cases and in cases involving new, unfamiliar diagnoses and was noted for both positive and negative revisions. There was some evidence of conservatism in the subjective revisions for negative cases, but the effect was small (approximately 2%).

**Figure 3.**
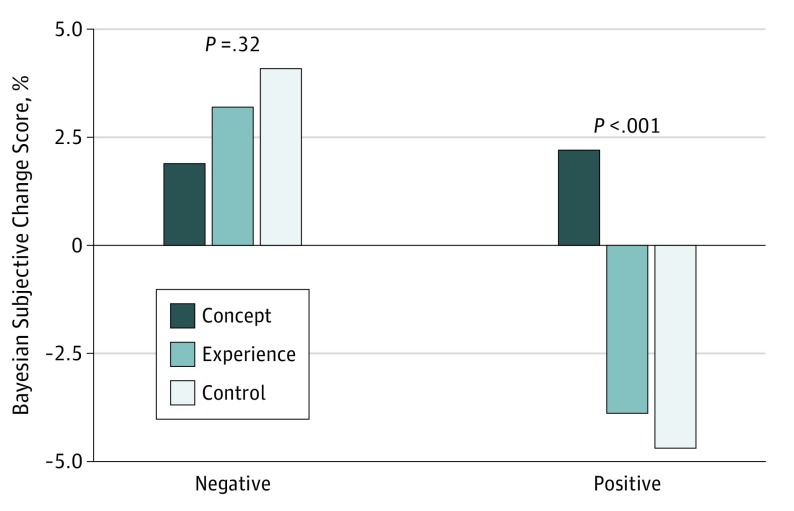
Difference Between the Calculated Bayesian Change Score and the Subjective Change Score for Each Intervention Group by Positive or Negative Test Results

To contrast subjective and calculated bayesian revisions and examine the effect of the interventions, we evaluated the difference between the subjective change score and the bayesian change score for each intervention group and for positive or negative test results, as shown in Figure 3. For a positive test result, a negative difference implies that the subjective change was smaller than the bayesian change (ie, conservatism). Conversely, for a negative test result, because all changes are in a negative direction, conservatism is evidenced by a positive difference. As Figure 3 shows, for negative tests, all experimental conditions resulted in a conservative revision, although all differences were less than 4%. For a positive test, the concept condition was slightly liberal; the others were slightly conservative. Again, all discrepancies from bayesian revision were small—less than 5%.

For both positive and negative test results, the concept condition tended to be more accurate, amounting to an average discrepancy between the posttest subjective and bayesian estimates of about 1.5%, which was less than half that of the other 2 groups. For cases with negative test results, the difference between groups was not significant (*F* = 1.15, *P* = .32). For positive diagnostic test results, the estimates from the experience and control groups were conservative, but the concept revisions were slightly liberal, and the difference between groups was significant (*F* = 12.48, *P* < .001). Case-by-case examination showed that this difference varied by case. However, only 5 of the 48 (16 cases × 3 conditions) mean revisions examined showed a difference between subjective posttest and bayesian estimates greater than 10%, and none exceeded a difference of 15%.

For the primary analysis, positive and negative test revisions were combined by reversing the sign for the revisions of the negative test results to compare the 3 experimental conditions. The concept condition was statistically significantly more accurate, with a mean (SE) discrepancy between the posttest subjective and bayesian estimates of only 0.4% (0.7%), compared with 3.5% (0.7%) for the experience group and 4.3% (0.7%) for the control group (*F* = 9.07, *P* < .001).

To determine whether the learning generalized to different diagnoses (transfer), a comparison of the diagnostic accuracy of cases from a new diagnostic category (pneumonia) vs cases from the learning condition was analyzed as reported in Table 2. Test cases from the learning diagnoses showed a small, nonsignificant advantage for the concept condition (1.2% difference vs 3.1% for experience and 3.1% for control [*F* = 1.80, *P* = .17]). Cases from the new diagnosis showed a statistically significant advantage for the concept condition (−2.0% liberal revision vs 4.6% for experience and 7.9% for control [*F* = 8.74, *P* < .001]).

**Table 2.** Difference Between Calculated Bayesian Change Score and Subjective Change Score for Each Intervention Group

Intervention	Mean (SE), %	
	Learning	New
Concept	1.2 (0.8)	−2.0 (1.6)
Experience	3.1 (0.9)	4.6 (1.7)
Control	3.1 (0.9)	7.9 (1.8)
*F* value	1.80	8.74
*P* value	.17	<.001

The time to complete each test case was analyzed by condition. The participants in the concept condition spent a mean (SE) of 24.6 (1.9) seconds responding to the test questions, compared with 10.8 (2.0) seconds for the experience condition and 11.7 (2.0) seconds for the control condition (*F* = 15.43, *P* < .001).

## Discussion

Our study showed that explicit conceptual instruction on bayesian reasoning significantly improved posttest probability estimation in novice clinicians. Although bayesian reasoning has been widely promoted in the literature as a diagnostic strategy,^4,5,6,7,8^ there has been scant evidence that teaching bayesian reasoning actually improves diagnostic accuracy. Providing learners with relatively brief instruction on these abstract concepts appeared to significantly improve their diagnostic performance in comparison with simply providing a number of relevant examples or no relevant instruction.

The advantage of the concept condition was accompanied by a longer testing time, suggesting that students were attempting to frame the question within the conceptual framework that was presented in the Video. The extra time for this condition, however, did not appear to be sufficient to allow actual calculation of posttest probability.

When the cases were analyzed by learning cases vs new cases, the advantage of the concept condition was not statistically significant in the learning cases but was statistically significant with the cases from a new diagnostic category, suggesting some transfer of concepts to the new cases. However, the differences are small and, despite statistical significance, should be viewed as tentative.

The advantage that emerged from the concept intervention is consistent with prior research suggesting that providing students with a conceptual framework can improve their ability to solve clinical problems.^17^ Enabling an explicit connection between basic concepts and clinical examples has been proposed as a mechanism for improving medical education in general.^18,19^

The results for the experience condition were not statistically significantly different from those of the control condition. The learning phase in the experience condition consisted of exposure to 9 cases per diagnostic category (a total of 27 cases), and it is possible that far more examples are necessary before intuitive judgment from repeated exposure is sufficient to affect the accuracy of probability estimation.

An unexpected finding of our study was how well the participants in all conditions estimated posttest probability. This finding is inconsistent with previous literature on errors in human judgment, which has concluded that humans (including clinicians) are suboptimal in bayesian reasoning, inadequately taking into account the base rates of outcomes and conservatively revising probabilities in light of new information.^9,10,11,12,13,14^ It is possible that the overall study design alerted the participants to the goals and task assumptions of the exercise.^20^ It is also possible that prior educational exposure to clinical cases or prior formal teaching in the medical school curriculum affected all participants. However, exactly how these factors may have affected the accuracy of probability revisions in this study is unclear.

The previously reported discrepancy between human probability estimation and optimal bayesian probability estimation has become traditionally accepted in the psychology and medical literature as cognitive biases, including base-rate neglect, anchoring bias, confirmation bias, and representativeness, all of which suggest suboptimal revision and have been purported to be a primary cause of diagnostic error.^13,14^ However, on closer scrutiny, much of this evidence was derived from situations that were not representative of the typical diagnostic setting. One highly cited study^9^ used a screening situation in which the base rate was very low and any positive test result, even one with excellent operating characteristics, would have most likely been a false-positive result. Participants in that study also appeared to exhibit semantic confusion by confusing the posttest probability with the conditional probability that was presented in the problem-solving exercise.^20^

Despite long-standing calls for using bayesian reasoning in clinical medicine, the concepts are not generally taught in a formal fashion in most medical schools. How then do physicians learn to incorporate new information into their diagnostic probabilities? Teaching reasoning as a formal discipline has been promoted for centuries, but according to Nisbett et al,^21^ teaching abstract rules of reasoning fell into disfavor in the 20th century. The prevailing notion was that people do not use abstract inferential rules, but rather use domain-specific empirical rules that deal with specific events, and such rules are learned by experience, not instruction. Further work has been more optimistic and has indicated that statistical heuristics and pragmatic inferential rules can be effectively taught, even with brief formal training.^21,22,23,24^ The research reported by Elstein et al^1^ also found that heuristic training improved medical students’ ability to adapt their thinking to the demands of medical problems. According to Nisbett, “The key is learning how to frame events in such a way that the relevance of the principles to the solutions of particular problems is made clear, and learning how to code events in such a way that the principles can actually be applied to the events.”^25^^(p11)^

Although the brief exposure to relevant cases in the experience group led to no advantage over a control condition, this finding may reflect an inadequate experience base rather than a failure of the concept underlying the intervention. There is evidence that exposure to multiple cases is sufficient to yield approximately correct posttest probability estimates.^26,27,28,29,30^ Rottman^29^ suggested that physicians’ posttest probability judgments are strongly and appropriately associated with their beliefs about the value of specific tests. Weber et al^30^ have shown an association between increasing expertise and improvement of clinicians’ estimates of probability. Although clinicians may not be aware of terms such as *prior probability* or *likelihood ratio*, their probability revisions are reasonably consistent with an analytic revision based on these parameters. Koehler^20^ has suggested that the base-rate fallacy and other cognitive biases may be oversold and that people may be better in real-world situations where they are more sensitized to the task at hand and the aims of the problem-solving exercise. Experience in real-world settings may be the best teacher, but our study suggests that formal teaching of statistical heuristics and concepts can provide significant improvement in bayesian updating among novice clinicians.

### Limitations

The trial has limitations. Despite the statistically significant advantage of the concept intervention, the differences among the conditions were modest. This finding may reflect the fact that the interventions were limited to an 18-minute video presentation and a relatively small number of examples. It is possible that a more intense or repetitive educational intervention would have shown a more substantial difference. In addition, the study was limited by a relatively small population, which may have lessened our ability to show a significant effect of repeated examples, compared with control participants. Also, the small number of assessment questions used to compare learned and new cases may have been insufficient to show a statistically significant effect. In addition, our analysis was limited to short-term effects, and we did not analyze whether the improvement in the concept or experience group was persistent over the long term.

## Conclusions

We tested whether formal instruction on concepts of bayesian reasoning or repeated examples improves students’ performance at estimating posttest probability. Our study showed an advantage for students who received theoretical instruction on bayesian concepts. However, the advantage was relatively modest, and all participants performed surprisingly well in estimating posttest probability in this study. Our findings have implications for how to teach diagnostic reasoning to novice clinicians.

## Notes

Supplement 1. **Trial Protocol**

Supplement 2. **eAppendix.** Content Provided to Participants and Test Questions

Supplement 3. **Data Sharing Statement**
