# Supplementary material for: Effect of Teaching Bayesian Methods Using Learning by Concept vs Learning by Example on Medical Students’ Ability to Estimate Probability of a Diagnosis: A Randomized Clinical Trial
Source: JAMA Netw Open. 2019 Dec 20;2(12):e1918023. doi: 10.1001/jamanetworkopen.2019.18023 (PMC7027434; doi:10.1001/jamanetworkopen.2019.18023)
Supplement: Supplement 1. — Trial Protocol [file jamanetwopen-2-e1918023-s001.pdf]

Hamilton Integrated Research Ethics Board

General Research Application

Overview

F.1 Is this application a:

- ○ New Application
- ● Post Provisional Response

F.2 Study Type

- ○ Clinical Trial
- ● Other Minimal Risk

F.3 Application Type

- ● Original
- ○ Reciprocal

Study

1.1 Short Study Title (Max 25 Characters)

Teaching Use of Diagnostic Tests

1.2 Title of Study

A Study to Evaluate Strategies for Teaching Effective Use of Diagnostic Tests

1.3 Study Acronym

EBM

1.4 What is your expected study period?

Start Date: Sep-01-2017 End Date: Mar-31-2018

1.5 Has this study undergone a formal scientific review?

- ○ Yes
- ● No

1.6 Has this study been submitted to any other REB?

- ○ Yes
- ● No

1.7 Has this study been denied approval by any other REB

- ○ Yes
- ● No

1.8 Is this study an:

- ○ Industry Sponsored Study
- ● Investigator-initiated Study

1.9 Is this a student project?

- ○ Yes
- ● No

1.10 Is this a multi-site study?

- ● Yes
- ○ No

1.11 Do you plan on conducting this study at both St. Joseph's (SJHH) and Hamilton Health Sciences (HHS)?

- ○ Yes
- ● No

1.12 How will you make the results of this study public?

- [x] Peer-reviewed publication
- [ ] Clinical trial registry
- [ ] Thesis
- [ ] Presentation
- [ ] Report to participants
- [ ] Other

1.13 How would you explain this study to a lay person (max 10 lines)?

Clinical reasoning, arriving at a diagnosis, involves two kinds of knowledge; the formal knowledge that students learn in medical school and the experiential knowledge that they acquire as working professionals. It is not clear which kind of knowledge is more critical in diagnostic skill. This study directly contrasts instruction teaching students mathematical approaches to using a diagnostic test (Bayes Theorem) to simply acquiring examples of cases when the test was used.

Investigators

2.1 Local Principal Investigator

Specify LPI site affiliation: McMaster

Salutation	First Name	Surname
Dr.	Geoff	Norman

Degree: PhD   Institution: McMaster

Dept: Health Research Methods, Evidence, and Impact (HEI)

Division: Please Select...

Address: DBHSC 5003

City: Hamilton   Prov: ON   PC: L8P1H6

Tel & Ext.: 905 5259140 ext 22119

Fax: Email: norman@mcmaster.ca

2.2 Please indicate the research institute or group the LPI is affiliated with:

Other

Specify Other Institute:

Program for Educational Research and Development

2.3 Is the LPI the Principal Investigator (PI) of this study?

- (●) Yes
- ( ) No

2.4 Does this study have a Coordinator?

- ( ) Yes
- (●) No

2.5 Does this study have any Co-Investigators?

- (●) Yes
- ( ) No

Are any Co-Investigators a student:

- ( ) Yes
- (●) No

Co-Investigators:

Salutation	First Name	Surname
Dr.	Jonathan	Sherbino

Degree	MD	Institution	Ottawa
Dept	Medicine		
Division	Medicine-Emergency Medicine		
Address	DBHSC 5003		

City	Hamilton	Prov	ON	PC	L8P1H6

Tel & Ext.	905 5259140, ext 20061		
Fax		Email	sherbino@gmail.com

Salutation	First Name	Surname
DR.	John	Brush

Degree	MD	Institution	Eastern Virginia Medical School
Dept	Medicine		
Division	Please Select...		
Address	844 Kempsville Dr.		

City	Norfolk	Prov	Virginia	PC	23502

Tel & Ext.	757-261-0700		
Fax		Email	jebrush@me.com

Investigator Training

2.7 Please attach either a copy of LPI's:

- (selected) TCPS2 (CORE) tutorial certificate
- GCP certificate

Type	Name	File Name	Date	Version	Size
TCPS2 CORE Certificate	TCPS2 Norman	TCPS2 Norman.pdf	Aug-15-2017 12:00:00 AM	1.0	890.6 KB

TCPS2 Certificate Date: Oct-30-2015

Locations

3.1 Where will this study take place? Check all that apply:

Hamilton Health Sciences (HHSC)

- ☐ Chedoke
- ☐ Hamilton General (HGH)
- ☐ Juravinski Hospital (JHCC)
- ☐ Juravinski Cancer Centre (JCC)
- ☐ McMaster Children's Hospital (MCH)
- ☐ McMaster University Medical Centre (MUMC)
- ☐ St. Peter's Hospital
- ☐ West Lincoln Hospital

St. Joseph's Healthcare Hamilton (SJHH)

- ☐ Charlton Campus
- ☐ West 5th Campus
- ☐ King Campus

- ☐ McMaster University

- ☐ Community

- ☑ Other

Please specify Other location:

Study participants will be administered materials online at a time and location of their choosing

3.2 Does this study take place at sites outside of Hamilton Health Sciences, St. Joseph's Healthcare Hamilton or McMaster University?

- ◉ Yes
- ○ No

3.3 Please select all countries that apply

United States

3.4 Setting:

- ☐ Emergency
- ☐ ICU
- ☐ Outpatient
- ☐ Inpatient
- ☑ Other

Specify Other:

Web-based administration to students at Eastern Virginia Medical School

Description

4.1 Is this a clinical trial?

- ○ Yes
- ◉ No

4.2 Is this an observational study?

- ○ Yes
- ◉ No

4.3 Is this an epidemiological study

- ○ Yes
- ◉ No

4.4 Does this study include human tissue collection or analysis?

- ○ Yes
- ◉ No

4.5 Does this study include genetic testing?

- ○ Yes
- ◉ No

4.6 Does this study require access to existing records?

- ○ Yes
- ◉ No

4.7 Does this study involve qualitative methods?

- ○ Yes
- ◉ No

4.8 Does this study involve any other types of research?

- (●) Yes
- ( ) No

If yes, please explain:

Experimental study of two educational interventions with quantitative outcome (accuracy)

Methodology

6.1 This section is intended to be a summary. Please submit a study protocol detailing the research that you plan to conduct.

Type	Name	File Name	Date	Version	Size
Protocol	Brush, Sherbino Norman EBM instruction	Brush, Sherbino Norman EBM instruction.docx	Aug-15-2017 12:00:00 AM	1.0	159.0 KB

6.2 What is the rationale for this study (i.e., why are you doing this study; max 5 lines)?

To determine whether formal instruction in mathematical streets to incorporate diagnostic test results and more or less effective than exposure to representative cases in improving diagnostic accuracy

6.3 What are the objectives of this study (i.e., what do you hope to show; max 5 lines)?

Whether instruction in formal or experiential use of diagnostic tests is more effective

6.4 Please specify your study design (e.g., RCT, cohort; max 5 lines):

Three group randomized experiment

6.5 Please specify your study population (e.g., diagnosis, age, gender; max 5 lines):

Senior preclinical medical students

6.6 Please specify your study procedures (max 5 lines):

Students will receiver either a) 45 minute of formal instruction on using diagnostic tests or b) 45 min of relevant clinical cases or c) 3 passages from a clinical text related to each of the 3 conditions in the study and asked to study them for 15 min each.

6.7 What is your primary outcome and how will it be measured (max 5 lines)?

Accuracy on diagnosing 20 new written cases of the same diagnoses expressed as subjective pretest and post-test probabilities and compared with a formal calculation

6.8 What are your secondary outcomes and how will they be measured (max 5 lines)?

none

6.9 What is your sample size?

Local: 60

Total (include all sites in study): 120

6.10 How did you determine your sample size (max 5 lines)?

Similar studies of interventions use similar sample sizes. No previous specific data are available so no point in attempting formal sample size calculation

6.11 How will you analyze your data (max 5 lines)?

mixed model ANOVA

Study Interventions

7.1 Does this study involve any diagnostic testing?

- ○ Yes
- ◉ No

7.2 Does this study involve any of the following interventions?

- ☐ Chemotherapy
- ☐ Radiotherapy
- ☐ Gene therapy
- ☐ Cognitive/Behavioural Therapy
- ☐ Drugs
- ☐ Natural Health Product
- ☐ Surgery
- ☐ Medical Device
- ☐ Observation
- ☑ Questionnaire/Survey
- ☐ Focus Group
- ☐ Interview
- ☐ Exercise
- ☐ Other

7.3 Does this study require any drugs?

- ○ Yes
- ◉ No

Safety and Monitoring

8.1 How will you monitor the conduct of this study (max 5 lines)?

> Single intervention, lasting about 1.5 hours. NO monitoring required

If this is a minimal risk Undergraduate/Master's study, the student's Supervisor should serve as monitor.

8.2 Does this study have a formal steering committee?

- ○ Yes
- ◉ No

8.3 Will an interim data analysis be done?

- ○ Yes
- ◉ No

8.4 Will you use a data safety monitoring board (DSMB)?

- ○ Yes
- ◉ No

8.5 Is there a likelihood of incidental findings resulting from your research?

- ○ Yes
- ◉ No

Risks and Benefits

9.1 What are the risks to participants in this study (e.g. pain, distress, privacy breach, social implication; max 5 lines)?

> Time inconvenience. Since they are not expected at this stage to have learned any of the study materials, virtually no other risk.

9.2 How will you minimize and manage the risks (max 5 lines)?

> not applicable

9.3 Will participants receive any other benefits from participating in this study (e.g. continued access to new drug)?

- (x) Yes
- ( ) No

Please explain

They will be learning some useful knowledge that will eventually be part of their formal education later in clerkship

9.4 Will participants be reimbursed for study related expenses (e.g. parking)?

- ( ) Yes
- (x) No

9.5 Will participants receive any compensation (e.g. money for time)?

- ( ) Yes
- (x) No

9.6 How will the scientific community and society benefit from this study (max 5 lines)?

Improved diagnostic accuracy through validated educational interventions

Participants

10.1 Does this study focus on any of these vulnerable groups? Check all that apply.

- [ ] People with cancer
- [ ] People with incurable disease
- [ ] People in medical emergencies
- [ ] People in long-term care
- [ ] People with mental health issues
- [ ] People who are unable to consent
- [ ] Children
- [ ] Pregnant females
- [ ] Elderly people
- [ ] Aboriginal people
- [ ] People in poverty
- [ ] People in prison
- [ ] Other

10.2 Do you have any age, ethnicity, language, gender or race-related inclusion or exclusion criteria?

- ( ) Yes
- (x) No

Recruitment

11.1 How do you plan to recruit participants?

- [ ] Investigators will approach their own patients/students
- [ ] Investigators will receive referrals from other Healthcare providers
- [ ] Decision support services (DSS) will prepare a list of potential participants. DSS signature requried for HHS
- [x] Advertising (e.g., poster, email, flyers, newspaper, web-based, etc.)
- [ ] Database of people who consented to future contact
- [ ] Direct approach (e.g. random digit dialing)
- [ ] Educational records (e.g. information from Registrar)
- [x] Other

Please submit a copy of all advertisements

Type	Name	File Name	Date	Version	Size
Recruitment and Advertising Materials	Email Recruitment 1.0 8-15-17	Email Recruitment 1.0 8-15-17.docx	Aug-15-2017 12:00:00 AM	1.0	25.3 KB

You selected:

Other

Please specify

Students will receive an email on university account describing study and asking for their participation

11.2 Do you need to screen Personal Health Information (PHI) of patients to identify potential participants?

- ( ) Yes
- (•) No

11.3 Does your recruitment plan require you to contact potential participants by telephone, email or letter?

- (•) Yes
- ( ) No

- [ ] Telephone
- [x] Email
- [ ] Letter

Please attach a copy of all telephone scripts and correspondence

Type	Name	File Name	Date	Version	Size
Scripts & Correspondence	Email Recruitment 1.0 8-15-17	Email Recruitment 1.0 8-15-17.docx	Oct-17-2017 12:00:00 AM	2	25.6 KB

Consent

12.1 Will you be seeking written consent from participants (i.e. age 16+)?

- [x] Yes
- [ ] No

Please attach all Consent form for Participants

Type	Name	File Name	Date	Version	Size
Consent form for Participants	Consent 1.0 8-15-17	Consent 1.0 8-15-17.docx	Oct-17-2017 12:00:00 AM	2	191.6 KB

12.2 Will all participants be competent to consent?

- [x] Yes
- [ ] No

12.3 Will the participants be minors under the age of 16?

- [ ] Yes
- [x] No

12.5 Are there any other study related consent forms

- [ ] Yes
- [x] No

12.6 Do you need to request a waiver of consent?

- [ ] Yes
- [x] No

12.7 Who will obtain consent to participate?

N/A see below

12.8 When and where will this be done?

It will be done online as part of the intervention

12.9 Will any of the investigators have a position of authority or power over the participants?

- [ ] Yes
- [x] No

12.10 How will you ensure continuing consent during the study?

One shot intervention

12.11 Will participants have the option to withdraw from this study?

- [x] Yes
- [ ] No

What do they have to do to withdraw?

Exit from computer program

Personal Information

Categories of information (TCPS)

- Identifying information identifies a participant through direct identifiers (e.g. Full name, Medical record number)
- Identifiable information could identify a participant through a combination of indirect identifiers (e.g. DOB plus address)
- De-identified/coded information: identifiers are removed and replaced with a code; the code can be used to re-identify participants
- Anonymized information: all identifiers are removed and no code is kept
- Anonymous information: no identifiers were collected

Personal Health Information (PHI)

- The collection, use and disclosure of PHI are regulated by the Personal Health Information Protection Act (PHIPA) 2004. Researchers must comply with this legislation
- Collection of participant SIN is prohibited, unless payments to participant exceed $500/yr (required for tax purposes)
- PHI should be collected at the lowest level of identifiability possible (e.g. initials instead of a name, age instead of DOB)

13.1 Do you need to record any identifiers for this study?

- ○ Yes
- ● No

13.2 How will you record study data?

- ☐ Case Report Form (CRF). Please attach the first 2 data pages.
- ☐ Data Collection Sheet (DCS)
- ☑ Other

Please specify

Automatic collection of anonymous responses from Livecode software

Storage and Protection of Information

PHIPA requirements

- Paper files with identifiable information must be kept in a locked cabinet within a locked office (but not at home)
- Electronic files with identifiable information may be stored on a password protected computer on a secure network (i.e., virus protection, file backup, firewall) or they must be encrypted.
- Electronic files with identifiable information may be stored on mobile devices (e.g. laptop, CD, USB, PDA), but only if there is no alternative method of storage; these files must be encrypted.
- Identifying and/or identifiable PHI cannot be transmitted by email unless it is encrypted

Coding

- Identifying and/or identifiable PHI should be protected by a coding system
- The code (study ID and identifiable PHI) must be isolated from study data and stored in a secure manner

14.1 Will you use a coding system to protect identifiable information?

- ( ) Yes
- (•) No

Please explain

There is no identifiable information captured by computer

14.2 How will you store and protect the study code (or other data with identifiers)?

Type of record		Required protection	Location (i.e. bldg, room)
Paper file	☐	Locked cabinet in locked institutional office	
Electronic file	☑	Password protected computer on a secure network	DBHSC
Electronic file	☐	Encrypted - Program Name	
AV tapes	☐	Locked cabinet in locked institutional office	

14.3 How will you store and protect data without identifiers?

password protected file in locked office

14.4 Do you plan to anonymize the study data?

- (•) Yes
- ( ) No

When?

At capture

14.5 How long will you keep the study data?

2 years

14.6 What will you do with the study data after this period?

delete

Transmission of Data

15.1 Does this study require you to send data outside of the institution where it is collected?

- (•) Yes
- ( ) No

15.2 Does this data include identifiers?

- ( ) Yes
- (•) No

A data transfer agreement is necessary (contact your institutional Agreements/Contracts official in the Research Administration office for details)

15.3 Where will the data be sent?

Data captured at EVMS will be automatically captured on our server as Excel spreadsheet

Data sent to the US is open to access by US Regulatory Bodies. Researchers must inform study participants of this possibility.

15.5 How will the data be transmitted?

- [ ] Fax
- [ ] Email (Encryption protocol must be attached)
- [ ] Private Courier (Delivery must be traceable)
- [ ] Canada Xpresspost (Regular mail may not be used)
- [x] Other

Please specify

See above

Secondary Use of Data

16.1 Will you link the locally collected data with any other data sets?

- ( ) Yes
- (•) No

16.2 Will the data be entered into a database for future use?

- ○ Yes
- ◉ No

Funding

17.1 Does this study require any financial or in-kind support?

- ◉ Yes
- ○ No

Please identify the sources (include all internal, external, public or private sources)

Funding Source	Sponsor funding ref. no.	Local Budget	Total Budget	Status
Internal funds		$1000	$1000	Pending

Please provide the study title on the funding Application

A Study to Evaluate Strategies for Teaching Effective Use of Diagnostic Tests

17.2 Where will the funds be administered?

- ☐ SJHH
- ☐ HHS
- ☑ McMaster Faculty of Health Sciences
- ☐ Other

17.3 Did the research proposal for this funded project originally include human participants or use of their biological material and/or access to their records?

- ◉ Yes
- ○ No

17.4 Will there be a signed contract/agreement with a study-related funding source?

- ○ Yes
- ◉ No

Conflict of Interest

1 Will any investigators, members of the research team, and/or their partners or immediate family members:

• Function as an advisor, employee, officer, director or consultant for a study-related sponsor or funding source?

- ○ Yes
- ● No

• Have a direct or indirect financial interest (including patents or stocks) in the drug, device or technology employed in this research study?

- ○ Yes
- ● No

• Receive any personal benefit (apart from fees for service) as a result of, or connected to this study? (e.g., remuneration, intellectual property rights, rights of employment, consultancies, board membership, share ownership, stock options, honorariums).

- ○ Yes
- ● No

Budget

Do you have a detailed study budget?

- ○ Yes
- ● No

- Please check all services required in your study and enter amounts where possible
- If you report your budget as cost per participant, please provide a list of services/costs per participant
- Payments to investigators should not exceed accepted standards (e.g., OHIP)
- Investigators may not accept any payments for enrolling participants

Services	A (Unit cost)	B (# per participant)	C (# of participants)	(AxBxC) = Subtotal
☐ X-ray				
☐ Ultrasound				
☐ Bone Scan				
☐ CT Scan				
☐ MRI/PET				
☐ ECG				
☐ Endoscopy				
☐ Labs				
☐ Pharmacy (e.g. drugs, fees)				
☐ Other				

Personnel	A (Unit cost)	B (# per particpant)	C (# of participants)	(AxBxC) = Subtotal
☐ Investigator (e.g. history)				
☐ Nurse/ coordinator				
☐ Other staff				

Participants	A (Unit cost)	B (# per particpant)	C (# of participants)	(AxBxC) = Subtotal
☐ Reimbursement (e.g. parking)				
☐ Payment (e.g. money for time)				

Equipment (please specify) **Subtotal**

Administration (please specify) **Subtotal**

Other (please specify) **Subtotal**

Cost for programming the software est. $1000 — 3000

	Subtotal
**Industry Studies (add 30% overhead)**	
**Industry Studies (add $3500 REB fee)**	
**Total**	1000

Supporting Documents

1. Investigator Brochures
2. Product Monograph
3. Questionnaires
4. Patient Diary
5. Other Documents
6. Cover Letter/Summary of Changes Documents

Authorization

21.1 Is your study being conducted at:

- [x] McMaster University or one of the Hamilton Health Sciences sites
- [ ] St. Joseph's Healthcare Hamilton

Resource Authorization Post Provisional Response

21.2 Do you require authorization signatures that you have not previously submitted?

- ( ) Yes
- (•) No

21.3 Do you require any departmental resource/authorization signatures that were not previously submitted?

- ( ) Yes
- (•) No

Signatures for Qualification and Resource Verification

21.7 Confirmation of LPI Qualifications: Chief (or) Chair of Department (or) Professional Practice Leader

For the Schools of Nursing and Rehab Science the designated Research representative may sign on behalf of the Chair.

- I confirm that the LPI has the credentials and expertise to conduct this research
- I confirm that the LPI is a member in good standing at HHS and/or McMaster University and/or St. Joseph's Hospital Hamilton

Confirmation of Resources

Investigator Signatures

21.5 Confirmation of Responsibility- Local Principal Investigator

- I assume full responsibility for the scientific and ethical conduct of the study as described in this REB application and submitted protocol.
- I agree to conduct this study in compliance with the Tri-Council Policy Statement (TCPS) and any other relevant regulations and guidelines.
- I certify that all researchers and other personnel involved in this project at this institution are appropriately qualified and experienced or will undergo appropriate training to fulfill their role in this project.
- I certify that any and all conflicts of interest have been declared
- I have obtained all necessary resource utilization signatures, and all costs associated with the use of these resources have been declared.
- On behalf of my research team, I recognize the importance of maintaining the confidentiality of all personal information, including personal health information, and the privacy of individuals with respect to that information. I will ensure that the personal information is used only as necessary, to fulfill the specific research objectives and related research questions described in this application and approved by the REB. This includes all conditions and restrictions imposed by the REB governing the use, security, disclosure, return or disposal of the research participants' personal information. I agree to take any further steps required by the REB and/or the institution to ensure that the confidentiality and security of the personal information is maintained in accordance with the Personal Health Information Protection Act (PHIPA), its accompanying regulations and the TCPS.

LPI Signature

Signed: This form was signed by Professor Geoff Norman (norman@mcmaster.ca) on Oct-24-2017 13:20

EVMS
Eastern Virginia Medical School

May 15, 2018

John Brush, M.D.
Sentara Norfolk General Hospital
600 Gresham Drive
Norfolk, VA 23507

IRB # 18-04-EX-0062

Dear Dr. Brush:

This form provides additional information to the *Application for Approval of Research Involving Human Subjects* form that accompanies this letter. The Application is the official document that confirms IRB review and type of approval and includes the IRB#, study title, and an appropriate chair, vice-chair or IRB member signature.

☒ IRB Study Title: A Study to Evaluate Strategies for Teaching Effective Use of Diagnostic Tests (EBM)

- Protocol: A Study to Evaluate Strategies for Teaching Effective Use of Diagnostic Tests Version Date: May 11, 2018

☒ No sponsor has been identified as providing funding for this study or project.

☒ The IRB has waived the requirement for the investigator to obtain a signed consent for all subjects using the following criteria:
☒ The research presents no more than minimal risk and involves no procedures for which written consent is normally required outside of the research context. [45CFR46.117(c)(2)]
Because the documentation requirement is waived, the IRB is requiring the investigator to provide all subjects with a written statement regarding the research.

☒ Instruments: Case examples

☒ Data Collection Tool

☒ Advertising: Recruitment email

☒ Additional Materials: Request for waiver of documentation of informed consent; Instructional video

This approval is a result of an **Expedited Board** action that specified the following category/categories under 63FR 60364 dated November 9, 1998:

☒ (7) Research on individual or group characteristics or behavior (including, but not limited to, research on perception, cognition, motivation, identity, language, communication, cultural beliefs or practices, and social behavior) or research employing survey, interview, oral history, focus group, program evaluation, human factors evaluation, or quality assurance methodologies.

☒ This study was approved on **May 11, 2018** and may be initiated now that you are in receipt of Final Approval documents.
- IF YOU ARE CONDUCTING YOUR RESEARCH AT ONE OF THE LOCAL HOSPITALS, YOU MUST RECEIVE THE APPROPRIATE APPROVALS FROM THAT HOSPITAL BEFORE INITIATING YOUR STUDY.
- IF YOU ARE CONDUCTING YOUR RESEARCH AT A SITE OTHER THAN EVMS, YOU ARE RESPONSIBLE FOR OBTAINING ANY LOCAL REVIEW NECESSARY FOR THE CONDUCT OF THIS RESEARCH.

☒ **Your protocol expiration date is May 10, 2019. Please see the attached form for the due date of the next continuing review submission.**

☒ Please remember that prompt reporting to the IRB of proposed changes in a research activity (e.g., changes to the protocol, consent form(s), advertisements, or other study-related materials) is required. This includes information related to funding sources. In addition, the changes

HUMAN SUBJECTS' PROTECTIONS PROGRAM
721 FAIRFAX AVENUE, ANDREWS HALL, SUITE 128
NORFOLK, VIRGINIA 23501
TEL 757.446.8423
FAX 757.624.2275
www.evms.edu

must be reviewed and approved by the EVMS IRB ***before*** the changes can be initiated *except* when it is necessary to eliminate apparent immediate hazards to the subject.

Eastern Virginia Medical School (EVMS) has a Federalwide Assurance (FWA 00003956) from OHRP. The Institutional Review Boards (IRB 00000460 and IRB 00001345) are registered with OHRP and are in compliance with 45 CFR 46, 21 CFR 50, and 21 CFR 56.

Please reference the IRB number, principal investigator and study title in any correspondence regarding this protocol.

Thank you for your continued cooperation with the Institutional Review Board.

Sincerely,

Daniel Sullivan, PhD, CIP
IRB Assistant Director

DMS/acl

EASTERN VIRGINIA MEDICAL SCHOOL

APPLICATION FOR APPROVAL OF RESEARCH INVOLVING HUMAN SUBJECTS

EVMS Institutional Review Board

Instructions: Please submit this form to the IRB Office, attaching the IRB protocol, abstract, data collection instruments, consent forms and/or informational letters, letters of approval from agencies, hospital impact statement(s) and other supporting documents.

- ~ HANDWRITTEN DOCUMENTS WILL NOT BE ACCEPTED BY THE IRB OFFICE.
- ~ ALL DOCUMENTS INCLUDED IN THE SUBMISSION MUST BE PAGINATED.

HELP: If you are unsure how to complete a field, press F1 while on the field and a help box will appear.

IRB Number: (If assigned)	18-04EX-0062

ADMINISTRATIVE INFORMATION			
**Study Title:**	A Study to Evaluate Strategies for Teaching Effective Use of Diagnostic Tests (EBM)	**Date Submitted:** (IRB USE ONLY) RECEIVED MAY 11 2018 By	
**Principal Investigator:**	John Brush, MD		
**PI Dept / Address**	Sentara Cardiology Specialists<br>Sentara Heart Hospital<br>600 Gresham Drive		
**City / State / Zip**	Norfolk, VA 23507		
**Phone Number(s):**	(757) 261-0700	**E-Mail:**	jebrush@me.com
**Person Preparing This Submission**			
**Name:**	Brittany McMichael	**Role:**	Research Team Member
**Address:**	Sentara Cardiovascular Research Institute<br>600 Gresham Drive<br>Norfolk, VA 23507		
**Phone Number(s):**	757-388-5487	**E-Mail:**	bymcmich@sentara.com

INVESTIGATORS AND/OR RESEARCH TEAM MEMBERS

Name	Department	Address	Status	HIPAA for Research Training Date	Human Subjects Protection Training Date
John Brush, MD	Sentara Cardiology Specialists	600 Gresham Drive<br>Norfolk, VA 23507	Research Team Member	on file	on file
Judith Taylor-Fishwick	EVMS Faculty Affairs & Professional Development	721 Fairfax Avenue<br>Norfolk, Virginia 23507	Research Team Member	on file	on file
Brittany McMichael	Sentara Cardiovascular Research Institute	600 Gresham Drive<br>Norfolk, VA 23507	Research Team Member	on file	on file
Allison Knight, PhD	Eastern Virginia Medical School	Lewis Hall<br>700 W. Olney Road<br>Norfolk, VA 23507	Research Team Member	on file	on file
			-- Choose One --		
			-- Choose One --		
			-- Choose One --		

			-- Choose One --		
			-- Choose One --		
			-- Choose One --		
			-- Choose One --		

1. TYPE OF REVIEW: Review the sub-categories and check the appropriate box (check only one)

☐ **FULL BOARD REVIEW:** (A $1,500 review fee is charged unless a "Wavier of IRB Fee" form is submitted with this application and approved by the Office of Research Subjects Protections.)

CLICK HERE AND PRESS F1 FOR NUMBER OF COPIES TO SUBMIT: ▶

☒ **EXPEDITED REVIEW:** Insert the Category number below that supports the type of review: 7

CLICK HERE AND PRESS F1 FOR NUMBER OF COPIES TO SUBMIT: ▶

(1) Clinical Studies of drugs or devices when: [1a] Drugs: IND not required; [1b] Devices: IDE not required.
(2) Collection of blood samples. CLICK HERE AND PRESS F1 FOR GUIDANCE: ▶
(3) Prospective collection of biological specimens for research purposes by noninvasive means.
(4) Collection of data through noninvasive procedures routinely employed in clinical practice, excluding procedures involving x-rays or microwaves.
(5) Research involving materials that have been collected, or will be collected solely for non-research purposes.
(6) Collection of data from voice, video, digital, or image recordings made for research purposes.
(7) Research on individual or group characteristics or behavior or research employing survey, interview, oral history, focus group, program evaluation human factors evaluation, or quality assurance methodologies.

☐ **EXEMPT REVIEW:** Insert the Category number below that supports the type of review: -- Choose One --

CLICK HERE AND PRESS F1 FOR NUMBER OF COPIES TO SUBMIT: ▶

(1) Research in Educational Setting involving normal educational practices.
(2) Educational Tests, Survey Procedures, Interview Procedures, or Observe Public Behavior unless subjects can be identified and disclosure place subjects at risk of criminal & civil liability. **[Does not apply to those <18 years old. Therefore, defaults to expedited or Full Board review.]**
(3) Educational Tests, Survey Procedures, Interview Procedures, or Observe Public Behavior unless subjects elected/appointed officials or candidates for public office and Federal statute requires maintenance of confidentiality. **[Does not apply to those <18 years old. Therefore, defaults to expedited or Full Board review.]**
(4) Collection/Study of Existing Data, Documents, Records, Pathological/Diagnostic Specimens and Subjects Cannot Be Identified. CLICK HERE AND PRESS F1 FOR GUIDANCE: ▶
(5) Federal Dept/Agency Research & Demonstration projects.
(6) Taste & Food Quality Evaluation & Consumer Acceptance Studies.

2. REQUIRED TRAINING:

It is necessary for all investigators, co-investigators, and research team members to complete human subjects protection training in order to receive IRB approval to proceed with research using human subjects, their data, or biological samples. Training opportunities and requirements can be found on the Office of Research web site at http://www.evms.edu/research/office/index.html.

Contact the Office of Research at (757) 446-8480 for additional information on all research training requirements.

Please note that Bloodborne Pathogen Training is mandated annually for **EVMS faculty and staff** with potential exposure to blood/body fluid by the Occupational Safety and Health Administration (OSHA).

Contact the Occupational Health Department at 446-5870 for additional information.

3. FINANCIAL STATEMENT:

Have you, other family members or any other person responsible for the design, conduct, or reporting of this research received from the sponsor (or a subsidiary or parent company of the sponsor):	Choose one answer in each row below:	
Salary, other payments for services (e.g., consulting fees or honoraria), recruitment bonuses, trips, referral fees or other incentives **that are NOT covered by an EVMS grant, contract, or clinical agreement?**	☒ No	☐ Yes
Equity interests (e.g., stocks, stock options, or other ownership interests greater than 3% ownership or greater than $10, 000 per annum of salary, fees, or other continuing payments)?	☒ No	☐ Yes
Intellectual property rights (e.g., patents, copyrights and royalties from such rights)?	☒ No	☐ Yes
**If "yes," to any of the above**, please provide a written explanation of the situation in this box. You may also be required to submit information to the EVMS Conflict of Interest (COI) Committee through the Office of Research, 446-8480. Refer to Appendix C for Model Language to insert into the consent form.		

4.	THIS STUDY WILL BE ACTIVE AT THE FOLLOWING LOCAL SITES: (Be sure to list site for ALL phases of the research)	
☐ Bon Secours DePaul Medical Center	☐ Bon Secours Maryview Hospital	☐ Children's Hospital of The King's Daughters
☐ Children's Specialty Group	☐ Devine Tidewater Urology	☒ Eastern Virginia Medical School
☐ Sentara Bayside Hospital	☐ Sentara CarePlex Hospital	☐ Sentara Leigh Memorial Hospital
☐ Sentara Norfolk General Hospital	☐ Shore Health Services	☐ Virginia Oncology Associates

(4a.) Other local or international site for this IRB application (specify name and include the complete address):	TYPE
	-- Choose One --
	-- Choose One --
	-- Choose One --
	-- Choose One --

5.	OTHER SITES:
In addition to the local sites listed above, is this study also conducted at any national or international sites?	☐ No ☒ Yes

6. TYPES OF PARTICIPANTS (CHECK ALL THAT APPLY):		
☐ Children [specify age range(s)]:	☒ Adults [specify age range(s)]: 18-89<br>(NOTE: Adults 90 or older must be grouped into one category per HIPAA regulations.	
☒ Students/Employees	☐ Healthy Volunteers	☐ Critically Ill Patients
☐ Cognitively Impaired Individuals	☐ Subjects in Emergency Conditions	☐ Economically Vulnerable Subjects
☐ Pregnant Women	☐ Fetus(es)	☐ In vitro fertilization
☐ Medical Records	☐ Specimens (blood, tissue)	
☐ Other: (specify):		

7. SOURCE OF SUBJECTS: (CHECK ALL THAT APPLY):		
☐ My Practice	☐ Referral from Other Physicians	☐ Medical Records
☐ Outpatients/Clinics	☐ Stored/Banked Human Specimens	☒ Other, Explain in Protocol
NOTE: All advertisements or other materials used to recruit subjects must be submitted for IRB approval.		

8.	CONSENT PROCEDURES: (CHECK ALL THAT APPLY):
**8a. CONSENT TO BE OBTAINED FROM:**	**8b. CONSENT TO BE OBTAINED BY:**
☒ Patient/Subject	☒ Principal investigator
☐ Parent(s)/Guardian	☐ Co-investigator(s)
☐ Legally authorized representative	☐ Research Team Members not on protocol (list below)
☐ Assent to be obtained from subjects age to	

8c. List others not identified in the protocol who are qualified and authorized to obtain subject consent (e.g., study coordinators, clinical staff, etc.) Any individual listed in this section must meet all appropriate EVMS training requirements.

NAME:	RELATIONSHIP TO THE STUDY:	LIST ALL SPECIFIC QUALIFICATIONS TO CONDUCT THE INFORMED CONSENT PROCESS:
	-- Choose One --	
	-- Choose One --	
	-- Choose One --	
	-- Choose One --	
	-- Choose One --	
	-- Choose One --	

8d. WITNESS: In most cases, a witness signature is not required unless consent is obtained orally. If a witness signature is preferred by the investigator or sponsor, please explain below and include the appropriate signature box on the subject consent form(s).

N/A

9.	WAIVER REQUESTS (CHECK ALL THAT APPLY):		
	Are you requesting that the IRB waive the requirements for obtaining subject consent for this study?<br>If yes, an ***"Application for Waiver of Consent"*** must be completed and attached to ALL copies of the submission. **ALL REQUESTS FOR WAIVER OF SUBJECT CONSENT ARE REVIEWED BY THE FULL BOARD.**	☒ No	☐ Yes
	Are you requesting that the IRB allow access to or the use of Protected Health Information (PHI) without obtaining subjects permission?<br>If yes, an ***"Application for Waiver of Authorization for the Use of Protected Health Information (PHI)"*** must be completed and attached to ALL copies of the submission	☒ No	☐ Yes

10. SUBJECT PARTICIPATION: *All items must be answered. If applying for a medical record review, length of active participation and follow-up should be answered as "Not Applicable".

ITEM	INSERT LENGTH OF TIME, NUMBER OR DATE
Length of time for active participation (as defined in protocol)	90 minutes
Follow-up (long-term follow-up after study completion)	None
Number of local subjects or medical records or samples	60
Total number of subjects or records or samples across all sites	120
Duration of study at this local site	6 months

Anticipated Start Date the proposed study will begin (be sure to allow time for IRB review and approval):	May 1, 2018<br>**Month / Year**
Anticipated End Date of the proposed study	December 31, 2018<br>**Month / Year**

**11.**	**ARE THE FOLLOWING ASSOCIATED WITH THE RESEARCH STUDY?**			
	**11a. SUPPLEMENTARY DOCUMENTS INCLUDED:**			
☐	Subject Diary	☒ No	☐ Yes	If Yes, insert identifier:
☒	Questionnaire or Psychological Instrument	☐ No	☒ Yes	If Yes, insert identifier: Email Recruitment
☐	Federal NIH Grant Application	☒ No	☐ Yes	If Yes, insert identifier:
☐	Investigator Brochure	☒ No	☐ Yes	If Yes, insert identifier:
☐	Drug Package Insert	☒ No	☐ Yes	If Yes, insert identifier:
☒	Data Collection Tool (with a key to all field headings)	☐ No	☒ Yes	If Yes, insert identifier:
☒	Advertisements / Flyers / Patient Information Sheets	☐ No	☒ Yes	If Yes, insert identifier: Email Recruitment, Analytical Lecture, Case study samples
☒	Other, Please Explain: Wiaver of Documentation of Informed Consent	☐ No	☒ Yes	If Yes, insert identifier: Wiaver of Documentation of Informed Consent
☐	Other, Please Explain:	☒ No	☐ Yes	If Yes, insert identifier:
	**11b. RESEARCH-RELATED USE OF ANY OF THE FOLLOWING:**			
☐	Investigational Drugs/Biologics:	☒ No	☐ Yes	If Yes, insert IND#:
	FDA Approved Drug(s) for an Unapproved Use<br>If Off-Label Use, an IND is not always required. If sponsor cooperating with goal of extending use of drug an IND is required	☒ No	☐ Yes	Comments:
	FDA Approved Drug(s) for an Unapproved Subject Group<br>If Off-Label Use, an IND is not always required. If sponsor cooperating with goal of extending use of drug an IND is required	☒ No	☐ Yes	Comments:
☐	Investigational Devices:	☒ No	☐ Yes	IDE#: and Date:
	Risk Assessed by Sponsor			☐ Significant Risk (SR)<br>☐ Non-Significant Risk (NSR)
☐	Humanitarian Device Exemption:	☒ No	☐ Yes	HDE #:
	**11c. DESIGN OF STUDY:**			
☐	Placebo Controlled	☒ No	☐ Yes	Comments:
☐	Blinded	☒ No	☐ Yes	If Yes, ☐ Double Blind **or** ☐ Single Blind
☒	Randomized	☐ No	☒ Yes	Comments:
☒	Anonymous Survey or Questionnaire	☐ No	☒ Yes	Comments:
☐	Banking of Tissue / Specimen / Data	☒ No	☐ Yes	Comments:
☐	Retrospective Review of Records or Information	☒ No	☐ Yes	Comments:
☐	Registry Study	☒ No	☐ Yes	Comments:
☐	Compassionate Use – Contact IRB office for guidance	☒ No	☐ Yes	Comments:
☐	Other:	☒ No	☐ Yes	Comments:
	**11d. SAFETY MEASURES:**			

☐	Data/safety monitoring is included in the study.	☒ No	☐ Yes	If yes, details must be provided within the protocol or as an attachment.
	**Please specify the type of monitoring:**			
☐	Local data and safety monitoring plan in place	☒ No	☐ Yes	Comments:
☐	Sponsor reviews adverse events, interim findings and relevant literature	☒ No	☐ Yes	Comments:
☐	Data Safety Monitoring Board [DSMB], Data Monitoring Committee (DMC) or other similar body in place	☒ No	☐ Yes	Comments:
	**Other measures:**			
☐	Certificate of Confidentiality *(for genetic research involving identified samples)*	☒ No	☐ Yes	Comments:
☐	Other:	☒ No	☐ Yes	Comments:
	11e. USE OF SPECIMENS OR DATA: Tissue/data banking and genetic research require additional protections for subjects.			
☐	Genetic research will be done on biologic samples.	☒ No	☐ Yes	If Yes, ☐ Samples will be de-identified<br>☐ Samples will be identified
☐	Gene therapy vectors or recombinant DNA products will be used.	☒ No	☐ Yes	If Yes, EVMS Biosafety Committee Approval # on
☐	Cell lines will be developed	☒ No	☐ Yes	Comments:
☐	Cell lines from unidentified subjects will be used in this research study.	☒ No	☐ Yes	Comments:
☒	Samples/data will be used and kept for the use of **this study only.**<br>The intent is **NOT TO ESTABLISH** a "tissue/data bank.	☐ No	☒ Yes	Comments:
☐	Samples/data will be stored/banked for the use of **the investigators OR others.**<br>The intent is **TO ESTABLISH** a repository or bank.	☒ No	☐ Yes	Comments:<br>☐ Samples will be de-identified<br>☐ Samples will be identified
	If yes, provide the IRB # for protocol to govern collection and storage of samples:			IRB #:
☐	Certificate of Confidentiality *(for genetic research involving identified samples)*	☒ No	☐ Yes	Comments:
	11f. SPONSOR AND/OR GRANTING AGENCY:			
☐	Sponsor is a Federal granting agency.<br>[If Federally funded by NIH, you must submit the entire grant with this application.]	☒ No	☐ Yes	Name of Sponsor:
☐	Sponsor is a commercial company.	☒ No	☐ Yes	Name of Sponsor:
☐	Sponsor is a non-profit granting entity.	☒ No	☐ Yes	Name of Sponsor:
☐	Sponsor is academic/hospital department or personal funds.	☒ No	☐ Yes	Name of Sponsor:
	**IF YES TO ANY OF THE ABOVE, PLEASE ANSWER THE FOLLOWING QUESTIONS**<br>Who is the Principal Investigator on the award?<br>To which entity/institution is the primary award made?			
☒	Unsupported, no funding	☐ No	☒ Yes	Comments:

12.	TO THE BEST OF YOUR KNOWLEDGE, HAS THIS STUDY ALREADY BEEN APPROVED BY AN EVMS IRB UNDER

13.	VERIFICATION OF SCIENTIFIC REVIEW AND ACCEPTANCE STATEMENT:
	It is necessary for each principal investigator to verify the scientific merit of a new study before submitting the study for IRB review. Based on information submitted by the principal investigator, the appropriate department chair (or designee), certifies the conduct of the study under his/her department.
	By signing below, you confirm that you have sufficient staff and facilities to conduct this study
	By signing below, you agree to abide by the EVMS IRB Assurance which specifies compliance with OHRP Regulations for Protection of Human Research Subjects, and you agree to conduct your research: 1) according to the guidelines of this statement, 2) according to human subjects regulations outlined in the human subjects training you have completed, and 3) according to the information you supplied in this Application.
	BY SIGNING BELOW, YOU UNDERSTAND YOU MUST OBTAIN WRITTEN IRB APPROVAL BEFORE INITIATING ANY RESEARCH PROCEDURES OR ACTIVITY.

PRINCIPAL INVESTIGATOR SIGNATURE:	DATE OF SIGNATURE
[signature]	2 / 20 / 18

14.	DEPARTMENT CHAIR CERTIFICATION:		
This protocol has been reviewed by me or an appropriate designee and I agree that this study has scientific merit.			
DEPARTMENT CHAIR OR DESIGNEE OR SIGNATURE:			DATE OF SIGNATURE
Signature:	[signature]		2 / 19 / 18
Printed Name:	Ronald A Stine	Department:	Cardiology

THIS SECTION FOR IRB USE ONLY

FINAL DISPOSITION:

REVIEW CATEGORY		ACTION		CONTINUING REVIEW DEADLINE	
☐ Exempt		☐ Approved		___/___/___	
☐ Expedited		☐ Disapproved			
☐ Full (Convened) Board					
IRB SIGNATURE:				DATE:	___/___/___
SIGNED BY:	☐ IRB Chair	☐ IRB Vice Chair	☐ IRB Member		

	ANOTHER INVESTIGATOR?		
	☒ No	☐ Yes	If yes, provide: Investigator's Name: and IRB #:

13. VERIFICATION OF SCIENTIFIC REVIEW AND ACCEPTANCE STATEMENT:	
It is necessary for each principal investigator to verify the scientific merit of a new study before submitting the study for IRB review. Based on information submitted by the principal investigator, the appropriate department chair (or designee), certifies the conduct of the study under his/her department.	
By signing below, you confirm that you have sufficient staff and facilities to conduct this study	
By signing below, you agree to abide by the EVMS IRB Assurance which specifies compliance with OHRP Regulations for Protection of Human Research Subjects, and you agree to conduct your research: 1) according to the guidelines of this statement, 2) according to human subjects regulations outlined in the human subjects training you have completed, and 3) according to the information you supplied in this Application.	
BY SIGNING BELOW, YOU UNDERSTAND YOU MUST OBTAIN WRITTEN IRB APPROVAL BEFORE INITIATING ANY RESEARCH PROCEDURES OR ACTIVITY.	
PRINCIPAL INVESTIGATOR SIGNATURE:	DATE OF SIGNATURE
	____/____/____

14. DEPARTMENT CHAIR CERTIFICATION:			
**This protocol has been reviewed by me or an appropriate designee and I agree that this study has scientific merit.**			
DEPARTMENT CHAIR OR DESIGNEE OR SIGNATURE:			DATE OF SIGNATURE
**Signature:**			____/____/____
**Printed Name:**		**Department:**	

THIS SECTION FOR IRB USE ONLY

FINAL DISPOSITION:

REVIEW CATEGORY		ACTION		CONTINUING REVIEW DEADLINE
☐ Exempt		☑ Approved		2 / 1 / 19
☑ Expedited		☐ Disapproved		
☐ Full (Convened) Board				
IRB SIGNATURE:	[signature]		DATE:	5 / 11 / 18
SIGNED BY:	☐ IRB Chair	☑ IRB Vice Chair	☐ IRB Member	

IRB APPROVAL
DATE 5/11/18
EXPIRES
DATE 5/10/19
IRB # 18-04-EX-0062

Application for Approval of Research Involving Human Subjects

DO NOT EXCEED TWO (2) PAGES AND DO NOT INCLUDE EXTRA PAGES

Study Title:	A Study to Evaluate Strategies for Teaching Effective Use of Diagnostic Tests	IRB Number:
Principal Investigator:	John Brush, MD	18-04EX-0062

1. CLEARLY STATE THE PURPOSE OF THE STUDY:
The present study is designed to contrast two instructional methods – explicit instruction in likelihood ratios and pretest/posttest probabilities versus implicit instruction based on presentation of multiple cases. These will be compared to a "no intervention" control group.<br>One study group will be provided with explicit instructions on the rationale of using anchoring and adjusting for a diagnostic inquiry and how likelihood ratios have a multiplicative effect on decision-making. The other group will be provided with a series of cases with feedback about posttest probability. The intent is not to instruct students in the mathematical manipulations involved in computing Bayes' theorem. Conversely there is no assumption that they are acquiring a general skill. Rather, both instructional approaches are directed to providing learners with a "gestalt" of how a particular test will perform with a particular patient case.

2. PROVIDE A BRIEF DESCRIPTION OF DESIGN:
This is a 3-group experimental design testing two instructional interventions against a no-intervention control group. A total of 120 senior preclinical medical students will be recruited. 60 3rd year and early 4th year medical students will be recruited locally.

3. PARTICIPANT INFORMATION:
**Duration of individual subject's total involvement (provide all details – active; long-term follow-up, etc.):**<br>Surveys will be available for 72 hours after informed consent is obtained.
**How will subjects be recruited?** Medical students will be recruited via advertisement guidelines suggested and approved by this committee. Students will be approached by email.
**Inducements to participate:** $30.00
**Inclusion Criteria:** 3rd or early 4th year medical students at EVMS
**Exclusion Criteria:** 3rd year students who have not yet completed Internal Medicine Clerkship

4. BENEFITS TO SUBJECTS (DO NOT USE WORDING SUCH AS "YOU", "YOUR", ETC.):
Students in all 3 experimental groups have the potential to receive educational benefit from their participation. However, participation will not provide any benefit to academic standing or increased merit compared to students not participating. Subjects will be provided with a cash stipend of $30.00 for their time and participation in this research study.

5. RISKS TO SUBJECTS (DO NOT USE WORDING SUCH AS "YOU", "YOUR", ETC.):
There is a risk of data breach and loss of personally identifiable information (full name and email address). Students' academic standing will not be effected by participation, or choosing not to participate. Resposnes to study questions will not become part of the students' academic record.

6. MEASURES TO MINIMIZE RISKS:
Data will be anonymized and will not become part of the medical students' academic record.
