# Supplementary material for: Effect of Teaching Bayesian Methods Using Learning by Concept vs Learning by Example on Medical Students’ Ability to Estimate Probability of a Diagnosis: A Randomized Clinical Trial
Source: JAMA Netw Open. 2019 Dec 20;2(12):e1918023. doi: 10.1001/jamanetworkopen.2019.18023 (PMC7027434; doi:10.1001/jamanetworkopen.2019.18023)
Supplement: Supplement 2. — eAppendix. Content Provided to Participants and Test Questions [file ZOI190678supp2_prod.pdf]

Supplementary Online Content

Brush JE Jr, Lee M, Sherbino J, Taylor-Fishwick JC, Norman G. Effect of teaching bayesian methods using learning by concept vs learning by example on medical students' ability to estimate probability of a diagnosis: a randomized clinical trial. *JAMA Netw Open*. 2019;2(12):e1918023. doi:10.1001/jamanetworkopen.2019.18023

eAppendix. Content Provided to Participants and Test Questions

This supplementary material has been provided by the authors to give readers additional information about their work.

© 2019 Brush JE Jr et al. JAMA Network Open.

Supplemental Material

The content that was provided to study participants and the test questions are provided in the following pages:

1. Experience Condition, pages 1-60
    a. Consent
    b. Instructions
    c. Cases
2. Control Condition, pages 1-41
    a. Consent
    b. Instructions
    c. Reading material
3. Concept Condition, pages 1-13
    a. Consent
    b. Link to video and 3 cases
4. Test for Experience and Control Conditions, pages 1-39
    a. Instructions
    b. Questions
5. Test for Concept Condition, pages 1-44
    a. Instructions
    b. Questions

© 2019 Brush JE Jr et al. JAMA Network Open.

Experiential Group

1. Consent
2. Instructions
3. Cases

© 2019 Brush JE Jr et al. JAMA Network Open.

EBM Study E2

There are 83 questions in this survey

Please review the following consent form.

© 2019 Brush JE Jr et al. JAMA Network Open.

Questionnaire Consent Form

Eastern Virginia Medical School (EVMS) Institutional Review Board

Study Title: ***A Study to Evaluate Strategies for Teaching Effective Use of Diagnostic Tests (EBM)***

Name of Investigator: John Brush, MD

Sponsor: None

You are being asked to participate in a research study involving the collection of information in the form of questionnaire. The purpose of the research project is to examine the effective use of diagnostic tests in clinical reasoning among pre-clinical medical students.

The study involves an online 90-minute session. You will receive standard instructions about how to use diagnostic tests, then either a) more detailed conceptual instruction about the relation between diagnostic test characteristics and disease probability, or b) a series of systematically chosen cases showing how test characteristics can influence probability of disease or c) a control condition where you will learn more about the specific conditions used in the study. You will then be asked to interpret 20 cases by providing a diagnosis and probability before and after receiving the results of the test.

You will reimbursed $30 for your participation. There are no additional costs to you associated with taking part in this study.

A risk associated with allowing your data to be saved is the release of personal information from your study record. We will strive to protect your records so that your personal information (like name, address, social security number and phone number) will remain private. There also may be other risks that are unknown and we cannot predict.. Your academic standing will not be effected by participation, or choosing not to participate. Responses to study questions will not become part of your academic record.

Although the results of this research may not benefit you directly, they may be made available upon request. You have the potential to receive educational benefit from participation. Your participation will contribute to our understanding of this skill, and may benefit future trainees. However, participation will not provide any benefit to your academic standing or increased merit compared to students not participating.

In conducting this research study, the data from the study will be analyzed statistically to test study hypotheses, and will be retained in an anonymized form for 10 years. No individual identifiers will be present in the database. Your protected health information (PHI), which includes personal information about you only be collected.

© 2019 Brush JE Jr et al. JAMA Network Open.

Your study records may be reviewed and/or copied in order to meet state and/or federal regulations. Reviewers may include, for example, Eastern Virginia Medical School Institutional Review Board, study investigator, John Brush, MD and his research team, and the research collaborators at McMaster University.

Information learned from this research may be used in reports, presentations and publications. None of these will personally identify you.

Taking part in this study is your choice. If you decide not to take part, your choice will not affect any medical benefits to which you are entitled. You may choose to leave the study at any time by notifying the study investigator. The study investigator may decide to take you off this study if you revoke your authorization.

We will tell you about new information that may affect your health, welfare, or willingness to stay in this study.

In the event of injury resulting from this research study, Eastern Virginia Medical School (EVMS) provides no financial compensation plan or free medical care.

If you have any questions pertaining to this research you may contact John Brush, MD or Judith Taylor-Fishwick at DiagnosticReasoningStudy@evms.edu, 757-446-8475. If you believe you have suffered an injury as a result of your participation in this study, you should contact the principal investigator, John Brush, MD at 757-446-8475. You may also contact Betsy Conner, director, EVMS Human Subjects Protection Program and IRB office at (757) 446-5854. If you have any questions pertaining to your rights as a research subject, you may contact a member of the Institutional Review Board through the Institutional Review Board office at (757) 446-8423.

You are being asked to participate in the above research study, which is being conducted at Eastern Virginia Medical School (EVMS), where you are an employee or student. The research study has been described to you, in writing, on this electronic consent form. You have also had the opportunity to ask the investigators conducting this study any questions that you may have regarding participation in this study.

The purpose of this consent form is to inform you that you have the right to choose not to participate in this research study. If you choose not to participate, or to withdraw at any time, it will not affect your standing as an employee or student.

If you are an employee, your participation will not place you in good favor with the investigator, your supervisor, or EVMS (e.g., increase in salary, promotion, extra vacation, or the like). Not participating will not adversely affect your employment with EVMS, in particular the position that you currently hold. If you are a student, your participation will not place you in good favor with the investigator or other faculty (e.g., receiving better grades, recommendations,

© 2019 Brush JE Jr et al. JAMA Network Open.

employment). Also, not participating in this study will not adversely affect your relationship with the investigator or other faculty.

By clicking "Agree", you are consenting to this study. You are able to print out a copy of this consent form for your records.

Note: Do NOT click on the "BACK" button in your browser while completing this study. *

Please choose **all** that apply:

☐ Agree

If you do NOT agree, you can exit this study by closing your browser or clicking on the "exit and clear survey" button below.

© 2019 Brush JE Jr et al. JAMA Network Open.

Instructions

In this session, you will be presented with a total of 27 cases.

For each case, after clinical information is presented, you will be asked for a probability (1-100) of the diagnosis. Then, a test result will be given and you will be asked for a post-test probability of the diagnosis.

YOU MUST COMPLETE ALL OF THE CASES IN ONE SITTING. If you don't have approximately 30 minutes at the moment, please click on the "Exit and clear survey" button at the bottom of this page and come back to it later using the same link.

Thanks.

© 2019 Brush JE Jr et al. JAMA Network Open.

Case 1 of 27

An 80 year-old woman is presents to the ED with acute shortness of breath. She has had several myocardial infarctions in the past, has a history of chronic systolic heart failure and presents acutely short of breath. She lives alone and usually her daughter brings her meals to her. Over the past week, however, her daughter has been out of town and the family arranged for a neighbor to prepare the patient's meals. The neighbor supplied canned vegetables and soups and the patient noticed that the food was more salty than she was used to. She has been quite thirsty and increased her fluid intake over the past week. The day prior to admission she became more short of breath and she was unable to sleep last night due to difficulty breathing. She noticed that her ankles and hands have become swollen recently. She denies chest pain or palpitations.

PMH is remarkable for a history of coronary artery disease, hypertension, and chronic systolic heart failure. Her medications are carvedilol, lisinipril, and furosemide, which she has been taking regularly.

The patient is widowed. She formerly worked as a hairdresser. She does not smoke or drink alcohol.

On exam, HR=105, BP=100/60, RR=32. She is elderly, frail, and acutely short of breath. HEENT reveals bluish lips. Neck exam reveals jugular venous distension. Lungs reveal wet crackles throughout. She has a tachycardic heart rhythm and an S3 gallop. The abdomen is slightly distended and benign. There is 1+ bilateral pitting edema.

On a scale of 0 (very IMprobable) to 100 (very PROBABLE), what is the probability that this patient has congestive heart failure? *

Each answer must be between 0 and 100
Only an integer value may be entered in this field.

Please write your answer here:

© 2019 Brush JE Jr et al. JAMA Network Open.

Your initial probability answer was:
{INSERTANS:137236X3510X27741}

A chest x-ray is performed. The radiologist interprets the x-ray as positive for pulmonary venous congestion.

Now what is the probability that the patient has congestive heart failure? *

Each answer must be between 0 and 100
Only an integer value may be entered in this field.

Please write your answer here:

Experts indicate that the correct answer for this case is: *congestive heart failure*

© 2019 Brush JE Jr et al. JAMA Network Open.

Case 2 of 27

A 25-year-old woman presents to the ED with shortness of breath. One month ago, she delivered her second child with an uncomplicated normal vaginal delivery. She had mild nausea early in her pregnancy and some fatigue and shortness late in her pregnancy, but those symptoms resolved after delivery. In the past 2 weeks, however, she has developed progressive shortness of breath. Now she is short of breath just walking from the bedroom to the bathroom. She has notices that she sleeps better with her head elevated on 2 to 3 pillows. Her ankles are moderately swollen. The left ankle is slightly more swollen and slightly erythematous.

Past medical history is remarkable for a history of deep venous thrombosis after her first pregnancy. She took warfarin for a year and this problem resolved. She is currently taking prenatal vitamins.

She is married and lives with her husband and 3 year-old son. She is planning to return to work as a secretary in another month. She does not smoke and drinks socially.

On exam she is afebrile, HR=110, BP=100/60, RR=28. She is mildly obese and appears mildly short of breath. Her neck is obese and the jugular veins are not visualized. Lungs reveal scattered rales. Cardiac exam reveals a slightly rapid heart rate, soft systolic murmur and there is 2+ bilateral pitting ankle edema.

On a scale of 0 (very IMprobable) to 100 (very PROBABLE), what is the probability that this patient has congestive heart failure? *

Each answer must be between 0 and 100
Only an integer value may be entered in this field.

Please write your answer here:

© 2019 Brush JE Jr et al. JAMA Network Open.

Your initial probability answer was:
{INSERTANS:137236X3484X27663}

A chest x-ray is performed. The radiologist interprets the x-ray as negative for pulmonary venous congestion.

Now what is the probability that the patient has congestive heart failure? *

Each answer must be between 0 and 100
Only an integer value may be entered in this field.

Please write your answer here:

Experts disagree what the diagnosis is at this point; *it could either be CHF due to post-partum cardiomyopathy or a pulmonary embolism (PE).*

© 2019 Brush JE Jr et al. JAMA Network Open.

Case 3 of 27

A 50-year-old woman present to the ED with a 2 week history of shortness of breath. She is now short of breath even walking from one room to the next. She sleeps on 2 pillows at night and has been doing so for most of her life. Over the past month or so, she has noted that she has had mild diarrhea and her stools are now dark, almost black. She complains of mild vague abdominal pain, but no nausea or vomiting or chest pain.

PMH is remarkable for a history of arthritis for which she uses NSAIDs prn.

On exam, she is afebrile, HR=115 bpm, BP=110/80, RR=28. She appears pale and mildly short of breath sitting in the hospital bed. Lungs are clear to auscultation and her cardiac exam is remarkable for a tachycardic rhythm and a soft systolic murmur. The abdominal exam reveals mild generalized tenderness and no masses or rebound tenderness. Rectal exam reveals dark stool that is Hemoccult positive. There is no peripheral edema.

On a scale of 0 (very IMprobable) to 100 (very PROBABLE), what is the probability that this patient has congestive heart failure? *

Each answer must be between 0 and 100
Only an integer value may be entered in this field.

Please write your answer here:

Your initial probability answer was:
{INSERTANS:137236X3485X27666}

A chest x-ray is performed. The radiologist interprets the x-ray as negative for pulmonary venous congestion.

Now what is the probability that the patient has congestive heart failure? *

Each answer must be between 0 and 100
Only an integer value may be entered in this field.

Please write your answer here:

© 2019 Brush JE Jr et al. JAMA Network Open.

Experts indicate that the correct answer for this case is: *shortness of breath due to severe anemia.*

© 2019 Brush JE Jr et al. JAMA Network Open.

Case 4 of 27

A 68-year-old man presents to the ED with a 3-day history of progressive shortness of breath. He has chronic shortness of breath and a chronic cough productive of white sputum in the morning. He uses chronic inhalers several times daily and as needed for wheezing. His activity has been limited for about 2 years, but the shortness of breath worsened over the past 3 days, associated with an increase in his cough and now with the production of green sputum. He sleeps flat and denies ankle edema. He continues to smoke ½ pack of cigarettes daily, formerly smoked 2 packs daily, and has been smoking off and on since age 16.

PMH is remarkable for COPD treated with inhaled bronchodilators and hypertension treated with lisinopril.

On exam, he is afebrile, HR=110 bpm, BP=140/90, RR=28. He is using his accessory muscles to breath and is in moderate respiratory distress. HEENT exam is unremarkable. Lung exam reveals distant breath sounds and scattered wheezing. Heart exam reveals a tachycardic, regular rhythm and a soft systolic murmur. Extremities shows slight cyanosis and clubbing and no peripheral edema.

On a scale of 0 (very IMprobable) to 100 (very PROBABLE), what is the probability that this patient has congestive heart failure? *

Each answer must be between 0 and 100
Only an integer value may be entered in this field.

Please write your answer here:

Your initial probability answer was: {INSERTANS:137236X3486X27669}

A chest x-ray is performed. The radiologist interprets the x-ray as negative for pulmonary venous congestion.

Now what is the probability that the patient has congestive heart failure? *

Each answer must be between 0 and 100
Only an integer value may be entered in this field.

Please write your answer here:

© 2019 Brush JE Jr et al. JAMA Network Open.

Experts indicate that the correct answer for this case is: *acute exacerbation of chronic obstructive pulmonary disease (COPD)*

© 2019 Brush JE Jr et al. JAMA Network Open.

Case 5 of 27

A 75-year-old woman presents to the ED with progressive shortness of breath. About 10 years ago, she had a “massive heart attack.” She was hospitalized in another city and was treated with emergency stenting of “the artery on the front of her heart,” according to the patient’s memory. She was in the hospital about 3 weeks and subsequently moved to her current home to be closer to her daughter, who now lives nearby. Over the past 3 weeks, she has developed progressive shortness of breath. She is able to only do light housework and can no longer run the vacuum cleaner or change the beds. She now sleeps on 3 pillows at night and sometimes has to sit up on the side of the bed at night to catch her breath. She denies chest pain. She indicates that her shoes won’t fit because of swelling of her legs and that she has seemed to gain weight.

PMH is remarkable for a history of MI. She takes lisinopril, carvedilol.

On exam, her HR=110, BP=160/90, RR=28. She is in moderate distress due to shortness of breath. Lungs reveal bibasilar rales. Cardiac exam reveals a tachycardic rhythm and a possible S3 gallop. The abdomen is benign.

On a scale of 0 (very IMprobable) to 100 (very PROBABLE), what is the probability that this patient has congestive heart failure? *

Each answer must be between 0 and 100
Only an integer value may be entered in this field.

Please write your answer here:

Your initial probability answer was: {INSERTANS:137236X3487X27672}

A chest x-ray is performed. The radiologist interprets the x-ray as positive for pulmonary venous congestion.

Now what is the probability that the patient has congestive heart failure? *

Each answer must be between 0 and 100
Only an integer value may be entered in this field.

Please write your answer here:

© 2019 Brush JE Jr et al. JAMA Network Open.

Experts indicate that the correct answer for this case is: *congestive heart failure*

© 2019 Brush JE Jr et al. JAMA Network Open.

Case 6 of 27

A 48-year-old man presents to the ED with progressive shortness of breath. He has longstanding hypertension that has been difficult to control. He has been on multiple medications for blood pressure control and he recently ran out of his clonidine. He developed shortness of breath about a month ago, which is now severe, occurring with minimal activity. He usually sleeps on 2 pillows, but over the past week he has been more comfortable sleeping in a recliner. He has some ankle edema and he thinks his weight may be up several pounds.

PMH is remarkable for hypertension treated with amlodipine, a thiazide diuretic, metoprolol twice daily and clonidine twice daily, which was recently stopped.

On exam, he is mildly obese in moderate distress due to shortness of breath. He is afebrile, HR=100, BP=200/110, RR=28. He has jugular venous distension noted sitting up on the ED stretcher. Cardiac exam reveals a tachycardic rhythm and a soft mid-peaking systolic murmur. The abdomen is benign. Lungs reveal bibasilar rales ½ way up from the bases. There is 2+ bilateral ankle edema.

On a scale of 0 (very IMprobable) to 100 (very PROBABLE), what is the probability that this patient has congestive heart failure? *

Each answer must be between 0 and 100
Only an integer value may be entered in this field.

Please write your answer here:

Your initial probability answer was: {INSERTANS:137236X3488X27675}

A chest x-ray is performed. The radiologist interprets the x-ray as positive for pulmonary venous congestion.

Now what is the probability that the patient has congestive heart failure? *

Each answer must be between 0 and 100
Only an integer value may be entered in this field.

Please write your answer here:

© 2019 Brush JE Jr et al. JAMA Network Open.

Experts indicate that the correct answer for this case is: *congestive heart failure*

© 2019 Brush JE Jr et al. JAMA Network Open.

Case 7 of 27

A 68-year-old man presents to the ED with progressive shortness of breath. He had a myocardial infarction 10 years ago and underwent coronary artery bypass grafts (CABG) x 4 at that time. He continues to smoke, sees a pulmonary physician and takes inhalers and low dose steroids for chronic obstructive pulmonary disease (COPD). Over the past month or so, his shortness of breath has worsened. He is short of breath doing routine things around the house and no longer is able to walk his dog around the block. He has always slept on 2 pillows but lately falls asleep in his recliner while watching TV. He denies chest pain but has noticed that his shoes are tighter lately due to edema and his weight is up slightly.

PMH is remarkable for CABG, COPD, hypertension, and hyperlipidemia. He has been compliant with his meds, which include amlodipine, atorvastatin, aspirin, prednisone and inhaled bronchodilators.

On exam, his HR=110, BP=140/70, RR=28. He is mildly obese and mildly short of breath at rest. His lung exam reveals diffuse rhonchi and scattered coarse rales. Cardiac exam reveals a slightly fast heart rate and a soft systolic murmur. There is no edema.

On a scale of 0 (very IMprobable) to 100 (very PROBABLE), what is the probability that this patient has congestive heart failure? *

Each answer must be between 0 and 100
Only an integer value may be entered in this field.

Please write your answer here:

Your initial probability answer was: {INSERTANS:137236X3489X27678}

A chest x-ray is performed. The radiologist interprets the x-ray as positive for pulmonary venous congestion.

Now what is the probability that the patient has congestive heart failure? *

Each answer must be between 0 and 100
Only an integer value may be entered in this field.

Please write your answer here:

© 2019 Brush JE Jr et al. JAMA Network Open.

Experts disagree what the diagnosis is at this point; *it could either be congestive heart failure (CHF) or chronic obstructive pulmonary disease (COPD).*

© 2019 Brush JE Jr et al. JAMA Network Open.

Case 8 of 27

A 60-year-old African American man presents to the ED with progressive shortness of breath. He has long-standing hypertension that has been hard to control. He has been on multiple medications for his hypertension in the past but has had problems paying for his medications and stopped taking his BP meds about 6 months ago. Over the past month, he had developed chest pain with exertion. He works for a moving company lifting furniture and has had to stop working over the past 2 weeks. He is now short of breath and when he climbs the steps to his bedroom, he has to stop half way to catch his breath. He has had vague headaches recently. He now sleeps on 2-3 pillows.

PMH-he has hypertension and records show he was on a thiazide diuretic and beta blocking agent in the past, but he is currently taking no medications.

On exam, his HR=80, BP=190/100, RR=20. In general, he is obese and mildly short of breath. He has mild jugular venous distension, clear lungs on exam. His cardiac exam reveals a normal S1 and S2 and an S4 gallop. Exam is otherwise unremarkable.

On a scale of 0 (very IMprobable) to 100 (very PROBABLE), what is the probability that this patient has congestive heart failure? *

Each answer must be between 0 and 100
Only an integer value may be entered in this field.

Please write your answer here:

Your initial probability answer was: {INSERTANS:137236X3490X27681}

A chest x-ray is performed. The radiologist interprets the x-ray as positive for pulmonary venous congestion.

Now what is the probability that the patient has congestive heart failure? *

Each answer must be between 0 and 100
Only an integer value may be entered in this field.

Please write your answer here:

© 2019 Brush JE Jr et al. JAMA Network Open.

Experts disagree what the diagnosis is at this point; *it could either be CHF or uncontrolled hypertension.*

© 2019 Brush JE Jr et al. JAMA Network Open.

Case 9 of 27

A 30-year-old man presents to the ED with worsening shortness of breath over the past week or so. He has a history of depression and chronic anxiety. He takes and anti-depression medication and Xanax prn. Two weeks ago, he had an argument with his boss. He is concerned about whether he may be laid off. He is having trouble sleeping at night and sometimes sleeps in his recliner while watching TV. He denies fever, cough, chest pain, ankle edema.

PMH is remarkable for a history of depression, treated with a daily medication. He cannot remember the name of the drug.

On exam, he is somewhat anxious appearing. He is afebrile, HR=105, BP=150/80, RR=28. His lungs are clear to auscultation, heart exam reveals a slightly tachycardic rhythm and a soft systolic murmur along the left sternal border. The abdomen is benign and there is no peripheral edema.

On a scale of 0 (very IMprobable) to 100 (very PROBABLE), what is the probability that this patient has congestive heart failure? *

Each answer must be between 0 and 100
Only an integer value may be entered in this field.

Please write your answer here:

Your initial probability answer was: {INSERTANS:137236X3491X27684}

A chest x-ray is performed. The radiologist interprets the x-ray as negative for pulmonary venous congestion.

Now what is the probability that the patient has congestive heart failure? *

Each answer must be between 0 and 100
Only an integer value may be entered in this field.

Please write your answer here:

© 2019 Brush JE Jr et al. JAMA Network Open.

Experts indicate that the correct answer for this case is: *anxiety*

© 2019 Brush JE Jr et al. JAMA Network Open.

Case 10 of 27

A 29-year-old woman presents complaining of chest heaviness. For the past three days she has complained of sinus congestion, a sore throat and a dry non-productive cough. She is worried that she might have caught something from her roommate, who has also been sick for the past few days. However, her inability to catch her breath is the reason that she is attending the emergency department. She denies any pleuritic chest pain or calf swelling. She was able to exercise yesterday without feeling short of breath. However, she kept coughing, so she gave up when people at the gym kept giving her a dirty look.

The patient is well without any other medical problems. She is a non-smoker. Her only medication is ibuprofen, which she has been taking for the last 3 days to address her sore throat and because “her body feels achy and run down.”

On examination, her temperature is 37.3C; pulse 76 / minute; blood pressure 110/76mmHg; and respiratory rate 18 / minute. Her heart rate is regular. She has normal pulses. There are no extra cardiac sounds or murmurs. Her chest exam is normal with an occasional transmitted upper respiratory sound. She has an inflamed oropharynx with without evidence of purulence. Her calves are symmetric, non-tender and not swollen.

On a scale of 0 (very IMprobable) to 100 (very PROBABLE), what is the probability that this patient has a pulmonary embolism? *

Each answer must be between 0 and 100
Only an integer value may be entered in this field.

Please write your answer here:

Your initial probability answer was: {INSERTANS:137236X3492X27687}

A quantitative D-dimer is measured. The value is less than 500, which is reported as negative.

Now what is the probability that the patient has a pulmonary embolism? *

Each answer must be between 0 and 100
Only an integer value may be entered in this field.

Please write your answer here:

© 2019 Brush JE Jr et al. JAMA Network Open.

Experts indicate that the correct answer for this case is: *an upper respiratory infection (URI)*

© 2019 Brush JE Jr et al. JAMA Network Open.

Case 11 of 27

A 66-year-old female presents with a warm, swollen right leg. There is no history of trauma. She indicates that the leg has been sore for close to one week. At first, she thought it was a flare of her lower extremity venous insufficiency; she missed a dose of her diuretic this week and has been non-compliant with wearing her compression stockings. She is seeking medical attention as she has developed chest pain today and feels lightheaded. Every time she stands up suddenly she feels as if she is going to faint. After catching her breath, she is able to walk normally. She normally participates in daily exercises but is unable to today because she feels unwell.

Her past medical history is significant for hypertension and venous insufficiency. She takes a loop diuretic daily as well as a baby aspirin.

On examination, her temperature is 37.4, pulse 118, BP 100/76, and RR 20. Her chest is clear to auscultation. Her cardiac exam reveals normal heart sounds and symmetric peripheral pulses. Her left leg is diffusely swollen and warm in comparison to the contralateral leg. There is no erythema of the skin. The inner thigh is particularly tender to palpation.

On a scale of 0 (very IMprobable) to 100 (very PROBABLE), what is the probability that this patient has a pulmonary embolism? *

Each answer must be between 0 and 100
Only an integer value may be entered in this field.

Please write your answer here:

[ ]

Your initial probability answer was:
{INSERTANS:137236X3493X27690}

A quantitative D-dimer is measured. The value is greater than 500, which is reported as positive.

Now what is the probability that the patient has a pulmonary embolism? *

Each answer must be between 0 and 100
Only an integer value may be entered in this field.

Please write your answer here:

[ ]

© 2019 Brush JE Jr et al. JAMA Network Open.

Experts indicate that the correct answer for this case is: *a pulmonary embolism*

© 2019 Brush JE Jr et al. JAMA Network Open.

Case 12 of 27

A 42-year-old man presents with pain in the right posterior upper chest. The pain has been constant for the last 2 days. It is described as a burning sensation that becomes more intense every time he moves his shoulder. He has no cough or shortness of breath. He experienced a similar problem 4 months ago, which he attributed to a starting a new weight lifting program at the gym. However, he doesn't recall any specific injury this time. Because the pain has not resolved he is seeking medical attention. His wife indicates that the biggest problem is that he is not sleeping because of the discomfort. Every time he lies on his right side the burning sensation intensifies.

His past medical history is only significant for erectile dysfunction. He uses no medication regularly, although he volunteers that he uses recreational marijuana most weekends.

On examination his temperature is 36; pulse 89 / minute; blood pressure 140/96mmHg; and respiratory rate 16 / minute. His cardiac exam is within normal limits without extra heart sounds or murmurs. His chest exam reveals equal air entry bilaterally. When palpating the area that he describes as sore, the rhomboids are noted to be particularly tender. He has no swelling of his calves.

On a scale of 0 (very IMprobable) to 100 (very PROBABLE), what is the probability that this patient has a pulmonary embolism? *

Each answer must be between 0 and 100
Only an integer value may be entered in this field.

Please write your answer here:

Your initial probability answer was: {INSERTANS:137236X3494X27693}

A quantitative D-dimer is measured. The value is less than 500, which is reported as negative.

Now what is the probability that the patient has a pulmonary embolism? *

Each answer must be between 0 and 100
Only an integer value may be entered in this field.

Please write your answer here:

© 2019 Brush JE Jr et al. JAMA Network Open.

Experts indicate that the correct answer for this case is:
musculoskeletal (MSK) pain

© 2019 Brush JE Jr et al. JAMA Network Open.

Case 13 of 27

A 33-year-old man presents complaining of retrosternal chest pain. The history is difficult to obtain as the patient is hyperventilating, rapidly talking and difficult to direct during the interview. He describes sudden onset of anterior chest pain – “like a crushing weight” – that happened without warning or trigger. He feels he can’t catch his breath. His mother experienced something similar 5 years ago and was diagnosed with a massive saddle pulmonary embolism as a result of a Factor V Leiden deficiency. The patient is very anxious and feels that “something bad is going to happen.”

His past medical history is significant for an anxiety disorder with provocative panic attacks. He takes paroxetine regularly to treat his anxiety. During significant episodes we will use lorazepam to decrease symptoms. He has already taken a dose of lorazepam today to help relieve the anxiety he is experiencing. He has never been tested to see if he has any genetic coagulopathies.

On examination, his temperature is 37.2, pulse 105, BP 110/66, and RR 24. His chest is clear to auscultation. His cardiac exam reveals no abnormalities, including murmurs, extra heart sounds or rubs. His right calf is tender to palpation; however, the patient indicates that he strained his leg muscles at the gym doing a new exercise.

On a scale of 0 (very IMprobable) to 100 (very PROBABLE), what is the probability that this patient has a pulmonary embolism? *

Each answer must be between 0 and 100
Only an integer value may be entered in this field.

Please write your answer here:

© 2019 Brush JE Jr et al. JAMA Network Open.

Your initial probability answer was:
{INSERTANS:137236X3495X27696}

A quantitative D-dimer is measured. The value is less than 500, which is reported as negative.

Now what is the probability that the patient has a pulmonary embolism? *

Each answer must be between 0 and 100
Only an integer value may be entered in this field.

Please write your answer here:

Experts disagree what the diagnosis is at this point; *it could either be a pulmonary embolism or anxiety.*

© 2019 Brush JE Jr et al. JAMA Network Open.

Case 14 of 27

An 86-year-old woman presents from home post syncopal event. She describes increasing breathlessness for the past week. She also complains of posterior chest pain that she describes as constant and sharp. The syncopal event happened as she was walking from the kitchen into her living room. She does not report any injury as a result of the fall. But she does remark that it feels as if her heart is still racing and that she can't breathe. You note that while she is speaking full sentences she does appear breathless.

The patient's past medical history is remarkable for locally metastatic colon cancer. This has only recently been diagnosed. She is to begin adjuvant chemotherapy next week to reduce tumour burden prior surgical resection. She has no cardiac history and is otherwise healthy.

On examination, her temperature is 37.2, pulse 125, BP 90/56, and RR 28. Her chest is clear to auscultation. Her cardiac exam reveals normal bilateral pulses and blood pressure. She has no murmurs or extra heart sounds, although the heart rate is noted to be elevated and regular. There is no evidence of head or extremity injury on physical exam as a result of the fall.

On a scale of 0 (very IMprobable) to 100 (very PROBABLE), what is the probability that this patient has a pulmonary embolism? *

Each answer must be between 0 and 100
Only an integer value may be entered in this field.

Please write your answer here:

**Your initial probability answer was:
{INSERTANS:137236X3496X27699}**

A quantitative D-dimer is measured. The value is greater than 500, which is reported as positive.

Now what is the probability that the patient has a pulmonary embolism? *

Each answer must be between 0 and 100
Only an integer value may be entered in this field.

Please write your answer here:

© 2019 Brush JE Jr et al. JAMA Network Open.

Experts indicate that the correct answer for this case is: *a pulmonary embolism*

© 2019 Brush JE Jr et al. JAMA Network Open.

Case 15 of 27

A 67-year-old man presents with retrosternal chest pain that he describes as burning. The pain began several hours ago. The patient indicates that the burning sensation is typical of his typical reflux. However, the intensity of the symptoms and the associated sense of “it takes my breath away” prompted medical attention. The patient tried his usual concoction of over the counter antacids without any success. When he googled his symptoms, he was concerned that he might have a blood clot in his lungs. “It burns so much that I have to take tiny breaths.”

The patient’s past medical history includes type two diabetes, benign prostate hypertrophy and gastroesophageal reflux. He takes ramipril, tamsulosin, ASA and omeprazole daily. The patient also mentions that he recently had a flare of what he self-diagnosed as gout. And has been taking daily naproxen for the past 10 days. His sore toe has responded and he has no further pain.

On examination, her temperature is 37.5C; pulse 64 / minute; blood pressure 130/92mmHg; and respiratory rate 21 / minute. His cardiac exam reveals a normal S1 and S2. Auscultation of his lungs reveals no adventitious sounds. Palpation of the epigastrium causes some non-specific subjective discomfort that is difficult to reproduce.

On a scale of 0 (very IMprobable) to 100 (very PROBABLE), what is the probability that this patient has a pulmonary embolism? *

Each answer must be between 0 and 100
Only an integer value may be entered in this field.

Please write your answer here:

Your initial probability answer was:
{INSERTANS:137236X3497X27702}

A quantitative D-dimer is measured. The value is less than 500, which is reported as negative.

Now what is the probability that the patient has a pulmonary embolism? *

Each answer must be between 0 and 100
Only an integer value may be entered in this field.

Please write your answer here:

© 2019 Brush JE Jr et al. JAMA Network Open.

Experts indicate that the correct answer for this case is:
gastroesophageal reflux disease (GERD)

© 2019 Brush JE Jr et al. JAMA Network Open.

Case 16 of 27

A 54-year-old man presents with shortness of breath and chest pain. Neither the pain nor difficulty breathing are particularly debilitating. He is a busy entrepreneur and has attempted to work through his symptoms for the past 3 days without success. As the symptoms have intensified, he has sought medical attention. He thought that perhaps he was coming down with the “flu” but he has no other symptoms, including fever, myalgia, sore throat or cough. He clears his throat / coughs several times during the assessment, but a cough is not a predominant feature, rather it is a sense of breathlessness.

His past medical history is remarkable for a previous left leg deep vein thrombosis. The etiology of his previous DVT was never determined. After a 3-month period of anticoagulation, he stopped his medication on his doctor’s advice. He has no other cardiac or respiratory conditions.

On examination, his temperature is 37.7, pulse 115, BP 142/86, and RR 24. His chest is clear to auscultation. His cardiac exam is unremarkable. His left leg is swollen. The patient indicates that this discrepancy in leg sizes is a result of his previous DVT. However, on palpation of his deep venous system of his upper inner thigh he notes that it is quite tender, which is a new finding.

On a scale of 0 (very IMprobable) to 100 (very PROBABLE), what is the probability that this patient has a pulmonary embolism? *

Each answer must be between 0 and 100
Only an integer value may be entered in this field.

Please write your answer here:

© 2019 Brush JE Jr et al. JAMA Network Open.

Your initial probability answer was:
{INSERTANS:137236X3498X27705}

A quantitative D-dimer is measured. The value is greater than 500, which is reported as positive.

Now what is the probability that the patient has a pulmonary embolism? *

Each answer must be between 0 and 100
Only an integer value may be entered in this field.

Please write your answer here:

Experts indicate that the correct answer for this case is: *a pulmonary embolism*

© 2019 Brush JE Jr et al. JAMA Network Open.

Case 17 of 27

A 54-year old woman presents to the emergency department after a sudden attack of shortness of breath. This dyspnea was accompanied by retrosternal chest pain, which was worse on deep breathing. She has otherwise been feeling well this week other than a cough productive of clear or yellow sputum for several days and had three episodes in which she coughed up small amounts of blood. The patient complained of nausea and vomited a small amount of what appeared to be remnants of her dinner when driving to the hospital. She travels frequently for work, and recently returned from a conference that required a number of connecting flights to get home. She complains of feeling exhausted.

Her past medical history is remarkable for a renal transplant. She is on triple immune suppression (prednisone, tacrolimus and cyclosporine). She has had pneumonia in the past as a complication of her drug therapy. She smokes occasionally when in social settings and drinks 1 glass of wine daily. She does not use illicit drugs.

On examination, her temperature is 38.2, pulse 106, BP 110/96, and RR 22. Her oropharynx has no erythema or exudate. Her chest is resonant to percussion bilaterally with faint crackles auscultated at her left lung base. Her heart demonstrates regular rate and rhythm, with a 2/6 holosystolic murmur heard loudest at the apex without radiation.

On a scale of 0 (very IMprobable) to 100 (very PROBABLE), what is the probability that this patient has a pulmonary embolism? *

Each answer must be between 0 and 100
Only an integer value may be entered in this field.

Please write your answer here:

© 2019 Brush JE Jr et al. JAMA Network Open.

Your initial probability answer was:
{INSERTANS:137236X3499X27708}

A quantitative D-dimer is measured. The value is greater than 500, which is reported as positive.

Now what is the probability that the patient has a pulmonary embolism? *

Each answer must be between 0 and 100
Only an integer value may be entered in this field.

Please write your answer here:

Experts disagree what the diagnosis is at this point; *it could either be a pulmonary embolism or pneumonia.*

© 2019 Brush JE Jr et al. JAMA Network Open.

Case 18 of 27

A 76-year-old female is brought to the emergency department by her husband for 3 days of chest pain with a dry cough, as well as two episodes of coughing up clear sputum streaked with blood. The pain is consistent and sharp. When she takes a breath, the pain intensifies. She has had generalized muscle aches during this same time period. She has felt “feverish” though has not measured her temperature. Her appetite and energy have diminished over the past 2 days. She has been using more of her three inhalers (anticholinergic, bronchodilator and inhaled corticosteroid) to no avail. Two months ago, she was seen in the emergency department for an acute exacerbation of her chronic obstructive pulmonary disease (COPD).

Her past medical history is remarkable for previous pulmonary embolism andCOPD. She continues to smoke. She is unable to describe her medications and indicates “you must have it on file” although she can describe the colours of her puffers. She is not currently on nay anticoagulants.

On examination, her temperature is 37.8, pulse 118, BP 170/96, and RR 24. Her cardiac exam reveals a regular rate and rhythm with no murmurs, gallops or rubs. On auscultation of her chest, she has crackles at both bases with a prolonged expiratory phase. Her extremities have no swelling. On general inspection she is very thin and cachectic.

On a scale of 0 (very IMprobable) to 100 (very PROBABLE), what is the probability that this patient has a pulmonary embolism? *

Each answer must be between 0 and 100
Only an integer value may be entered in this field.

Please write your answer here:

© 2019 Brush JE Jr et al. JAMA Network Open.

Your initial probability answer was: {INSERTANS:137236X3500X27711}

A quantitative D-dimer is measured. The value is less than 500, which is reported as negative.

Now what is the probability that the patient has a pulmonary embolism? *

Each answer must be between 0 and 100
Only an integer value may be entered in this field.

Please write your answer here:

Experts disagree what the diagnosis is at this point; *it could either be a pulmonary embolism or COPD.*

© 2019 Brush JE Jr et al. JAMA Network Open.

Case 19 of 27

A 50-year-old woman presents to the ED with chest pain. She has a longstanding history of hypertension for which takes three medications. She also has a history of borderline diabetes. She ran out of her blood pressure medications 3 days ago and hasn't been able to get into see her primary care doctor to get a renewal for her prescriptions. Yesterday, she began having chest pain described as a pressure in the mid chest without radiation. The pain occurred off and on throughout the day. She was relatively inactive yesterday and the pain did not seem to worsen with activity. She felt mildly short of breath along with the pain. She decided to come to the ED today because the pain was persisting, and also to see if she could get a refill on her BP medications. She is complaining of 3/10 mid sub-sternal chest tightness.

PMH is remarkable for a 5-year history of hypertension and borderline diabetes controlled with diet. She normally takes dyazide, metoprolol, and amlodipine for hypertension.

She is married and works as a waitress. She quit smoking 3 years ago and drinks occasionally.

On examination, HR=100, BP=200/110, RR=28. She is obese in mild distress due to pain. HEENT is unremarkable, Lungs are clear, Cardiac exam reveals a regular rate and rhythm and no murmurs. The abdomen is obese and benign.

On a scale of 0 (very IMprobable) to 100 (very PROBABLE), what is the probability that this patient has an acute myocardial infarction? *

Each answer must be between 0 and 100
Only an integer value may be entered in this field.

Please write your answer here:

© 2019 Brush JE Jr et al. JAMA Network Open.

Your initial probability answer was:
{INSERTANS:137236X3501X27714}

A troponin level was drawn. The value is less than 30, which is reported as normal.

Now what is the probability that this patient has an acute myocardial infarction? *

Each answer must be between 0 and 100
Only an integer value may be entered in this field.

Please write your answer here:

Experts disagree what the diagnosis is at this point; *it could either be an acute myocardial infarction or a hypertensive emergency.*

© 2019 Brush JE Jr et al. JAMA Network Open.

Case 20 of 27

A 45-year-old man presents to the ED with chest pain. He has a longstanding history of hypertension, obesity, and continues to smoke. Three months ago, he presented with unstable angina, underwent cardiac catheterization and stenting of the obtuse marginal artery with a drug eluting stent. He was discharged and instructed to take a new medicine in addition to aspirin. The medicine was expensive, however, and he stopped taking that medication about a week ago. This morning at work, he developed chest pain described as a severe mid chest tightness without radiation, associated with sweating. He drove himself to the ED. He took a sublingual nitroglycerin in route and his pain is now 2/10 in intensity.

PMH is remarkable for hypertension and coronary artery disease. Medications are metoprolol, amlodipine, atorvastatin, aspirin, and prasugrel, which he recently stopped.

He lives with his wife and son and works as a plumber. He continues to smoke 1 ppd and is trying to stop. He drinks beers on the weekends.

On examination, HR=90, BP=130/90, RR=20. He is in moderate distress due to pain. Lungs are clear, cardiac exam is unremarkable. The chest is non-tender and the abdomen is benign.

On a scale of 0 (very IMprobable) to 100 (very PROBABLE), what is the probability that this patient has an acute myocardial infarction? *

Each answer must be between 0 and 100
Only an integer value may be entered in this field.

Please write your answer here:

Your initial probability answer was:
{INSERTANS:137236X3504X27723}

A troponin level was drawn. The value is greater than 30, which is reported as abnormal.

Now what is the probability that this patient has an acute myocardial infarction? *

Each answer must be between 0 and 100
Only an integer value may be entered in this field.

Please write your answer here:

© 2019 Brush JE Jr et al. JAMA Network Open.

Experts indicate that the correct answer for this case is: *an acute myocardial infarction*

© 2019 Brush JE Jr et al. JAMA Network Open.

Case 21 of 27

A 28-year-old woman presents to the ED with a 3 day history of chest pain. She has been under considerable stress lately related to marital problems. He works as a secretary and has had problems concentrating at work. She feels anxious and is having problems sleeping at night. For 3 days, she has experienced a vague tightness in her chest that seems to be provoked by anxiety. The tightness will last up to an hour and usually resolves when she is able to calm herself down. She remains fairly active and does not experience chest discomfort with exertion. She had chest pain at work and a co-worker insisted that she have it checked out. She is currently complaining of mild tightness in the mid chest without radiation.

PMH is remarkable only for anxiety and borderline hypertension, which is untreated.

On examination, HR=100, BP=140/90, RR=20. She appears anxious in mild distress. Lungs are clear to auscultation and her cardiac exam is normal.

On a scale of 0 (very IMprobable) to 100 (very PROBABLE), what is the probability that this patient has an acute myocardial infarction? *

Each answer must be between 0 and 100
Only an integer value may be entered in this field.

Please write your answer here:

Your initial probability answer was: {INSERTANS:137236X3503X27720}

A troponin level was drawn. The value is less than 30, which is reported as normal.

Now what is the probability that this patient has an acute myocardial infarction? *

Each answer must be between 0 and 100
Only an integer value may be entered in this field.

Please write your answer here:

© 2019 Brush JE Jr et al. JAMA Network Open.

Experts indicate that the correct answer for this case is: *anxiety*

© 2019 Brush JE Jr et al. JAMA Network Open.

Case 22 of 27

A 60-year-old man presents to the ED with chest pain. Over the past 3 weeks, he has had chest pain described as a sharp pain in his mid-chest without radiation. The pain will occur several times per day and usually occurs with exertion. He works as a letter carrier and has noticed that the pain is worse while he is walking during the day. It lasts about ½ hour and resolves spontaneously. He also notices that is improves after he eats a meal. The pain is associated with belching. He denies shortness of breath, nausea, or sweating. The pain got worse today, belching worsened, and he decided to have it checked out. He is currently complaining of 4/10 chest pain in the mid chest without radiation.

PMH is remarkable for type 2 diabetes mellitus. He takes Glucotrol and simvastatin.

On examination, HR=90, BP=130/80, RR=20. He appears comfortable. Lungs are clear and cardiac exam is unremarkable. The abdomen is obese with mild generalized tenderness.

On a scale of 0 (very IMprobable) to 100 (very PROBABLE), what is the probability that this patient has an acute myocardial infarction? *

Each answer must be between 0 and 100
Only an integer value may be entered in this field.

Please write your answer here:

Your initial probability answer was: {INSERTANS:137236X3502X27717}

A troponin level was drawn. The value is greater than 30, which is reported as abnormal.

Now what is the probability that this patient has an acute myocardial infarction? *

Each answer must be between 0 and 100
Only an integer value may be entered in this field.

Please write your answer here:

© 2019 Brush JE Jr et al. JAMA Network Open.

Experts disagree what the diagnosis is at this point; *it could either be an acute myocardial infarction or GERD.*

© 2019 Brush JE Jr et al. JAMA Network Open.

Case 23 of 27

A 50-year-old man presents to the ED with chest pain. He is fairly active, working as a house painter. Over the weekend, he was working at home, chopping and stacking firewood. When he was lifting a heavy log, he experienced a sharp pain in his mid-chest without radiation. There were no associated symptoms. He tried Tylenol with minimal relief. His father died at age 55 of a heart attack and his wife was very concerned about her husband's chest pain. She drove him to the ED to have this checked out. He is currently complaining of 5/10 chest pain.

Past medical history is remarkable for a history of type 2 diabetes mellitus, treated with diet. He takes no medications.

On examination, HR=80, BP=130/80, RR=20. He is obese and in mild distress due to pain. Lungs are clear to auscultation. Cardiac exam is unremarkable. Palpation of the chest reveals tenderness in the mid-chest area, which he thinks reproduces the paint that he has been experiencing over the past several days.

On a scale of 0 (very IMprobable) to 100 (very PROBABLE), what is the probability that this patient has an acute myocardial infarction? *

Each answer must be between 0 and 100
Only an integer value may be entered in this field.

Please write your answer here:

Your initial probability answer was: {INSERTANS:137236X3505X27726}

A troponin level was drawn. The value is less than 30, which is reported as normal.

Now what is the probability that this patient has an acute myocardial infarction? *

Each answer must be between 0 and 100
Only an integer value may be entered in this field.

Please write your answer here:

© 2019 Brush JE Jr et al. JAMA Network Open.

Experts indicate that the correct answer for this case is:
musculoskeletal chest pain

© 2019 Brush JE Jr et al. JAMA Network Open.

Case 24 of 27

A 50-year-old man presents to the ED with chest pain. He has no prior documented cardiac history. This morning, while at work as an automobile mechanic, he developed the sudden onset of chest pain described as a moderately severe dull aching pain in the mid chest associated with aching in his jaw and with diaphoresis. His boss called 911 and he was brought to the ED. He continues to complain of 9/10 chest pressure.

PMH is unremarkable. He hasn't seen a doctor in years. He smokes 1 ppd and drinks about 2 beers every day.

On examination, HR=110, BP=130/90, RR=28. He is in moderate distress due to obvious pain. Lungs are clear and cardiac exam is unremarkable. The chest and abdomen are non-tender and pulses are full and equal throughout.

On a scale of 0 (very IMprobable) to 100 (very PROBABLE), what is the probability that this patient has an acute myocardial infarction? *

Each answer must be between 0 and 100
Only an integer value may be entered in this field.

Please write your answer here:

Your initial probability answer was: {INSERTANS:137236X3506X27729}

A troponin level was drawn. The value is greater than 30, which is reported as abnormal.

Now what is the probability that this patient has an acute myocardial infarction? *

Each answer must be between 0 and 100
Only an integer value may be entered in this field.

Please write your answer here:

© 2019 Brush JE Jr et al. JAMA Network Open.

Experts indicate that the correct answer for this case is: *an acute myocardial infarction*

© 2019 Brush JE Jr et al. JAMA Network Open.

Case 25 of 27

A 50-year-old man presents to the ED with chest pain. He has a history of hypertension, but has not been compliant with his medications. He developed chest pain starting yesterday. The pain is described as a severe sharp, tearing pain in the mid chest radiating to the left arm and back. The pain lasted about 30 minutes yesterday and resolved spontaneously. This morning, it recurred and is now severe, 9/10 in intensity. It is associated with mild nausea and diaphoresis.

PMH is remarkable for hypertension. He was prescribed a thiazide diuretic and beta blocker, which he stopped taking about a month ago. He hasn't followed up with his primary care doctor in over 6 months.

On exam, HR=100, BP=200/110, RR=28. He appears acutely uncomfortable. Lungs are clear. Cardiac exam reveals a regular rate and rhythm and no murmurs. Pulses are equal throughout.

On a scale of 0 (very IMprobable) to 100 (very PROBABLE), what is the probability that this patient has an acute myocardial infarction? *

Each answer must be between 0 and 100
Only an integer value may be entered in this field.

Please write your answer here:

Your initial probability answer was: {INSERTANS:137236X3507X27732}

A troponin level was drawn. The value is greater than 30, which is reported as abnormal.

Now what is the probability that this patient has an acute myocardial infarction? *

Each answer must be between 0 and 100
Only an integer value may be entered in this field.

Please write your answer here:

© 2019 Brush JE Jr et al. JAMA Network Open.

Experts disagree what the diagnosis is at this point; *it could either be an acute myocardial infarction or an aortic dissection.*

© 2019 Brush JE Jr et al. JAMA Network Open.

Case 26 of 27

A 35-year-old man presents to the ED with a 3-day history of chest pain. About a week ago, he had an upper respiratory illness, with a sore throat, non-productive cough, and a fever as high as 101 degrees (F). He saw his primary care physician and was prescribed ampicillin and Tylenol. His symptoms improved after about 3-4 days. Several days ago, he began experiencing chest pain described as a sharp pain in the left chest without radiation. The pain would occur several times daily without any particular precipitating factor. The pain seemed to worsen when he took a deep breath. He has noted mild shortness of breath because he has found it hard to take a deep breath.

PMH is unremarkable. He is otherwise healthy and takes no medications.

On examination, HR-100, BO=120/80, RR=24. He is in mild distress due to pain. HEENT is unremarkable. There is mild cervical adenopathy. Lungs are clear to auscultation. Cardiac exam reveals a regular rate and rhythm and a short mid-systolic sound that may be a murmur or a rub. There is no chest tenderness and the abdominal exam is benign.

On a scale of 0 (very IMprobable) to 100 (very PROBABLE), what is the probability that this patient has an acute myocardial infarction? *

Each answer must be between 0 and 100
Only an integer value may be entered in this field.

Please write your answer here:

Your initial probability answer was: {INSERTANS:137236X3508X27735}

A troponin level was drawn. The value is less than 30, which is reported as normal.

Now what is the probability that this patient has an acute myocardial infarction? *

Each answer must be between 0 and 100
Only an integer value may be entered in this field.

Please write your answer here:

© 2019 Brush JE Jr et al. JAMA Network Open.

Experts indicate that the correct answer for this case is: *pericarditis*

© 2019 Brush JE Jr et al. JAMA Network Open.

Case 27 of 27

An 80 year-old woman presents to the ED with chest pain. She has a longstanding history of hypertension, obesity, type 2 diabetes mellitus and continues to smoke 1 ppd. Five years ago, she had a pacer placed for symptomatic bradycardia. This morning while having her morning coffee and a cigarette, she developed the gradual onset of a pain in her mid-chest described as a moderately severe tightness, radiating to her left shoulder blade. She felt mildly nauseated, but otherwise, there were no other associated symptoms. She told her husband who immediately called 911. EKG in route revealed normal sinus rhythm and 100% ventricular paced rhythm.

PMH is remarkable for pacer placement, hypertension, diabetes, and hyperlipidemia. She takes metformin, amlodipine, losartan, simvastatin, and aspirin.

On examination, HR=110, BP=150/90, RR=28. She appears uncomfortable. Lungs are clear, cardiac exam reveals a regular rate and rhythm. Abdomen is benign.

On a scale of 0 (very IMprobable) to 100 (very PROBABLE), what is the probability that this patient has an acute myocardial infarction? *

Each answer must be between 0 and 100
Only an integer value may be entered in this field.

Please write your answer here:

Your initial probability answer was: {INSERTANS:137236X3509X27738}

A troponin level was drawn. The value is greater than 30, which is reported as abnormal.

Now what is the probability that this patient has an acute myocardial infarction? *

Each answer must be between 0 and 100
Only an integer value may be entered in this field.

Please write your answer here:

© 2019 Brush JE Jr et al. JAMA Network Open.

Experts indicate that the correct answer for this case is: *an acute myocardial infarction*

© 2019 Brush JE Jr et al. JAMA Network Open.

Submit your survey.
Thank you for completing this survey.

© 2019 Brush JE Jr et al. JAMA Network Open.

Control Group

1. Consent Form
2. Instructions
3. Reading Material

© 2019 Brush JE Jr et al. JAMA Network Open.

EBM Study C2

Experiential /Reading condition

There are 26 questions in this survey

Please review the following consent form.

© 2019 Brush JE Jr et al. JAMA Network Open.

Questionnaire Consent Form

Eastern Virginia Medical School (EVMS) Institutional Review Board

Study Title: ***A Study to Evaluate Strategies for Teaching Effective Use of Diagnostic Tests (EBM)***

Name of Investigator: John Brush, MD

Sponsor: None

You are being asked to participate in a research study involving the collection of information in the form of questionnaire. The purpose of the research project is to examine the effective use of diagnostic tests in clinical reasoning among pre-clinical medical students.

The study involves an online 90-minute session. You will receive standard instructions about how to use diagnostic tests, then either a) more detailed conceptual instruction about the relation between diagnostic test characteristics and disease probability, or b) a series of systematically chosen cases showing how test characteristics can influence probability of disease or c) a control condition where you will learn more about the specific conditions used in the study. You will then be asked to interpret 20 cases by providing a diagnosis and probability before and after receiving the results of the test.

You will reimbursed $30 for your participation. There are no additional costs to you associated with taking part in this study.

A risk associated with allowing your data to be saved is the release of personal information from your study record. We will strive to protect your records so that your personal information (like name, address, social security number and phone number) will remain private. There also may be other risks that are unknown and we cannot predict.. Your academic standing will not be effected by participation, or choosing not to participate. Responses to study questions will not become part of your academic record.

Although the results of this research may not benefit you directly, they may be made available upon request. You have the potential to receive educational benefit from participation. Your participation will contribute to our understanding of this skill, and may benefit future trainees. However, participation will not provide any benefit to your academic standing or increased merit compared to students not participating.

In conducting this research study, the data from the study will be analyzed statistically to test study hypotheses, and will be retained in an anonymized form for 10 years. No individual identifiers will be present in the database. Your protected health information (PHI), which includes personal information about you only be collected.

Your study records may be reviewed and/or copied in order to meet state and/or federal regulations. Reviewers may include, for example, Eastern Virginia Medical School Institutional Review Board, study investigator, John Brush, MD and his research team, and the research collaborators at McMaster University.

Information learned from this research may be used in reports, presentations and publications. None of these will personally identify you.

Taking part in this study is your choice. If you decide not to take part, your choice will not affect any medical benefits to which you are entitled. You may choose to leave the study at any time by notifying the study investigator. The study investigator may decide to take you off this study if you revoke your authorization.

We will tell you about new information that may affect your health, welfare, or willingness to stay in this study.

© 2019 Brush JE Jr et al. JAMA Network Open.

In the event of injury resulting from this research study, Eastern Virginia Medical School (EVMS) provides no financial compensation plan or free medical care.

If you have any questions pertaining to this research you may contact John Brush, MD or Judith Taylor-Fishwick at DiagnosticReasoningStudy@evms.edu, 757-446-8475. If you believe you have suffered an injury as a result of your participation in this study, you should contact the principal investigator, John Brush, MD at 757-446-8475. You may also contact Betsy Conner, director, EVMS Human Subjects Protection Program and IRB office at (757) 446-5854. If you have any questions pertaining to your rights as a research subject, you may contact a member of the Institutional Review Board through the Institutional Review Board office at (757) 446-8423.

You are being asked to participate in the above research study, which is being conducted at Eastern Virginia Medical School (EVMS), where you are an employee or student. The research study has been described to you, in writing, on this electronic consent form. You have also had the opportunity to ask the investigators conducting this study any questions that you may have regarding participation in this study.

The purpose of this consent form is to inform you that you have the right to choose not to participate in this research study. If you choose not to participate, or to withdraw at any time, it will not affect your standing as an employee or student.

If you are an employee, your participation will not place you in good favor with the investigator, your supervisor, or EVMS (e.g., increase in salary, promotion, extra vacation, or the like). Not participating will not adversely affect your employment with EVMS, in particular the position that you currently hold. If you are a student, your participation will not place you in good favor with the investigator or other faculty (e.g., receiving better grades, recommendations, employment). Also, not participating in this study will not adversely affect your relationship with the investigator or other faculty.

By clicking "Agree", you are consenting to this study. You are able to print out a copy of this consent form for your records.

Note: Do NOT click on the "BACK" button in your browser while completing this study. *

Please choose **all** that apply:

☐ **Agree**

If you do NOT agree, you can exit this study by closing your browser or clicking on the "exit and clear survey" button below.

© 2019 Brush JE Jr et al. JAMA Network Open.

Instructions

In this session, you will review a series of high quality readings on the presentation and diagnosis of three clinical topics: *congestive heart failure, pulmonary embolism* and *acute coronary syndrome*. For each diagnosis, you will view one clinical case to practice interpreting diagnostic tests.

YOU MUST COMPLETE THIS READING MODULE IN ONE SITTING. If you don't have approximately 30 minutes at the moment, please click on the "Exit and clear survey" button at the bottom of this page and come back to it later using the same link.

Thanks.

© 2019 Brush JE Jr et al. JAMA Network Open.

Reading Module: Congestive Heart Failure (CHF)

© 2019 Brush JE Jr et al. JAMA Network Open.

Heart Failure Clinical Presentation

History

In evaluating patients with heart failure, the clinician should ask about the following comorbidities and/or risk factors:

- **Myopathy**
- **Previous myocardial infarction**
- **Valvular heart disease, familial heart disease**
- **Alcohol use**
- **Hypertension**
- **Diabetes**
- **Dyslipidemia**
- **Coronary/peripheral vascular disease**
- **Sleep-disordered breathing**
- **Collagen vascular disease, rheumatic fever**
- **Pheochromocytoma**
- **Thyroid disease**
- **Substance abuse (previous/current history)**
- **History of chemotherapy/radiation to the chest**

The New York Heart Association (NYHA) classification of heart failure is widely used in practice and in clinical studies to quantify clinical assessment of heart failure (see Heart Failure Criteria, Classification, and Staging). Breathlessness, a cardinal symptom of left ventricular (LV) failure, may manifest with progressively increasing severity as the following:

- **Exertional dyspnea**
- **Orthopnea**
- **Paroxysmal nocturnal dyspnea**
- **Dyspnea at rest**
- **Acute pulmonary edema**

Other cardiac symptoms of heart failure include chest pain/pressure and palpitations. Common noncardiac signs and symptoms of heart failure include anorexia, nausea, weight loss, bloating, fatigue, weakness, oliguria, nocturia, and cerebral symptoms of varying severity, ranging from anxiety to memory impairment and confusion. Findings from the Framingham Heart Study suggested that subclinical cardiac dysfunction and noncardiac comorbidities are associated with increased incidence of heart failure, supporting the idea that heart failure is a progressive syndrome and that noncardiac factors are extremely important. [27, 28, 52]

Older patients with heart failure frequently have preserved ejection fraction and an atypical and/or delayed presentation. [53]

Exertional dyspnea

The principal difference between exertional dyspnea in patients who are healthy and exertional dyspnea in patients with heart failure is the degree of activity necessary to induce the symptom. As heart failure first develops, exertional dyspnea may simply appear to be an aggravation of the breathlessness that occurs in healthy persons

© 2019 Brush JE Jr et al. JAMA Network Open.

during activity, but as LV failure advances, the intensity of exercise resulting in breathlessness progressively declines; however, subjective exercise capacity and objective measures of LV performance at rest in patients with heart failure are not closely correlated. Exertional dyspnea, in fact, may be absent in sedentary patients.

Orthopnea

Orthopnea is an early symptom of heart failure and may be defined as dyspnea that develops in the recumbent position and is relieved with elevation of the head with pillows. As in the case of exertional dyspnea, the change in the number of pillows required is important. In the recumbent position, decreased pooling of blood in the lower extremities and abdomen occurs. Blood is displaced from the extrathoracic compartment to the thoracic compartment. The failing LV, operating on the flat portion of the Frank-Starling curve, cannot accept and pump out the extra volume of blood delivered to it without dilating. As a result, pulmonary venous and capillary pressures rise further, causing interstitial pulmonary edema, reduced pulmonary compliance, increased airway resistance, and dyspnea.

Orthopnea occurs rapidly, often within a minute or two of recumbency, and develops when the patient is awake. Orthopnea may occur in any condition in which the vital capacity is low. Marked ascites, regardless of its etiology, is an important cause of orthopnea. In advanced LV failure, orthopnea may be so severe that the patient cannot lie down and must sleep sitting up in a chair or slumped over a table.

Cough, particularly during recumbency, may be an "orthopnea equivalent." This nonproductive cough may be caused by pulmonary congestion and is relieved by the treatment of heart failure.

Paroxysmal nocturnal dyspnea

Paroxysmal nocturnal dyspnea usually occurs at night and is defined as the sudden awakening of the patient, after a couple of hours of sleep, with a feeling of severe anxiety, breathlessness, and suffocation. The patient may bolt upright in bed and gasp for breath. Bronchospasm increases ventilatory difficulty and the work of breathing and is a common complicating factor of paroxysmal nocturnal dyspnea. On chest auscultation, the bronchospasm associated with a heart failure exacerbation can be difficult to distinguish from an acute asthma exacerbation, although other clues from the cardiovascular examination should lead the examiner to the correct diagnosis. Both types of bronchospasm can be present in a single individual.

In contrast to orthopnea, which may be relieved by immediately sitting up in bed, paroxysmal nocturnal dyspnea may require 30 minutes or longer in this position for relief. Episodes may be so frightening that the patient may be afraid to resume sleeping, even after the symptoms have subsided.

Dyspnea at rest

Dyspnea at rest in heart failure is the result of the following mechanisms:

- **Decreased pulmonary function secondary to decreased compliance and increased airway resistance**
- **Increased ventilatory drive secondary to hypoxemia due to increased pulmonary capillary wedge pressure (PCWP); ventilation/perfusion (V/Q) mismatching due**

© 2019 Brush JE Jr et al. JAMA Network Open.

to increased PCWP and low cardiac output; and increased carbon dioxide production
- **Respiratory muscle dysfunction, with decreased respiratory muscle strength, decreased endurance, and ischemia**

Pulmonary edema

Acute pulmonary edema is defined as the sudden increase in PCWP (usually >25 mm Hg) as a result of acute and fulminant LV failure. It is a medical emergency and has a very dramatic clinical presentation. The patient appears extremely ill, poorly perfused, restless, sweaty, tachypneic, tachycardic, hypoxic, and coughing, with an increased work of breathing and using respiratory accessory muscles and with frothy sputum that on occasion is blood tinged.

Chest pain/pressure and palpitations

Chest pain/pressure may occur as a result of either primary myocardial ischemia from coronary disease or secondary myocardial ischemia from increased filling pressure, poor cardiac output (and, therefore, poor coronary diastolic filling), or hypotension and hypoxemia.

Palpitations are the sensation a patient has when the heart is racing. It can be secondary to sinus tachycardia due to decompensated heart failure, or more commonly, it is due to atrial or ventricular tachyarrhythmias.

Fatigue and weakness

Fatigue and weakness are often accompanied by a feeling of heaviness in the limbs and are generally related to poor perfusion of the skeletal muscles in patients with a lowered cardiac output. Although they are generally a constant feature of advanced heart failure, episodic fatigue and weakness are also common in earlier stages.

Nocturia and oliguria

Nocturia may occur relatively early in the course of heart failure. Recumbency reduces the deficit in cardiac output in relation to oxygen demand, renal vasoconstriction diminishes, and urine formation increases. Nocturia may be troublesome for patients with heart failure because it may prevent them from obtaining much-needed rest. Oliguria is a late finding in heart failure, and it is found in patients with markedly reduced cardiac output from severely reduced LV function.

Cerebral symptoms

The following may occur in elderly patients with advanced heart failure, particularly in those with cerebrovascular atherosclerosis:

- **Confusion**
- **Memory impairment**
- **Anxiety**
- **Headaches**
- **Insomnia**
- **Bad dreams or nightmares**
- **Rarely, psychosis with disorientation, delirium, or hallucinations**

© 2019 Brush JE Jr et al. JAMA Network Open.

Heart Failure Clinical Presentation

Physical Examination

Patients with mild heart failure appear to be in no distress after a few minutes of rest, but they may be obviously dyspneic during and immediately after moderate activity. Patients with left ventricular (LV) failure may be dyspneic when lying flat without elevation of the head for more than a few minutes. Those with severe heart failure appear anxious and may exhibit signs of air hunger in this position.

Patients with a recent onset of heart failure are generally well nourished, but those with chronic severe heart failure are often malnourished and sometimes even cachectic. Chronic marked elevation of the systemic venous pressure may produce exophthalmos and severe tricuspid regurgitation, and it may lead to visible pulsation of the eyes and of the neck veins. Central cyanosis, icterus, and malar flush may be evident in patients with severe heart failure.

In mild or moderate heart failure, stroke volume is normal at rest; in severe heart failure, it is reduced, as reflected by a diminished pulse pressure and a dusky discoloration of the skin. With very severe heart failure, particularly if cardiac output has declined acutely, systolic arterial pressure may be reduced. The pulse may be weak, rapid, and thready; the proportional pulse pressure (pulse pressure/systolic pressure) may be markedly reduced. The proportional pulse pressure correlates reasonably well with cardiac output. In one study, when pulse pressure was less than 25%, it usually reflected a cardiac index of less than 2.2 L/min/m^2. [54]

Ascites occurs in patients with increased pressure in the hepatic veins and in the veins draining into the peritoneum; it usually reflects long-standing systemic venous hypertension. Fever may be present in severe decompensated heart failure because of cutaneous vasoconstriction and impairment of heat loss.

Increased adrenergic activity is manifested by tachycardia, diaphoresis, pallor, peripheral cyanosis with pallor and coldness of the extremities, and obvious distention of the peripheral veins secondary to venoconstriction. Diastolic arterial pressure may be slightly elevated.

Rales heard over the lung bases are characteristic of heart failure that is of at least moderate severity. With acute pulmonary edema, rales are frequently accompanied by wheezing and expectoration of frothy, blood-tinged sputum. The absence of rales *does not* exclude elevation of pulmonary capillary pressure due to LV failure.

Systemic venous hypertension is manifested by jugular venous distention. Normally, jugular venous pressure declines with respiration; however, it increases in patients with heart failure, a finding known as the Kussmaul sign (also found in constrictive pericarditis). This reflects an increase in right atrial pressure and, therefore, right-sided heart failure. In general, elevated jugular venous pressure is the most reliable indicator of fluid volume overload in older patients, and thorough evaluation is needed. [53]

The hepatojugular reflux is the distention of the jugular vein induced by applying manual pressure over the liver; the patient's torso should be positioned at a 45° angle. The hepatojugular reflux occurs in patients with elevated left-sided filling

© 2019 Brush JE Jr et al. JAMA Network Open.

pressures and reflects elevated capillary wedge pressure and left-sided heart failure.

Although edema is a cardinal manifestation of heart failure, it does not correlate well with the level of systemic venous pressure. In patients with chronic LV failure and low cardiac output, extracellular fluid volume may be sufficiently expanded to cause edema in the presence of only slight elevations in systemic venous pressure. Usually, a substantial gain of extracellular fluid volume (i.e., a minimum of 5 L in adults) must occur before peripheral edema develops. Edema in the absence of dyspnea or other signs of LV or right ventricular (RV) failure is not solely indicative of heart failure and can be observed in many other conditions, including chronic venous insufficiency, nephrotic syndrome, or other syndromes of hypoproteinemia or osmotic imbalance.

Hepatomegaly is prominent in patients with chronic right-sided heart failure, but it may occur rapidly in acute heart failure. When hepatomegaly occurs acutely, the liver is usually tender. In patients with considerable tricuspid regurgitation, a prominent systolic pulsation of the liver, attributable to an enlarged right atrial V wave, is often noted. A presystolic pulsation of the liver, attributable to an enlarged right atrial A wave, can occur in tricuspid stenosis, constrictive pericarditis, restrictive cardiomyopathy involving the right ventricle, and pulmonary hypertension (primary or secondary).

Hydrothorax is most commonly observed in patients with hypertension involving both the systemic and pulmonary circulation. It is usually bilateral, although when unilateral, it is usually confined to the right side of the chest. When hydrothorax develops, dyspnea usually intensifies because of further reductions in vital capacity.

Cardiac findings

Protodiastolic (S_3) gallop is the earliest cardiac physical finding in decompensated heart failure in the absence of severe mitral or tricuspid regurgitation or left-to-right shunts. The presence of an S_3 gallop in adults is important, pathologic, and often the most apparent finding on cardiac auscultation in patients with significant heart failure.

Cardiomegaly is a nonspecific finding that nonetheless occurs in most patients with chronic heart failure. Notable exceptions include heart failure from acute myocardial infarction, constrictive pericarditis, restrictive cardiomyopathy, valve or chordae tendineae rupture, or heart failure due to tachyarrhythmias or bradyarrhythmias.

Pulsus alternans (during pulse palpation, this is the alternation of one strong and one weak beat without a change in the cycle length) occurs most commonly in heart failure due to increased resistance to LV ejection, as occurs in hypertension, aortic stenosis, coronary atherosclerosis, and dilated cardiomyopathy. Pulsus alternans is usually associated with an S_3 gallop, signifies advanced myocardial disease, and often disappears with treatment of heart failure.

Accentuation of the P_2 heart sound is a cardinal sign of increased pulmonary artery pressure; it disappears or improves after treatment of heart failure. Mitral and tricuspid regurgitation murmurs are often present in patients with decompensated heart failure because of ventricular dilatatation. These murmurs often disappear or diminish when compensation is restored. Note that the correlation is poor between the intensity of the murmur of mitral regurgitation and its significance in patients

© 2019 Brush JE Jr et al. JAMA Network Open.

with heart failure. Severe mitral regurgitation may be accompanied by an unimpressively soft murmur.

Cardiac cachexia is found in long-standing heart failure, particularly of the RV, because of anorexia from hepatic and intestinal congestion and sometimes because of digitalis toxicity. Occasionally, impaired intestinal absorption of fat occurs and, rarely, protein-losing enteropathy occurs. Patients with heart failure may also exhibit increased total metabolism secondary to augmentation of myocardial oxygen consumption, excessive work of breathing, low-grade fever, and elevated levels of circulating tumor necrosis factor (TNF).

© 2019 Brush JE Jr et al. JAMA Network Open.

Heart Failure Differential Diagnoses

Diagnostic Considerations

Cardiogenic and noncardiogenic pulmonary edema

Atypical presentations

Many classes of disorders can result in increased cardiac demand or impaired cardiac function. Cardiac causes include arrhythmias (tachycardia or bradycardia), structural heart disease, and myocardial dysfunction (systolic or diastolic). Noncardiac causes include processes that increase the preload (volume overload), increase the afterload (hypertension), reduce the oxygen-carrying capacity of the blood (anemia), or increase demand (sepsis). For example, renal failure can result in heart failure due to fluid retention and anemia. Lymphatic obstruction and venous obstruction syndromes can also cause edema-forming states, and obesity-hypoventilation syndrome (OHS) can lead to right-sided heart failure with right ventricular hypertrophy.

Diastolic heart failure may be the most common form of heart failure in the US population. [21] Alterations in ventricular-arterial coupling appear to have a key role in impaired hemodynamic response to exercise, but the diagnosis of diastolic heart failure cannot be excluded even in the presence of normal diastolic function at rest. [21]

Heart failure should also be differentiated from the pulmonary edema that is associated with injury to the alveolar-capillary membrane caused by diverse etiologies (i.e., noncardiogenic pulmonary edema, adult respiratory distress syndrome [ARDS]). Increased capillary permeability is observed in trauma, hemorrhagic shock, sepsis, respiratory infections, administration of various drugs, and ingestion of toxins (eg, heroin, cocaine, toxic gases). With the advent of natriuretic peptide testing, differentiating cardiac from noncardiac causes of pulmonary edema has improved. [60, 61]

Several features may differentiate cardiogenic from noncardiogenic pulmonary edema. In heart failure, a history of an acute cardiac event or of progressive symptoms of heart failure is usually present. The physical examination may yield clues to acute heart failure. Findings such as an S_3 gallop and elevated jugular venous pulsation are highly specific for acute heart failure, but their low sensitivity makes them less-than-ideal screening tools. [54, 62]

Patients with noncardiogenic pulmonary edema may have clinical features similar to those with cardiogenic pulmonary edema but will often lack an S_3 gallop and jugular venous distention. The differentiation is often made based on pulmonary capillary wedge pressure (PCWP) measurements from invasive hemodynamic monitoring. Left ventricular filling pressures measured by PCWP are the single most reliable hemodynamic measure that predicts a fatal outcome in patients with acute heart failure. PCWP is generally more than 18 mm Hg in heart failure and less than 18 mm Hg in noncardiogenic pulmonary edema, but superimposition of chronic pulmonary vascular disease can make this distinction more difficult to discern.

Heart failure, in particular right-sided heart failure, can present as an abdominal

© 2019 Brush JE Jr et al. JAMA Network Open.

syndrome with nausea, vomiting, right-sided abdominal pain (as a sign of liver congestion), bloating, anorexia, and significant weight loss. In advanced cases, patients can appear jaundiced because of cardiac cirrhosis. Constipation is a common complaint among patients with heart failure, and it can be a manifestation of decreased intestinal transit secondary to poor perfusion. In very severe cases of cardiogenic shock, an individual can present with severe abdominal pain mimicking bowel obstruction, perforation, acute abdomen, and peritonitis as a manifestation of severe intestinal ischemia and possible infarction.

In elderly patients, fatigue and confusion can sometimes be the first symptoms of heart failure, which is related to a decrease in cardiac output. The mnemonic DEFEAT-HF consists of five steps that may be helpful in the diagnosis and management of heart failure in the older population: diagnosis, etiology, fluid volume, ejection fraction, and therapy. [53]

© 2019 Brush JE Jr et al. JAMA Network Open.

Case Instructions

For the following case and the two other later cases, you will be asked for a probability (1-100) of the diagnosis. Then, a test result will be given and you will be asked for a post-test probability of the diagnosis.

© 2019 Brush JE Jr et al. JAMA Network Open.

Case 1

An 80 year-old woman is presents to the ED with acute shortness of breath. She has had several myocardial infarctions in the past, has a history of chronic systolic heart failure and presents acutely short of breath. She lives alone and usually her daughter brings her meals to her. Over the past week, however, her daughter has been out of town and the family arranged for a neighbor to prepare the patient's meals. The neighbor supplied canned vegetables and soups and the patient noticed that the food was more salty than she was used to. She has been quite thirsty and increased her fluid intake over the past week. The day prior to admission she became more short of breath and she was unable to sleep last night due to difficulty breathing. She noticed that her ankles and hands have become swollen recently. She denies chest pain or palpitations.

PMH is remarkable for a history of coronary artery disease, hypertension, and chronic systolic heart failure. Her medications are carvedilol, lisinipril, and furosemide, which she has been taking regularly.

The patient is widowed. She formerly worked as a hairdresser. She does not smoke or drink alcohol.

On exam, HR=105, BP=100/60, RR=32. She is elderly, frail, and acutely short of breath. HEENT reveals bluish lips. Neck exam reveals jugular venous distension. Lungs reveal wet crackles throughout. She has a tachycardic heart rhythm and an S3 gallop. The abdomen is slightly distended and benign. There is 1+ bilateral pitting edema.

On a scale of 0 (very IMprobable) to 100 (very PROBABLE), what is the probability that this patient has congestive heart failure? *

Each answer must be between 0 and 100
Only an integer value may be entered in this field.

Please write your answer here:

Your initial probability answer was: {INSERTANS:485846X3476X27637}

A chest x-ray is performed. The radiologist interprets the x-ray as positive for pulmonary venous congestion.

Now what is the probability that the patient has congestive heart failure? *

Each answer must be between 0 and 100
Only an integer value may be entered in this field.

Please write your answer here:

Experts indicate that the correct answer for this case is: *congestive heart failure*

© 2019 Brush JE Jr et al. JAMA Network Open.

Reading Module: Pulmonary Embolism (PE)

© 2019 Brush JE Jr et al. JAMA Network Open.

Pulmonary Embolism

History, Physical Examination, Complications

Physical examination findings are quite variable in pulmonary embolism and, for convenience, may be grouped into four categories as follows:

- **Massive pulmonary embolism**
- **Acute pulmonary infarction**
- **Acute embolism without infarction**
- **Multiple pulmonary emboli or thrombi**

The presentation of pulmonary embolism may vary from sudden catastrophic hemodynamic collapse to gradually progressive dyspnea. (Prior poor cardiopulmonary status of the patient is an important factor leading to hemodynamic collapse.) Most patients with pulmonary embolism have no obvious symptoms at presentation. In contrast, patients with symptomatic DVT commonly have pulmonary embolism confirmed on diagnostic studies in the absence of pulmonary symptoms. Sickle cell disease often creates a diagnostic difficulty with regard to pulmonary embolism. A chest infection is often the presenting symptom.

Patients with pulmonary embolism may present with atypical symptoms. In such cases, strong suspicion of pulmonary embolism based on the presence of risk factors can lead to consideration of pulmonary embolism in the differential diagnosis. These symptoms include the following:

- **Seizures**
- **Syncope**
- **Abdominal pain**
- **Fever**
- **Productive cough**
- **Wheezing**
- **Decreasing level of consciousness**
- **New onset of atrial fibrillation**
- **Flank pain [1]**
- **Delirium (in elderly patients) [2]**

The diagnosis of pulmonary embolism should be sought actively in patients with respiratory symptoms unexplained by an alternative diagnosis. The symptoms of pulmonary embolism are nonspecific; therefore, a high index of suspicion is required, particularly when a patient has risk factors for the condition.

Acute respiratory consequences of pulmonary embolism include the following:

- **Increased alveolar dead space**
- **Hypoxemia**
- **Hyperventilation**

In patients with recognized pulmonary embolism, the incidence of physical signs has been reported as follows:

- **Tachypnea (respiratory rate >16/min) - 96%**

© 2019 Brush JE Jr et al. JAMA Network Open.

- **Rales - 58%**
- **Accentuated second heart sound - 53%**
- **Tachycardia (heart rate >100/min) - 44%**
- **Fever (temperature >37.8°C [100.04ºF]) - 43%**
- **Diaphoresis - 36%**
- **S $_3$ or S $_4$ gallop - 34%**
- **Clinical signs and symptoms suggesting thrombophlebitis - 32%**
- **Lower extremity edema - 24%**
- **Cardiac murmur - 23%**
- **Cyanosis - 19%**

The PIOPED study reported the following incidence of common symptoms of pulmonary embolism [35] :

- **Dyspnea (73%)**
- **Pleuritic chest pain (66%)**
- **Cough (37%)**
- **Hemoptysis (13%)**

Fever of less than 39°C (102.2ºF) may be present in 14% of patients; however, temperature higher than 39.5°C (103.1º F) is not from pulmonary embolism. Chest wall tenderness upon palpation, without a history of trauma, may be the sole physical finding in rare cases.

Pleuritic chest pain without other symptoms or risk factors may be a presentation of pulmonary embolism. Pleuritic or respirophasic chest pain is a particularly worrisome symptom. Pleuritic chest pain is reported to occur in as many as 84% of patients with pulmonary emboli. Its presence suggests that the embolus is located more peripherally and thus may be smaller.

Pulmonary embolism has been diagnosed in 21% of young, active patients who come to emergency departments (EDs) complaining only of pleuritic chest pain. These patients usually lack any other classical signs, symptoms, or known risk factors for pulmonary thromboembolism. Such patients often are dismissed inappropriately with an inadequate workup and a nonspecific diagnosis, such as musculoskeletal chest pain or pleurisy.

Massive pulmonary embolism

Patients with massive pulmonary embolism are in shock. They have systemic hypotension, poor perfusion of the extremities, tachycardia, and tachypnea. In addition, patients appear weak, pale, sweaty, and oliguric and develop impaired mentation.

Signs of pulmonary hypertension, such as palpable impulse over the second left intercostal space, loud P_2, right ventricular S_3 gallop, and a systolic murmur louder on inspiration at left sternal border (tricuspid regurgitation), may be present.

Massive pulmonary embolism has been defined by hemodynamic parameters and evidence of myocardial injury rather than anatomic findings because the former is associated with adverse outcomes. [42] Although previous studies of CT scans in the diagnosis of pulmonary embolus suggested that central obstruction was not associated with adverse outcomes, a new multicenter study clarifies this observation.

© 2019 Brush JE Jr et al. JAMA Network Open.

Vedovati et al found no association between central obstruction and death or clinical deterioration in 579 patients with pulmonary embolus. [43] However, when a subset of 516 patients who were hemodynamically stable was assessed, central localization of emboli was found to be an independent mortality risk factor while distal localization was inversely associated with adverse events. Thus, anatomic findings by CT scan may be important in assessing risk in hemodynamically stable patients with pulmonary embolus.

Acute pulmonary infarction

Approximately 10% of patients have peripheral occlusion of a pulmonary artery, causing parenchymal infarction. These patients present with acute onset of pleuritic chest pain, breathlessness, and hemoptysis. Although the chest pain may be clinically indistinguishable from ischemic myocardial pain, normal ECG findings and no response to nitroglycerin rules out myocardial pain. Patients with acute pulmonary infarction have decreased excursion of the involved hemithorax, palpable or audible pleural friction rub, and even localized tenderness. Signs of pleural effusion, such as dullness to percussion and diminished breath sounds, may be present.

Acute embolism without infarction

Patients with acute embolism without infarction have nonspecific physical signs that may easily be secondary to another disease process. Tachypnea and tachycardia frequently are detected, pleuritic pain sometimes may be present, crackles may be heard in the area of embolization, and local wheeze may be heard rarely.

Multiple pulmonary emboli or thrombi

Patients with pulmonary emboli and thrombi have physical signs of pulmonary hypertension and cor pulmonale. Patients may have elevated jugular venous pressure, right ventricular heave, palpable impulse in the left second intercostal space, right ventricular S_3 gallop, systolic murmur over the left sternal border that is louder during inspiration, hepatomegaly, ascites, and dependent pitting edema. These findings are not specific for pulmonary embolism and require a high index of suspicion for pursuing appropriate diagnostic studies.

Pulmonary emboli in children

Many physical findings are typically less marked in children than they are in adults, presumably because children have greater hemodynamic reserve and, thus, are better able to tolerate the significant hemodynamic and pulmonary changes.

Because of the rarity of pulmonary emboli in children, these patients are probably underdiagnosed. For the same reason, much of the information pertaining to diagnosis and management of pulmonary embolism has been derived from adult practice.

Cough is present in approximately 50% of children with pulmonary emboli; tachypnea occurs with the same frequency. Hemoptysis is a feature in a minority of children with pulmonary emboli, occurring in about 30% of cases. Crackles are heard in a minority of cases.

Cyanosis and hypoxemia are not prominent features of pulmonary embolism. If

© 2019 Brush JE Jr et al. JAMA Network Open.

present, cyanosis suggests a massive embolism leading to a marked ventilation-perfusion (V/Q) mismatch and systemic hypoxemia. Some case reports have described massive pediatric pulmonary embolism with normal saturation.

A pleural rub is often associated with pleuritic chest pain and indicates an embolism in a peripheral location in the pulmonary vasculature. Signs that indicate pulmonary hypertension and right ventricular failure include a loud pulmonary component of the second heart sound, right ventricular lift, distended neck veins, and hypotension. An increase in pulmonary artery pressure is reportedly not evident until at least 60% of the vascular bed has been occluded.

A gallop rhythm signifies ventricular failure, while peripheral edema is a sign of congestive heart failure. Various heart murmurs may be audible, including a tricuspid regurgitant murmur signifying pulmonary hypertension.

Fever is an unusual sign that is nonspecific, and diaphoresis is a manifestation of sympathetic arousal. Signs of other organ involvement in patients with sickle cell disease would be elicited, such as sequestration crisis, priapism, anemia, and stroke.

© 2019 Brush JE Jr et al. JAMA Network Open.

Pulmonary Embolism Differential Diagnoses

Diagnostic Considerations

Sickle cell disease

The variability of presentation for pulmonary embolism sets the patient and clinician up for potentially missing the diagnosis. Such missed diagnoses occur in approximately 400,000 patients in the United States per year; approximately 100,000 deaths could be prevented with proper diagnosis and treatment.

The diagnostic challenge is that the "classic" presentation of the condition, with abrupt onset of pleuritic chest pain, shortness of breath, and hypoxia, is rarely seen. Studies of patients who died unexpectedly from pulmonary embolism have revealed that the patients complained of nagging symptoms, often for weeks, before dying. Forty percent of these patients had been seen by a physician in the weeks prior to their death. [7]

The differential diagnoses are extensive, and they should be considered carefully with any patient thought to have pulmonary embolism. These patients also should have an alternative diagnosis confirmed, or pulmonary embolism should be excluded, before discontinuing the workup. Additional problems to be considered include the following:

- **Musculoskeletal pain**
- **Pleuritis**
- **Pericarditis**
- **Salicylate intoxication**
- **Hyperventilation**
- **Silicone pulmonary embolism [44]**
- **Lung trauma**
- **Mediastinitis, acute**

Sickle cell disease often creates a diagnostic difficulty with regard to pulmonary embolism. A chest infection is often the presenting symptom. Hypoxemia, dehydration, and fever lead to intravascular sludging within pulmonary (among others) vasculature. This promotes a vicious cycle, further exacerbating local hypoxemia, ultimately leading to local tissue infarction. This process is further worsened by bone marrow infarction, which may cause release of fat emboli that lodge in the pulmonary circulation. [45]

© 2019 Brush JE Jr et al. JAMA Network Open.

Clinical Scoring Guidelines

A 2007 clinical practice guideline from the American Academy of Family Physicians (AAFP) and the American College of Physicians (ACP) recommends that validated clinical prediction rules be used to estimate pretest probability of pulmonary embolism (PE) and to interpret test results. [4] The guideline, *Current Diagnosis of Venous Thromboembolism in Primary Care*, advocates use of the Wells prediction rule for this purpose but notes that the Wells rule performs better in younger patients without comorbidities or a history of venous thromboembolism (VTE) than in other patients.

In 2015, the ACP released guidelines for the evaluation of patients with suspected acute PE, which included the following recommendations [107] :

- **Plasma D-dimer tests are more appropriate for those at intermediate risk for a PE, and no testing may be necessary for some patients at low risk.**
- **Use either the Wells or Geneva rules to choose tests based on a patient's risk for PE.**
- **If the patient is at low risk, clinicians should use the eight Pulmonary Embolism Rule-Out Criteria (PERC); if a patient meets all eight criteria, the risks of testing are greater than the risk for embolism, and no testing is needed.**
- **For patients at intermediate risk, or for those at low risk who do not meet all of the rule-out criteria, use a high-sensitivity plasma D-dimer test as the initial test.**
- **In patients older than 50 years, use an age-adjusted threshold (age × 10 ng/mL, rather than a blanket 500 ng/mL), because normal D-dimer levels increase with age.**
- **Patients with a D-dimer level below the age-adjusted cutoff should not receive any imaging studies.**
- **Patients with elevated D-dimer levels should then receive imaging.**
- **Patients at high risk should skip the D-dimer test and proceed to CT pulmonary angiography, because a negative D-dimer test does not eliminate the need for imaging in these patients.**
- **Clinicians should only obtain ventilation-perfusion scans in patients with a contraindication to CT pulmonary angiography or if CT pulmonary angiography is unavailable.**
- **Clinicians should use validated clinical prediction rules to estimate pretest probability in patients in whom acute PE is being considered.**

In contrast, the 2011 guidelines of the American College of Emergency Physicians (ACEP) find that either objective criteria or gestalt clinical assessment can be used to risk-stratify patients with suspected PE. There is insufficient evidence to support the preferential use of one method over another (level B). For patients with a low pretest probability for suspected PE, PERC may be used to exclude the diagnosis based on historical and physical examination data alone (level B). Other key recommendations include the following [108] :

- **Negative quantitative D-dimer assay results can be used to exclude PE in patients with a low pretest probability for PE (level A).**
- **Negative quantitative D-dimer assay results may be used to exclude PE in patients with an intermediate pretest probability for PE (level C).**

© 2019 Brush JE Jr et al. JAMA Network Open.

- **For patients with a low or PE unlikely (Wells score 4) pretest probability for PE who require additional diagnostic testing (e.g., positive D-dimer result, or highly sensitive D-dimer test not available), a negative, multidetector CT pulmonary angiogram alone can be used to exclude PE (level B).**
- **For patients with an intermediate or high pretest probability for PE and a negative CT pulmonary angiogram result in whom a clinical concern for PE still exists and CT venogram has not already been performed, consider additional diagnostic testing (e.g., D-dimer, lower extremity imaging, VQ scanning, traditional pulmonary arteriography) prior to exclusion of VTE disease (level C).**
- **Venous ultrasound may be considered as initial imaging in patients with obvious signs of deep venous thrombosis (DVT) for whom venous ultrasound is readily available, patients with relative contraindications for CT scan (e.g., borderline renal insufficiency, CT contrast agent allergy), and pregnant patients. A positive finding in a patient with symptoms consistent with PE can be considered evidence for diagnosis of VTE disease and may preclude the need for additional diagnostic imaging in the emergency department (level B).**

© 2019 Brush JE Jr et al. JAMA Network Open.

D-Dimer Follow-Up on Low-to-Moderate Pretest Probability

When clinical prediction rule results indicate that the patient has a low or moderate pretest probability of pulmonary embolism, D-dimer testing may be the next step. [3]

D-Dimer, a degradation product produced by plasmin-mediated proteases of cross-linked fibrin, is measured by a variety of assay types, including quantitative, semiquantitative, and qualitative rapid enzyme-linked immunosorbent assays (ELISAs); quantitative and semi-quantitative latex; and whole-blood assays. A systematic review of prospective studies of high methodologic quality concluded that the ELISAs—especially the quantitative rapid ELISA—dominate the comparative ranking among the D-dimer assays for sensitivity and negative likelihood ratio. [47] The quantitative rapid ELISA has a sensitivity of 0.95 and negative likelihood ratio of 0.13; the latter is similar to that for a normal to near-normal lung scan in patients with suspected pulmonary embolism.

Negative results on a high-sensitivity D-dimer test in a patient with a low pretest probability of pulmonary embolism indicate a low likelihood of venous thromboembolism and reliably exclude pulmonary embolism. A large, prospective, randomized trial found that in patients with a low probability of pulmonary embolism who had negative D-dimer results, forgoing additional diagnostic testing was not associated with an increased frequency of symptomatic venous thromboembolism during the subsequent 6 months. [48]

In a 2012 prospective cohort study, a Wells score of 4 or less combined with a negative qualitative D-dimer test was shown to safely exclude pulmonary embolism in primary care patients. [49]

D-dimer testing is most reliable for excluding pulmonary embolism in younger patients who have no associated comorbidity or history of venous thromboembolism and whose symptoms are of short duration. [4] However, it is of questionable value in patients who are older than 80 years, who are hospitalized, who have cancer, or who are pregnant, because nonspecific elevation of D-dimer concentrations is common in such patients.

D-dimer testing should not be used when the clinical probability of pulmonary embolism is high, because the test has low negative predictive value in such cases. [50]

Combining D-dimer results with measurement of the exhaled end-tidal ratio of carbon dioxide to oxygen ($etCO_2/O_2$) can be useful for diagnosis of pulmonary embolism. Kline et al found that, in moderate-risk patients with a positive D-dimer (>499 ng/mL), an $etCO_2/O_2$< 0.28 significantly increased the probability of finding segmental or larger pulmonary embolism on computed tomography multidetector-row pulmonary angiography, while an $etCO_2/O_2$) >0.45 predicted the absence of segmental or larger pulmonary embolism. [51]

Because of the poor specificity, positive D-dimer measurements are not helpful in confirming the diagnosis of venous thromboembolic disease. However, a positive D-dimer measurement may lead to consideration of venous thromboembolic disease

© 2019 Brush JE Jr et al. JAMA Network Open.

in the differential diagnosis in selected patients. In addition, the use of D-dimers in children is not well studied. A small pediatric series reported that D-dimer measurements are negative in 40% of patients. [29] A retrospective series reported an elevated D-dimer in 86% of patients at presentation. [17]

Pulmonary Embolism Workup

Electrocardiography

The most common ECG abnormalities in the setting of pulmonary embolism are tachycardia and nonspecific ST-T wave abnormalities. The finding of S_1 Q_3 T_3 is nonspecific and insensitive in the absence of clinical suspicion for pulmonary embolism. The classic findings of right heart strain and acute cor pulmonale are tall, peaked P waves in lead II (P pulmonale); right axis deviation; right bundle-branch block; an S_1 Q_3 T_3 pattern; or atrial fibrillation. Unfortunately, only 20% of patients with proven pulmonary embolism have any of these classic electrocardiographic abnormalities. If electrocardiographic abnormalities are present, they may be suggestive of pulmonary embolism, but the absence of such abnormalities has no significant predictive value.

© 2019 Brush JE Jr et al. JAMA Network Open.

Case 2

A 29-year-old woman presents complaining of chest heaviness. For the past three days she has complained of sinus congestion, a sore throat and a dry non-productive cough. She is worried that she might have caught something from her roommate, who has also been sick for the past few days. However, her inability to catch her breath is the reason that she is attending the emergency department. She denies any pleuritic chest pain or calf swelling. She was able to exercise yesterday without feeling short of breath. However, she kept coughing, so she gave up when people at the gym kept giving her a dirty look.

The patient is well without any other medical problems. She is a non-smoker. Her only medication is ibuprofen, which she has been taking for the last 3 days to address her sore throat and because “her body feels achy and run down.”

On examination, her temperature is 37.3C; pulse 76 / minute; blood pressure 110/76mmHg; and respiratory rate 18 / minute. Her heart rate is regular. She has normal pulses. There are no extra cardiac sounds or murmurs. Her chest exam is normal with an occasional transmitted upper respiratory sound. She has an inflamed oropharynx with without evidence of purulence. Her calves are symmetric, non-tender and not swollen.

On a scale of 0 (very IMprobable) to 100 (very PROBABLE), what is the probability that this patient has a pulmonary embolism? *

Each answer must be between 0 and 100
Only an integer value may be entered in this field.

Please write your answer here:

Your initial probability answer was: {INSERTANS:485846X3479X27646}

A quantitative D-dimer is measured. The value is less than 500, which is reported as negative.

Now what is the probability that this patient has a pulmonary embolism? *

Each answer must be between 0 and 100
Only an integer value may be entered in this field.

Please write your answer here:

Experts indicate that the correct answer for this case is: *an upper respiratory infection (URI).*

© 2019 Brush JE Jr et al. JAMA Network Open.

Reading Module: Acute Coronary Syndrome (ACS)

Acute Coronary Syndrome

Clinical Presentation

History

The severity and duration of coronary artery obstruction, the volume of myocardium affected, the level of demand, and the ability of the rest of the heart to compensate are major determinants of a patient's clinical presentation and outcome. A patient may present to the ED because of a change in pattern or severity of symptoms.

Typically, angina is a symptom of myocardial ischemia that appears in circumstances of increased oxygen demand. It is usually described as a sensation of chest pressure or heaviness that is reproduced by activities or conditions that increase myocardial oxygen demand. A new case of angina is more difficult to diagnose because symptoms are often vague and similar to those caused by other conditions (e.g., indigestion, anxiety).

However, not all patients experience chest pain. They may present with only neck, jaw, ear, arm, or epigastric discomfort. Some patients, including some who are elderly or who have diabetes, present with no pain, complaining only of episodic shortness of breath, severe weakness, light-headedness, diaphoresis, or nausea and vomiting. Elderly persons may also present only with altered mental status. Those with pre-existing altered mental status or dementia may have no recollection of recent symptoms and may have no complaints.

In addition, evidence exists that women more often have coronary events without typical symptoms, which may explain the frequent failure of clinicians to initially diagnose ACS in women.

A summary of patient complaints is as follows:

- **Palpitations**
- **Pain, which is usually described as pressure, squeezing, or a burning sensation across the precordium and may radiate to the neck, shoulder, jaw, back, upper abdomen, or either arm**
- **Exertional dyspnea that resolves with pain or rest**
- **Diaphoresis from sympathetic discharge**
- **Nausea from vagal stimulation**
- **Decreased exercise tolerance**

Stable angina involves episodic pain lasting 5-15 minutes, is provoked by exertion, and is relieved by rest or nitroglycerin. In unstable angina, patients have increased risk for adverse cardiac events, such as myocardial infarction or death. New-onset exertional angina can occur at rest and is of increasing frequency or duration or is refractory to nitroglycerin. Variant angina (Prinzmetal angina) occurs primarily at rest, is triggered by smoking, and is thought to be due to coronary vasospasm.

© 2019 Brush JE Jr et al. JAMA Network Open.

Acute Coronary Syndrome Clinical Presentation

Physical Examination

Physical examination results are frequently normal. If chest pain is ongoing, the patient will usually lie quietly in bed and may appear anxious, diaphoretic, and pale. Physical findings can vary from normal to any of the following:

- **Hypotension - Indicates ventricular dysfunction due to myocardial ischemia, infarction, or acute valvular dysfunction**
- **Hypertension - May precipitate angina or reflect elevated catecholamine levels due to anxiety or to exogenous sympathomimetic stimulation**
- **Diaphoresis**
- **Pulmonary edema and other signs of left heart failure**
- **Extra-cardiac vascular disease**
- **Jugular venous distention**
- **Cool, clammy skin and diaphoresis in patients with cardiogenic shock**

In addition, a third heart sound (S_3) may be present, and frequently, a fourth heart sound (S_4) exists. The latter is especially prevalent in patients with inferior-wall ischemia and may be heard in patients with ischemia or systolic murmur secondary to mitral regurgitation

A systolic murmur related to dynamic obstruction of the left ventricular (LV) outflow tract may also occur. It is caused by hyperdynamic motion of the basal left ventricular myocardium and may be heard in patients with an apical infarct.

A new murmur may reflect papillary muscle dysfunction. Rales on pulmonary examination may suggest LV dysfunction or mitral regurgitation.

Patients who present to the ED with chest pain who have a low short-term risk of a major adverse cardiac event must be identified to facilitate early discharge in order to avoid lengthy and costly hospital stays. [14] The ASPECT study tested a 2-hour, accelerated diagnostic protocol (ADP) that included the use of a structured pretest probability scoring method, electrocardiography, and a point-of-care biomarker panel that included troponin, creatine kinase MB, and myoglobin levels. The study suggests that ADP can identify patients at low risk for a short-term major adverse cardiac event who may be suitable for early discharge; such an approach could be used to decrease the overall observation periods and admissions for chest pain and has the potential to affect health-service delivery worldwide.

© 2019 Brush JE Jr et al. JAMA Network Open.

Acute Coronary Syndrome

Differential Diagnoses

Diagnostic Considerations

As many as half of all cases of ACS are clinically silent in that they do not cause the classic symptoms of this syndrome. Consequently, ACS goes unrecognized by the patient. Maintain a high index of suspicion for ACS, especially when evaluating women, patients with diabetes, older patients, patients with dementia, and those with a history of heart failure.

Although ST-segment and T-wave changes are associated with CAD, alternative causes of these findings are left ventricular aneurysm, pericarditis, Prinzmetal angina, early repolarization, Wolff-Parkinson-White syndrome, and drug therapy (e.g., with tricyclic antidepressants, phenothiazines).

Increasing public awareness of the typical and atypical presentations of ACS is of the utmost importance for optimal and timely treatment. Many patients do not recognize that their symptoms are cardiac in origin and therefore may delay seeking medical help. Patients with established CAD call emergency medical services if they have chest pain that does not resolve after they take a sublingual nitroglycerin tablet.

In patients presenting to the ED with chest pain, a structured diagnostic approach that includes time-focused ED decision points, brief observation, and selective application of early outpatient provocative testing appeared both safe and diagnostically efficient in a study by Scheuermeyer et al. However, some patients with ACS may be discharged for outpatient stress testing on the index ED visit. [16]

© 2019 Brush JE Jr et al. JAMA Network Open.

Approach Considerations

Stable coronary artery disease (CAD) may result in ACS in the absence of plaque rupture and thrombosis, when physiologic stress (eg, trauma, blood loss, anemia, infection, tachyarrhythmias) increases demands on the heart. In such cases, the diagnosis of acute myocardial infarction can be made if workup reveals the typical rise and fall of biochemical markers of myocardial necrosis along with at least 1 of the following [2] :

- **Ischemic symptoms**
- **Development of pathologic Q waves on electrocardiogram (ECG)**
- **Significant ST-segment-T wave (ST-T) changes or new left bundle branch block (LBBB)**
- **Imaging evidence of new loss of viable myocardium or new regional wall motion abnormality**
- **Introcoronary thrombus identified by angiography or autopsy**

Non–ST-segment elevation myocardial infarction (NSTEMI) is distinguished from unstable angina by elevated levels of cardiac enzymes and biomarkers of myocyte necrosis. Differentiation is generally based on 3 sets of biomarkers measured at 6- to 8-hour intervals after the patient's presentation to the ED. The current definition of NSTEMI requires a typical clinical syndrome plus elevated troponin (or creatine kinase isoenzyme MB [CK-MB]) levels to over 99% of the normal reference (with a coefficient of variation of < 10% for the assay). Given this definition, nearly 25% of patients who were previously classified as having unstable angina now fulfill the criteria for NSTEMI.

Measure cardiac enzyme levels at regular intervals, starting at admission and continuing until the peak is reached or until 3 sets of results are negative. Biochemical biomarkers (demonstrated in the image below) are useful for diagnosis and prognostication.

© 2019 Brush JE Jr et al. JAMA Network Open.

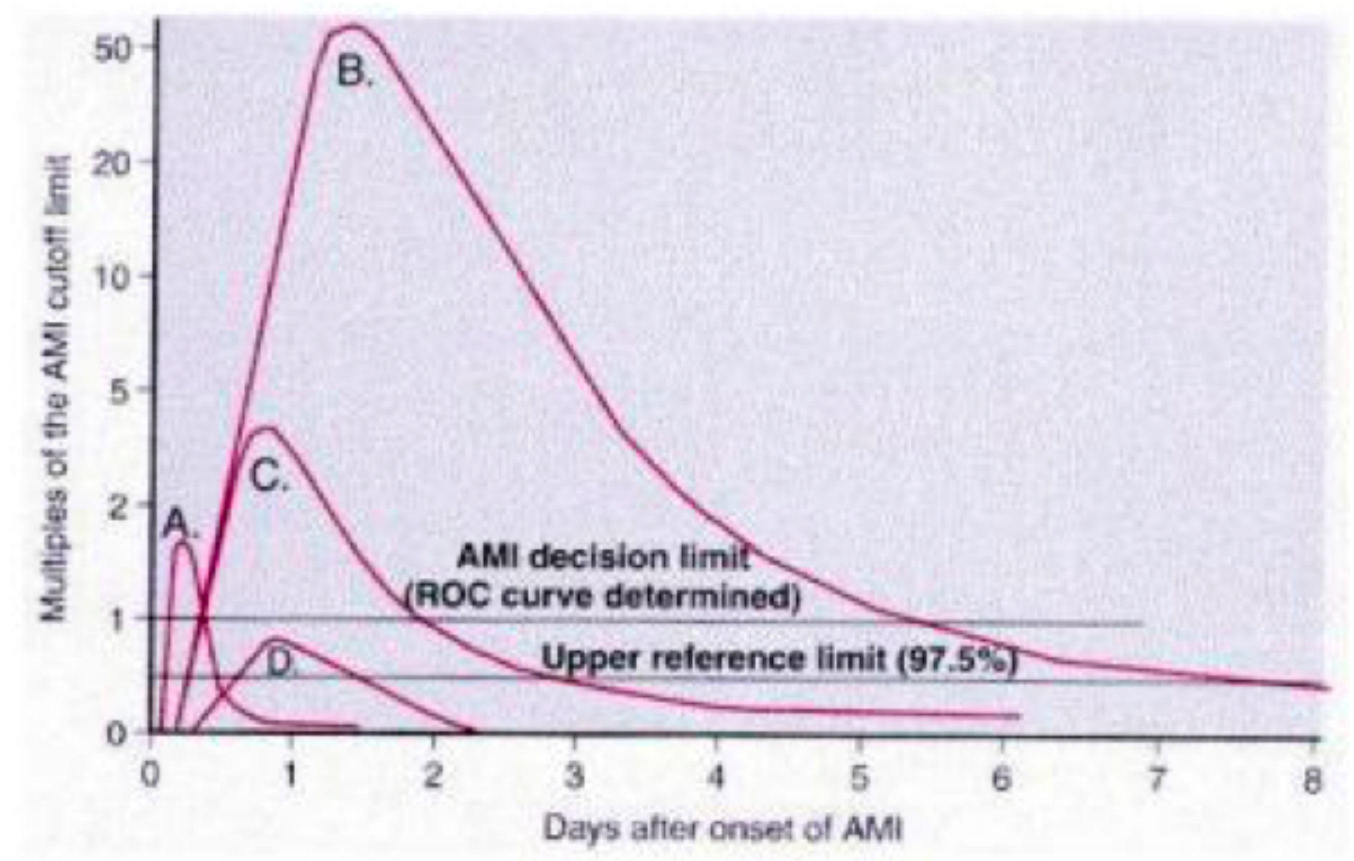

This plot shows changes in cardiac markers over time after the onset of symptoms. Peak A is the early release of myoglobin or creatine kinase isoenzyme MB (CK-MB) after acute myocardial infarction (AMI). Peak B is the cardiac troponin level after infarction. Peak C is the CK-MB level after infarction. Peak D is the cardiac troponin level after unstable angina. Data are plotted on a relative scale, where 1.0 is set at the myocardial-infarction cutoff concentration. Courtesy of Wu et al (1999). ROC = receiver operating characteristic.

Of note, cardiac-specific troponins are not detectable in the blood of healthy individuals; therefore, they provide high specificity for detecting injury to cardiac myocytes. These molecules are also more sensitive than CK-MB for myocardial necrosis and therefore improve early detection of small myocardial infarctions. Although blood troponin levels increase simultaneously with CK-MB levels (about 6 h after the onset of infarction), they remain elevated for as long as 2 weeks. As a result, troponin values cannot be used to diagnose reinfarction. New methods of detecting troponins in the blood can measure levels as low as 0.1-0.2 ng/mL.

Keller et al suggest that among patients with suspected acute coronary syndrome, highly sensitive troponin I assay (hsTnI) or contemporary troponin I assay (cTnI) determination 3 hours after admission for chest pain may facilitate early rule-out of acute myocardial infarction. A serial change in hsTnI or cTnI levels from admission (using the 99th percentile diagnostic cutoff value) to 3 hours postadmission may aid in early diagnosis of acute myocardial infarction. [17]

Minor elevations in these molecules can be detected in the blood of patients without ACS in the setting of myocarditis (pericarditis), sepsis, renal failure, acute congestive

© 2019 Brush JE Jr et al. JAMA Network Open.

heart failure (CHF), acute pulmonary embolism, or prolonged tachyarrhythmias.

© 2019 Brush JE Jr et al. JAMA Network Open.

Acute Coronary Syndrome Workup

Electrocardiography

ECGs should be reviewed promptly. Involve a cardiologist when in doubt.

Recording an ECG during an episode of the presenting symptoms is valuable. Transient ST-segment changes (>0.05 mV) that develop during a symptomatic period and that resolve when the symptoms do are strongly predictive of underlying CAD and have prognostic value. Comparison with previous ECGs is often helpful.

Alternative causes of ST-segment and T-wave changes are left ventricular aneurysm, pericarditis, Prinzmetal angina, early repolarization, Wolff-Parkinson-White syndrome, and drug therapy (e.g., with tricyclic antidepressants, phenothiazines).

In the emergency setting, ECG is the most important ED diagnostic test for angina. It may show changes during symptoms and in response to treatment, confirm a cardiac basis for symptoms. It also may demonstrate preexisting structural or ischemic heart disease (left ventricular hypertrophy, Q waves). A normal ECG or one that remains unchanged from the baseline does not exclude the possibility that chest pain is ischemic in origin. Changes that may be seen during anginal episodes include the following:

- **Transient ST-segment elevations**
- **Dynamic T-wave changes - Inversions, normalizations, or hyperacute changes**
- **ST depressions - May be junctional, downsloping, or horizontal**

In patients with transient ST-segment elevations, consider LV aneurysm, pericarditis, Prinzmetal angina, early repolarization, and Wolff-Parkinson-White syndrome as possible diagnoses. Fixed changes suggest acute myocardial infarction.

When deep T-wave inversions are present, consider the possibility of central nervous system (CNS) events or drug therapy with tricyclic antidepressants or phenothiazines as the cause.

Diagnostic sensitivity may be increased by performing right-sided leads (V_4 R), posterior leads (V_8, V_9), and serial recordings.

ECGs from 2 patients are shown below.

© 2019 Brush JE Jr et al. JAMA Network Open.

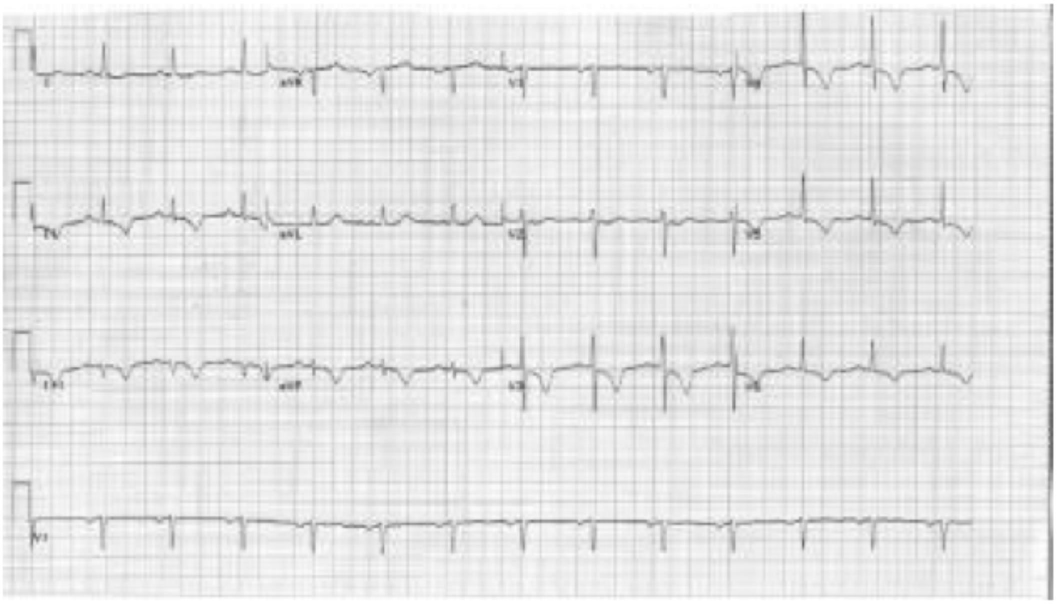

A 50-year-old man with type 1 diabetes mellitus and hypertension presents after experiencing 1 hour of midsternal chest pain that began after eating a large meal. Pain is now present but is minimal. Aspirin is the single drug that will have the greatest potential impact on subsequent morbidity. In the setting of ongoing symptoms and electrocardiogram (ECG) changes, nitrates titrated to 10% reduction in blood pressure and symptoms, beta blockers, and heparin are all indicated. If the patient continues to have persistent signs and/or symptoms of ischemia, addition of a glycoprotein IIb/IIIa inhibitor should be considered.

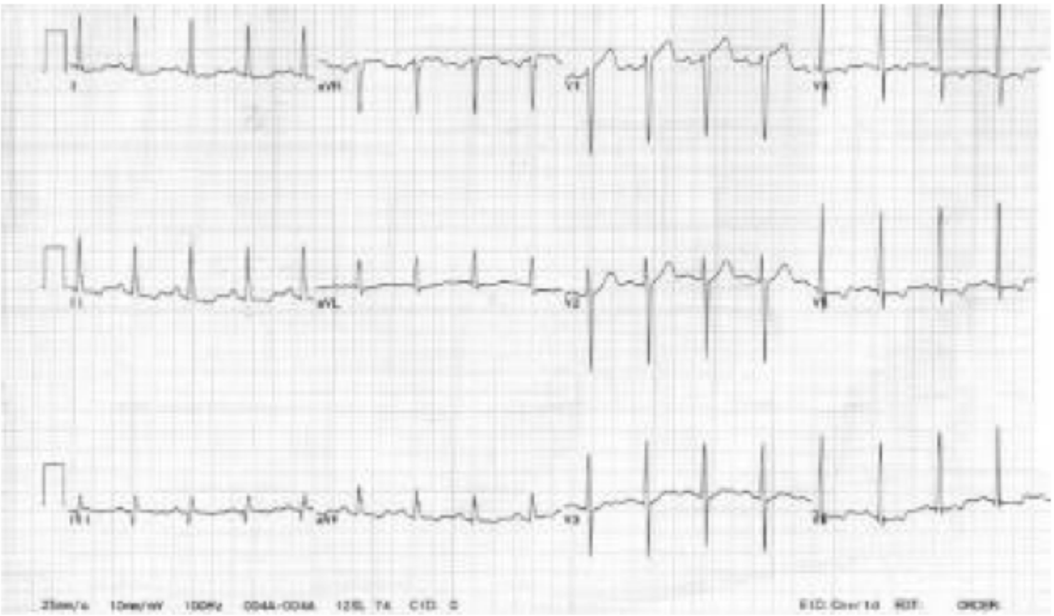

A 62-year-old woman with a history of chronic stable angina and a "valve problem" presents with new chest pain. She is symptomatic on arrival, complaining of shortness of breath and precordial chest tightness. Her initial vital signs are blood pressure = 140/90 mm Hg and heart rate = 98. Her electrocardiogram (ECG) is as

© 2019 Brush JE Jr et al. JAMA Network Open.

shown. She is given nitroglycerin sublingually, and her pressure decreases to 80/palpation. Right ventricular ischemia should be considered in this patient.

In difficult cases with nondiagnostic ECGs, such as those involving a left bundle-branch block, early imaging is useful to assess wall-motion abnormalities.

An important use of noninvasive imaging is to classify a patient has having NSTEMI or true STEMI.

The Optimal Cardiovascular Diagnostic Evaluation Enabling Faster Treatment of Myocardial Infarction (OCCULT-MI) trial compared the 80-lead (80L) mapping system to standard 12-lead (12L) ECG. The study concluded that the 80L body surface mapping technology detected more patients with MI or ACS than the 12L ECG, while still maintaining a high degree of specificity. [18]

A study by Damman et al examined information from 5,420 patients from the Fragmin and Fast Revascularization During Instability in Coronary Artery Disease (FRISC II), Invasive Versus Conservative Treatment in Unstable Coronary Syndromes (ICTUS), and Randomized Intervention Trial of Unstable Angina 3 (RITA-3) patient-pooled database. The study found that admission ECG characteristics had long-term prognostic value for cardiovascular death or myocardial infarction. Quantitative ECG characteristics showed no incremental discrimination compared with qualitative data. [19]

A 5-year follow-up of patients with non–ST-elevation acute coronary syndrome from these 3 trials found no link between a procedure-related MI and long-term cardiovascular mortality. However, long-term mortality substantially increased after a spontaneous MI. [20]

© 2019 Brush JE Jr et al. JAMA Network Open.

Acute Coronary Syndrome Workup

Measurement of Troponin levels

The troponins are regulatory proteins found in skeletal and cardiac muscle. The 3 subunits that have been identified include troponin I (TnI), troponin T (TnT), and troponin C (TnC). The genes that code for the skeletal and cardiac isoforms of TnC are identical; thus, no structural difference exists between them. However, the skeletal and cardiac subforms for TnI and TnT are distinct, and immunoassays have been designed to differentiate between them. This explains the cardiospecificity of the cardiac troponins. Skeletal TnI and TnT are structurally different. No cross-reactivity occurs between skeletal and cardiac TnI and TnT with the current assays.

The cardiac troponins are sensitive, cardiospecific, and provide prognostic information for patients with ACS. They have become the cardiac markers of choice for patients with ACS.

Early studies on the release kinetics of the cardiac troponins indicated that they were not early markers of myocardial necrosis. The early generation troponin assays yielded positive results within 4-8 hours after symptom onset, similar in timing to the release of CK-MB; however, they remained elevated for as long as 7-10 days post-myocardial infarction.

Initial studies on the cardiac troponins revealed a subset of patients with rest unstable angina in whom CK-MB levels were normal but who had elevated troponin levels. These patients had higher adverse cardiac event rates (acute myocardial infarction, death) within the 30 days after the index admission and a natural history that closely resembled patients with NSTEMI. The table below outlines many of the initial studies on troponins in ACS.

Trials of Troponins in ACS

		n	% positive troponin test	Event rate (death, AMI)	Follow up period
**Troponin T**					
Hamm	1992	64	40	33%	In hospital
Raviode	1995	127	35	22%	6 months
Wu	1995	131	21	30%	1month
Lindahl	1996	976	51	13%	1 month
Ohmann	1996	801	33	16%	1 month
Hamm	1997	315	22	22%	1 month
CAPTURE trials	1998	890	24	15%	1 month
Luscher	1997	516	48	11%	1 month
**Troponin I**					
Galvani	1996	91	24	23%	1month
Antman	1996	948	25	4%	6 weeks
Hamm	1997	315	36	19%	1 month
Luscher	1997	516	41	11%	1 month

© 2019 Brush JE Jr et al. JAMA Network Open.

Use of cardiac markers in the ED. Studies on troponins in ACS.

As previously mentioned, an elevated troponin level also enables risk stratification of patients with ACS and identifies patients at high risk for adverse cardiac events (i.e., myocardial infarction, death) up to 6 months after the index event. [5, 6]

In a study by Antman et al, the initial TnI level on admission in patients with ACS correlated with mortality at 6 weeks. CK-MB levels, although sensitive and specific for the diagnosis of acute myocardial infarction, were not predictive of adverse cardiac events and had no prognostic value. [5] The relationship between TnI levels and risk of cardiac events and mortality is demonstrated in the graphs below.

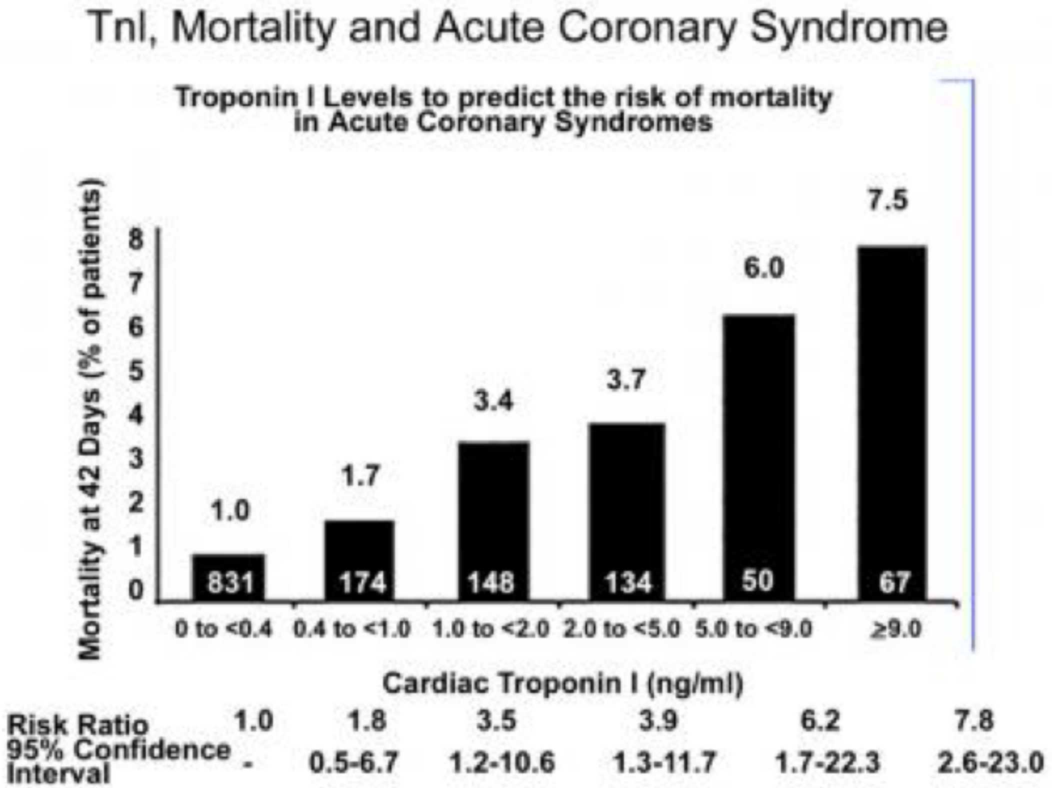

	0 to <0.4	0.4 to <1.0	1.0 to <2.0	2.0 to <5.0	5.0 to <9.0	≥9.0
Risk Ratio	1.0	1.8	3.5	3.9	6.2	7.8
95% Confidence Interval	-	0.5-6.7	1.2-10.6	1.3-11.7	1.7-22.3	2.6-23.0

Use of cardiac markers in the ED. Troponin I levels and cardiac mortality in ACS.

© 2019 Brush JE Jr et al. JAMA Network Open.

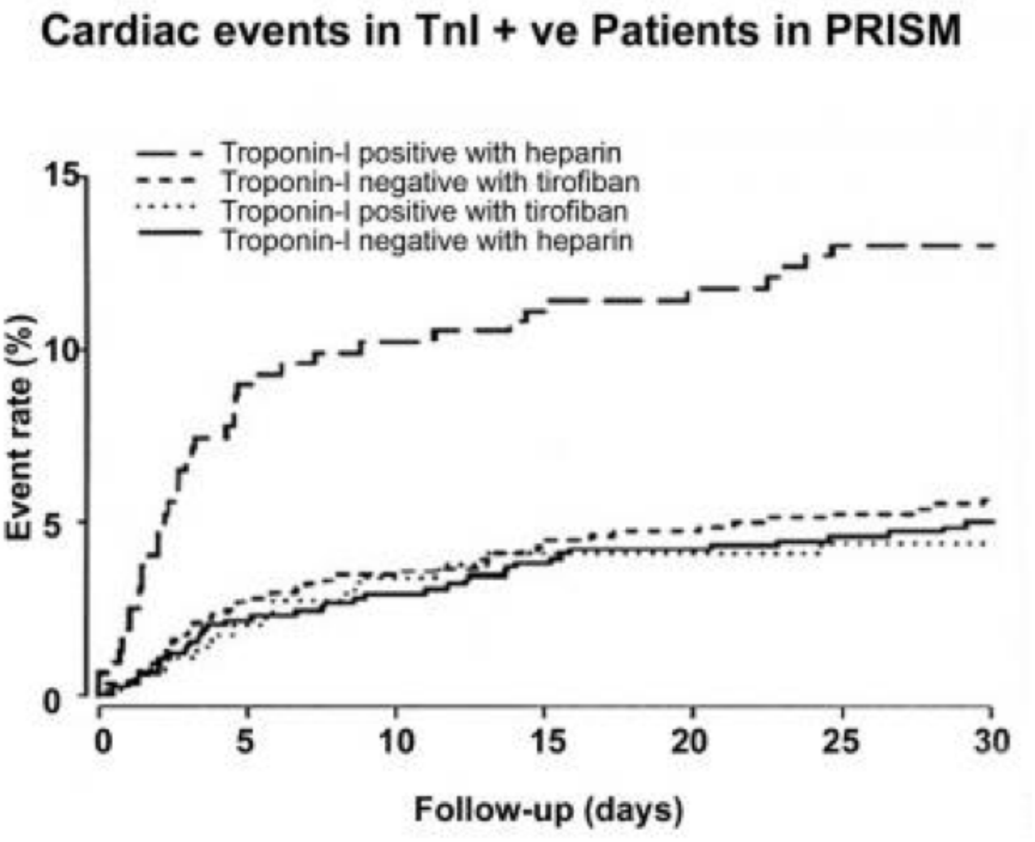

Use of cardiac markers in the ED. Cardiac event rates in the platelet receptor inhibition for ischemic syndrome (PRISM) study based on troponin I results.

Data from a meta-analysis indicated that an elevated troponin level in patients without ST-segment elevation is associated with a nearly 4-fold increase in cardiac mortality rate. For the composite end point of acute myocardial infarction or death, an elevated troponin level was associated with an odds ratio of 3.3. [21]

The TIMI IIIB, GUSTO IIa, GUSTO IV ACS, and Fragmin During Instability in Coronary Artery Disease (FRISC) trial all demonstrated a direct correlation between the level of TnI or TnT and the adverse cardiac event rate and mortality rate in ACS. [5, 22, 23, 24, 25] These studies confirmed the use of the cardiac troponins TnI and TnT in risk stratification and therapeutic decision making.

Studies by Ohman et al and Stubbs et al revealed that an elevated troponin level at baseline was an independent predictor of mortality even in patients with chest pain and acute myocardial infarction with ST-segment elevation who were eligible for reperfusion therapy. [22, 26]

With the introduction of increasingly sensitive and precise troponin assays, up to 80% of patients with acute myocardial infarction will be found to have an elevated troponin within 2-3 hours of ED arrival. With this improved clinical performance in cardiac troponin assays, the so-called rapidly rising cardiac biomarkers, such as myoglobin or CK-MB isoforms, have little clinical utility. [27, 28, 29, 30] In a prospective multicenter study, patients with ACS who and presented with acute chest pain to the ED were followed for 12 months. The study found that patients with

© 2019 Brush JE Jr et al. JAMA Network Open.

normal high-sensitivity cardiac troponin T (hs-cTnT) levels at presentation have low mortality rates but an increased rate of acute myocardial infarction during the subsequent 360 days. [31]

As a result, some authorities have called for a troponin standard alone and recommend eliminating CK-MB. [32]

Many patients with acute myocardial infarction present with equivocal ECG patterns, making prehospital ECG diagnosis difficult. A study by Sorensen et al suggests prehospital TnT testing may improve diagnosis in patients with chest pain transported by ambulance. [33] When quantitative TnT was measured at hospital arrival in 958 patients after 8 and 24 hours, a diagnosis of acute myocardial infarction was established in 208 of 258 patients with increased TnT levels, showing prehospital TnT testing is feasible with a high success rate. Prehospital implementation of quantitative tests, with lower detection limits, may identify most patients with acute myocardial infarction irrespective of ECG changes.

If myocardial injury is suspected despite negative cardiac-specific troponin findings, additional, sensitive laboratory assays are indicated. [34]

Patients with suspected ACS who test negative for troponin and copeptin can be safely discharged from the hospital without further testing, according to a recent study, the Biomarkers in Cardiology 8 (BiC-8) trial. Copeptin, a marker of severe hemodynamic stress, can be detected immediately in acute myocardial infarction. [35] The study involved 902 patients at low to intermediate risk of ACS; half of the patients were treated with standard care, and the other 451 patients underwent a copeptin assay. In the latter group, patients with a positive copeptin test, defined as a level of 10 pmol/L or greater, were treated with standard ACS care, while patients with a copeptin level below 10 pmol/L were discharged into ambulant care, including an outpatient visit within 72 hours. In the 451 patients tested for troponin and treated with standard care, the 30-day rate of major adverse cardiovascular events was 5.5%, compared with 5.46% in the 451 patients tested for troponin and copeptin (a statistically insignificant difference). [35]

© 2019 Brush JE Jr et al. JAMA Network Open.

Case 3

A 50-year-old woman presents to the ED with chest pain. She has a longstanding history of hypertension for which takes three medications. She also has a history of borderline diabetes. She ran out of her blood pressure medications 3 days ago and hasn't been able to get into see her primary care doctor to get a renewal for her prescriptions. Yesterday, she began having chest pain described as a pressure in the mid chest without radiation. The pain occurred off and on throughout the day. She was relatively inactive yesterday and the pain did not seem to worsen with activity. She felt mildly short of breath along with the pain. She decided to come to the ED today because the pain was persisting, and also to see if she could get a refill on her BP medications. She is complaining of 3/10 mid sub-sternal chest tightness.

PMH is remarkable for a 5-year history of hypertension and borderline diabetes controlled with diet. She normally takes dyazide, metoprolol, and amlodipine for hypertension.

She is married and works as a waitress. She quit smoking 3 years ago and drinks occasionally.

On examination, HR=100, BP=200/110, RR=28. She is obese in mild distress due to pain. HEENT is unremarkable, Lungs are clear, Cardiac exam reveals a regular rate and rhythm and no murmurs. The abdomen is obese and benign.

On a scale of 0 (very IMprobable) to 100 (very PROBABLE), what is the probability that this patient has an acute myocardial infarction? *

Each answer must be between 0 and 100
Only an integer value may be entered in this field.

Please write your answer here:

Your initial probability answer was: {INSERTANS:485846X3481X27655}

A troponin level was drawn. The value is less than 30, which is reported as normal.

Now what is the probability that this patient has an acute myocardial infarction? *

Each answer must be between 0 and 100
Only an integer value may be entered in this field.

Please write your answer here:

Experts disagree what the diagnosis is at this point; *it could either be a hypertensive emergency or an acute myocardial infarction.*

© 2019 Brush JE Jr et al. JAMA Network Open.

Submit your survey.
Thank you for completing this survey.

© 2019 Brush JE Jr et al. JAMA Network Open.

Concept Group

1. Consent
2. Instructions
3. Link to video and 3 cases

© 2019 Brush JE Jr et al. JAMA Network Open.

EBM Study V2

There are 12 questions in this survey

Please review the following consent form.

© 2019 Brush JE Jr et al. JAMA Network Open.

Questionnaire Consent Form

Eastern Virginia Medical School (EVMS) Institutional Review Board

Study Title: ***A Study to Evaluate Strategies for Teaching Effective Use of Diagnostic Tests (EBM)***

Name of Investigator: John Brush, MD

Sponsor: None

You are being asked to participate in a research study involving the collection of information in the form of questionnaire. The purpose of the research project is to examine the effective use of diagnostic tests in clinical reasoning among pre-clinical medical students.

The study involves an online 90-minute session. You will receive standard instructions about how to use diagnostic tests, then either a) more detailed conceptual instruction about the relation between diagnostic test characteristics and disease probability, or b) a series of systematically chosen cases showing how test characteristics can influence probability of disease or c) a control condition where you will learn more about the specific conditions used in the study. You will then be asked to interpret 20 cases by providing a diagnosis and probability before and after receiving the results of the test.

You will reimbursed $30 for your participation. There are no additional costs to you associated with taking part in this study.

A risk associated with allowing your data to be saved is the release of personal information from your study record. We will strive to protect your records so that your personal information (like name, address, social security number and phone number) will remain private. There also may be other risks that are unknown and we cannot predict.. Your academic standing will not be effected by participation, or choosing not to participate. Responses to study questions will not become part of your academic record.

Although the results of this research may not benefit you directly, they may be made available upon request. You have the potential to receive educational benefit from participation. Your participation will contribute to our understanding of this skill, and may benefit future trainees. However, participation will not provide any benefit to your academic standing or increased merit compared to students not participating.

In conducting this research study, the data from the study will be analyzed statistically to test study hypotheses, and will be retained in an anonymized form for 10 years. No individual identifiers will be present in the database. Your protected health information (PHI), which includes personal information about you only be collected.

Your study records may be reviewed and/or copied in order to meet state and/or federal regulations. Reviewers may include, for example, Eastern Virginia Medical School

© 2019 Brush JE Jr et al. JAMA Network Open.

Institutional Review Board, study investigator, John Brush, MD and his research team, and the research collaborators at McMaster University.

Information learned from this research may be used in reports, presentations and publications. None of these will personally identify you.

Taking part in this study is your choice. If you decide not to take part, your choice will not affect any medical benefits to which you are entitled. You may choose to leave the study at any time by notifying the study investigator. The study investigator may decide to take you off this study if you revoke your authorization.

We will tell you about new information that may affect your health, welfare, or willingness to stay in this study.

In the event of injury resulting from this research study, Eastern Virginia Medical School (EVMS) provides no financial compensation plan or free medical care.

If you have any questions pertaining to this research you may contact John Brush, MD or Judith Taylor-Fishwick at DiagnosticReasoningStudy@evms.edu, 757-446-8475. If you believe you have suffered an injury as a result of your participation in this study, you should contact the principal investigator, John Brush, MD at 757-446-8475. You may also contact Betsy Conner, director, EVMS Human Subjects Protection Program and IRB office at (757) 446-5854. If you have any questions pertaining to your rights as a research subject, you may contact a member of the Institutional Review Board through the Institutional Review Board office at (757) 446-8423.

You are being asked to participate in the above research study, which is being conducted at Eastern Virginia Medical School (EVMS), where you are an employee or student. The research study has been described to you, in writing, on this electronic consent form. You have also had the opportunity to ask the investigators conducting this study any questions that you may have regarding participation in this study.

The purpose of this consent form is to inform you that you have the right to choose not to participate in this research study. If you choose not to participate, or to withdraw at any time, it will not affect your standing as an employee or student.

If you are an employee, your participation will not place you in good favor with the investigator, your supervisor, or EVMS (e.g., increase in salary, promotion, extra vacation, or the like). Not participating will not adversely affect your employment with EVMS, in particular the position that you currently hold. If you are a student, your participation will not place you in good favor with the investigator or other faculty (e.g., receiving better grades, recommendations, employment). Also, not participating in this study will not adversely affect your relationship with the investigator or other faculty.

By clicking "Agree", you are consenting to this study. You are able to print out a copy of this consent form for your records.

Note: Do NOT click on the "BACK" button in your browser while completing this study.

© 2019 Brush JE Jr et al. JAMA Network Open.

*

Please choose **all** that apply:

☐ **Agree**

If you do NOT agree, you can exit this study by closing your browser or clicking on the "exit and clear survey" button below.

© 2019 Brush JE Jr et al. JAMA Network Open.

Instruction Video

In this session, you will watch a 20-minute video on essential evidence-based medicine (EBM) principles, including how to calculate and use likelihood ratios.

After the video, you will view three clinical cases to practice interpreting diagnostic tests.

YOU MUST FINISH THIS SESSION IN ONE SITTING. If you don't have approximately 30 minutes at the moment, please click on the "Exit and clear survey" button at the bottom of this page and come back to it later using the same link.

When the video ends, please return to this screen and click on the NEXT button below. This will take you to the next screen and the three clinical cases.

In 24 hours, you will receive an invitation to participate in the second session.

The second session will be available for 72 hours. You will be asked to interpret 20 cases by providing a diagnosis and probability before and after receiving the results of a diagnostic test and likelihood ratios. The second session will take about 30 minutes.

Please click on the link below to view a 20-minute instruction video.

https://youtu.be/pIq0XaYwCm8

IF FOR SOME REASON, YOU CANNOT CLICK ON THE "NEXT" BUTTON AFTER VIEWING THE VIDEO (IF FOR INSTANCE THE BROWSER CLOSES WHILE WATCHING THE VIDEO), PLEASE REOPEN THE ORIGINAL LINK IN YOUR EMAIL, SKIP THE VIDEO (JUST CLICK "NEXT") AND CONTINUE TO THE THREE CASES.

Thank you.

© 2019 Brush JE Jr et al. JAMA Network Open.

Clinical Case Instructions

For each of the following 3 cases, after clinical information is presented, you will be asked for a probability (1-100) of the diagnosis. Then, a test result will be given and you will be asked for a post-test probability of the diagnosis.

© 2019 Brush JE Jr et al. JAMA Network Open.

Case 1 of 3

An 80 year-old woman is presents to the ED with acute shortness of breath. She has had several myocardial infarctions in the past, has a history of chronic systolic heart failure and presents acutely short of breath. She lives alone and usually her daughter brings her meals to her. Over the past week, however, her daughter has been out of town and the family arranged for a neighbor to prepare the patient's meals. The neighbor supplied canned vegetables and soups and the patient noticed that the food was more salty than she was used to. She has been quite thirsty and increased her fluid intake over the past week. The day prior to admission she became more short of breath and she was unable to sleep last night due to difficulty breathing. She noticed that her ankles and hands have become swollen recently. She denies chest pain or palpitations.

PMH is remarkable for a history of coronary artery disease, hypertension, and chronic systolic heart failure. Her medications are carvedilol, lisinipril, and furosemide, which she has been taking regularly.

The patient is widowed. She formerly worked as a hairdresser. She does not smoke or drink alcohol.

On exam, HR=105, BP=100/60, RR=32. She is elderly, frail, and acutely short of breath. HEENT reveals bluish lips. Neck exam reveals jugular venous distension. Lungs reveal wet crackles throughout. She has a tachycardic heart rhythm and an S3 gallop. The abdomen is slightly distended and benign. There is 1+ bilateral pitting edema.

On a scale of 0 (very IMprobable) to 100 (very PROBABLE), what is the probability that this patient has congestive heart failure? *

Each answer must be between 0 and 100
Only an integer value may be entered in this field.

Please write your answer here:

© 2019 Brush JE Jr et al. JAMA Network Open.

Your initial probability answer was: {INSERTANS:793717X3470X27622}

A chest x-ray is performed. The radiologist interprets the x-ray as positive for pulmonary venous congestion.

Studies show that a chest x-ray showing pulmonary venous congestion is predictive of congestive heart failure with a sensitivity of 54%, specificity of 96%, and a positive likelihood ratio of 13.5 and a negative likelihood ratio of 0.5.

Now what is the probability that the patient has congestive heart failure? *

Each answer must be between 0 and 100
Only an integer value may be entered in this field.

Please write your answer here:

Experts indicate that the correct answer for this case is: ***congestive heart failure***

© 2019 Brush JE Jr et al. JAMA Network Open.

Case 2 of 3

A 29-year-old woman presents complaining of chest heaviness. For the past three days she has complained of sinus congestion, a sore throat and a dry non-productive cough. She is worried that she might have caught something from her roommate, who has also been sick for the past few days. However, her inability to catch her breath is the reason that she is attending the emergency department. She denies any pleuritic chest pain or calf swelling. She was able to exercise yesterday without feeling short of breath. However, she kept coughing, so she gave up when people at the gym kept giving her a dirty look.

The patient is well without any other medical problems. She is a non-smoker. Her only medication is ibuprofen, which she has been taking for the last 3 days to address her sore throat and because “her body feels achy and run down.”

On examination, her temperature is 37.3C; pulse 76 / minute; blood pressure 110/76mmHg; and respiratory rate 18 / minute. Her heart rate is regular. She has normal pulses. There are no extra cardiac sounds or murmurs. Her chest exam is normal with an occasional transmitted upper respiratory sound. She has an inflamed oropharynx with without evidence of purulence. Her calves are symmetric, non-tender and not swollen.

On a scale of 0 (very IMprobable) to 100 (very PROBABLE), what is the probability that this patient has a pulmonary embolism? *

Each answer must be between 0 and 100
Only an integer value may be entered in this field.

Please write your answer here:

© 2019 Brush JE Jr et al. JAMA Network Open.

Your initial probability answer was: {INSERTANS:793717X3471X27625}

A quantitative D-dimer is measured. The value is less than 500, which is reported as negative.

Studies show that a quantitative d-dimer is predictive of pulmonary embolism with a sensitivity of 96% and a specificity of 45% with a positive likelihood ratio of 1.7 and a negative likelihood ratio of 0.09.

Now what is the probability that this patient has a pulmonary embolism? *

Each answer must be between 0 and 100
Only an integer value may be entered in this field.

Please write your answer here:

Experts indicate that the correct answer for this case is: ***an upper respiratory infection (URI).***

© 2019 Brush JE Jr et al. JAMA Network Open.

Case 3 of 3

A 50-year-old woman presents to the ED with chest pain. She has a longstanding history of hypertension for which takes three medications. She also has a history of borderline diabetes. She ran out of her blood pressure medications 3 days ago and hasn't been able to get into see her primary care doctor to get a renewal for her prescriptions. Yesterday, she began having chest pain described as a pressure in the mid chest without radiation. The pain occurred off and on throughout the day. She was relatively inactive yesterday and the pain did not seem to worsen with activity. She felt mildly short of breath along with the pain. She decided to come to the ED today because the pain was persisting, and also to see if she could get a refill on her BP medications. She is complaining of 3/10 mid sub-sternal chest tightness.

PMH is remarkable for a 5-year history of hypertension and borderline diabetes controlled with diet. She normally takes dyazide, metoprolol, and amlodipine for hypertension.

She is married and works as a waitress. She quit smoking 3 years ago and drinks occasionally.

On examination, HR=100, BP=200/110, RR=28. She is obese in mild distress due to pain. HEENT is unremarkable, Lungs are clear, Cardiac exam reveals a regular rate and rhythm and no murmurs. The abdomen is obese and benign.

On a scale of 0 (very IMprobable) to 100 (very PROBABLE), what is the probability that this patient has an acute myocardial infarction? *

Each answer must be between 0 and 100
Only an integer value may be entered in this field.

Please write your answer here:

© 2019 Brush JE Jr et al. JAMA Network Open.

Your initial probability answer was: {INSERTANS:793717X3472X27628}

A troponin level was drawn. The value is less than 30, which is reported as normal.

Studies show that troponin testing in this setting has a sensitivity of 95%, specificity of 80% and a positive likelihood ratio of 4.75 and a negative likelihood ratio of 0.1.

Now what is the probability that this patient has an acute myocardial infarction? *

Each answer must be between 0 and 100
Only an integer value may be entered in this field.

Please write your answer here:

Experts disagree what the diagnosis is at this point; *it could either be a hypertensive emergency or an acute myocardial infarction.*

© 2019 Brush JE Jr et al. JAMA Network Open.

Submit your survey.
Thank you for completing this survey.

© 2019 Brush JE Jr et al. JAMA Network Open.

Test for Experience and Control Groups

1. Instructions
2. Test questions

© 2019 Brush JE Jr et al. JAMA Network Open.

EBM T1 test for reading control and experiential conditions

There are 45 questions in this survey

Instructions

Welcome back! We very much appreciate your participation in this study.

In this session, you will be presented with a total of 20 cases.

As in the first session, you will be asked for a probability for the diagnosis for each case, after clinical information is presented. Then, a test result will be given and you will be asked for a post-test probability.

YOU MUST COMPLETE ALL OF THE CASES IN ONE SITTING. If you don't have approximately 30 minutes at the moment, please click on the "Exit and clear survey" button at the bottom of this page and come back to it later using the same link BUT remember that the survey expires 72 hours after you received the email with the link for this session.

AFTER you complete the test you will be directed to a separate survey to complete information regarding the HONORARIUM. A new page will be opened so that we cannot link your answers to your contact information.

Thanks.

© 2019 Brush JE Jr et al. JAMA Network Open.

Session 2 - Case 1 of 20

A 28 year-old woman presents to the ED with a 3-day history of shortness of breath. She also complains of a dry cough and sore throat. Over the past 3 days, she has had mild chills but hasn't taken her temperature. For the last 3 days she has shortness of breath when she climbs the stairs of her apartment building carrying groceries. She sleeps flat at night and denies ankle edema.

Her past medical history is unremarkable. She denies hypertension and only takes birth control pills. She has no known allergies.

On examination, she is afebrile, HR=98, BP=120/80. RR=20. Her throat is slightly erythematous and she has mildly enlarged cervical lymph nodes. Lungs are clear to auscultation and her heart exam is normal.

On a scale of 0 (very IMprobable) to 100 (very PROBABLE), what is the probability that this patient has congestive heart failure? *

Each answer must be between 0 and 100
Only an integer value may be entered in this field.

Please write your answer here:

Your initial probability answer was: {INSERTANS:251728X3331X27193}

A chest x-ray is performed. The radiologist interprets the x-ray as negative for pulmonary venous congestion.

Now what is the probability that the patient has congestive heart failure?

*

Each answer must be between 0 and 100
Only an integer value may be entered in this field.

Please write your answer here:

© 2019 Brush JE Jr et al. JAMA Network Open.

Session 2 - Case 2 of 20

A 50-year-old African American woman presents to the ED with progressive shortness of breath. She has longstanding hypertension, obesity, and type 2 diabetes mellitus. Over the years, she developed progressive renal failure and one year ago was placed on hemodialysis. Over the past two weeks she has developed increasing dyspnea on exertion. Over the last three days she has developed sinus congestion, myalgia and a dry cough. She now has to go slowly when she climbs the stairs up to her second-story apartment. She has always slept on 2 pillows. She has noticed some increasing ankle edema lately and finds that her hands appear a bit puffy making her wedding ring feel tight.

PMH-hypertension, diabetes, end-stage renal disease on dialysis. Her medications are losartan, amlodipine, glyburide, and calcitriol.

The patient lives locally with her husband and is no longer working. She continues to smoke ½ ppd and drinks an occasional beer.

On exam, she is afebrile, HR-80, BP-170/80, RR=28. She is obese and in mild respiratory distress. There is minimal jugular venous distension. Lung exam reveals occasional scattered wheeze and cardiac exam is unremarkable except for bilateral 2+ ankle edema.

On a scale of 0 (very IMprobable) to 100 (very PROBABLE), what is the probability that this patient has congestive heart failure? *

Each answer must be between 0 and 100
Only an integer value may be entered in this field.

Please write your answer here:

© 2019 Brush JE Jr et al. JAMA Network Open.

Your initial probability answer was:
{INSERTANS:251728X3334X27201}

A chest x-ray is performed. The radiologist interprets the x-ray as negative for pulmonary venous congestion.

Now what is the probability that the patient has congestive heart failure?

*

Each answer must be between 0 and 100
Only an integer value may be entered in this field.

Please write your answer here:

© 2019 Brush JE Jr et al. JAMA Network Open.

Session 2 - Case 3 of 20

A 60-year-old man presents to the ED with progressive shortness of breath. The patient lives in a nearby homeless shelter, is currently unemployed, and has a long-standing history of alcohol abuse. He continues to drink about a pint of alcohol daily. He was transferred from the homeless shelter because of severe shortness of breath and acute intoxication. He is a difficult historian but does give a history of severe shortness of breath with minimal exertion over the past month. He has trouble sleeping at night and has found that he can get some sleep if he sits up in a chair or leans over a table while sleeping. He has notices that his abdomen has become distended and he has swelling in the ankles. He denies chest pain, palpitations, or lightheadedness. He denies cough or fever.

Past medical history is remarkable for a history of hospitalizations in the past for acute alcohol intoxication, but otherwise, he has avoided contact with doctors and has no other reported past medical problems. He takes no medications.

He lives at the homeless shelter. He is divorced and unemployed, formerly working as a school custodian. He smokes a few cigarettes daily and drinks a pint of vodka daily.

On exam, HR=115, BP=90/60, RR=28. He is disheveled and smells of alcohol and appears intoxicated and acutely short of breath. He has jugular venous distention, bibasilar rales, an S3 gallop, and 3+ bilateral pitting edema. Abdominal exam reveals an enlarged liver and possible ascites.

On a scale of 0 (very IMprobable) to 100 (very PROBABLE), what is the probability that this patient has congestive heart failure? *

Each answer must be between 0 and 100
Only an integer value may be entered in this field.

Please write your answer here:

© 2019 Brush JE Jr et al. JAMA Network Open.

Your initial probability answer was: {INSERTANS:251728X3335X27204}

A chest x-ray is performed. The radiologist interprets the x-ray as positive for pulmonary venous congestion.

Now what is the probability that the patient has congestive heart failure?

*

Each answer must be between 0 and 100
Only an integer value may be entered in this field.

Please write your answer here:

© 2019 Brush JE Jr et al. JAMA Network Open.

Session 2 - Case 4 of 20

A 70 year-old man presents to the ED with progressive shortness of breath. He had an inferior ST elevation myocardial infarction 5 years ago treated with emergency stenting of the right coronary artery. He is obese and has a history of obstructive sleep apnea. He uses CPAP at night. Over the past month and a half, he has noticed a marked increase in his shortness of breath. He is now short of breath when he plays golf and does routine things around the house. He sleeps flat at night using CPAP and has had mild ankle edema.

PMH is remarkable for CAD and obstructive sleep apnea, as well as hypertension and type-2 diabetes mellitus.

He lives with his wife. He quit smoking when he had his heart attack 5 years ago and drinks one mixed drink each night.

On exam, he is afebrile, HR=100, BP=150/80, RR=18. His neck is obese and the jugular veins are not visualized. Lungs reveals fine bibasilar rales. Cardiac exam reveals no extra heart sounds. He has 1+ ankle edema.

On a scale of 0 (very IMprobable) to 100 (very PROBABLE), what is the probability that this patient has congestive heart failure? *

Each answer must be between 0 and 100
Only an integer value may be entered in this field.

Please write your answer here:

© 2019 Brush JE Jr et al. JAMA Network Open.

Your initial probability answer was: {INSERTANS:251728X3336X27207}

A chest x-ray is performed. The radiologist interprets the x-ray as negative for pulmonary venous congestion.

Now what is the probability that the patient has congestive heart failure?

*

Each answer must be between 0 and 100
Only an integer value may be entered in this field.

Please write your answer here:

© 2019 Brush JE Jr et al. JAMA Network Open.

Session 2 - Case 5 of 20

A 28-year-old woman arrives in the emergency department with her roommate for acute onset flank pain. She says the pain is a constant dull ache; however, it regularly increases in intensity to become nearly unbearable before dying back down. The pain radiates into her groin. For the past three days she has fell unwell. The patient also notes discomfort with urination. Today she has noticed gross hematuria when she urinated. She has never had this pain before.

The patient has a history of urinary tract infections. She also has adult ADHD (attention deficit hyperactivity disorder) for which she self-medicates with marijuana. She is a graduate student who has taken a leave of absence to train for the national triathlon team.

On physical examination she appears uncomfortable. Occasionally, she is unable to lay still, but she will then settle complaining of a dull pain. Her temperature is 37.2C; pulse 105/minute; blood pressure 150/95mmHg; respiratory rate 20/minute. Her chest is clear to auscultation and heart sounds are normal but with a regular tachycardic rate. Her abdomen is non-tender and soft. She has left costovertebral angle tenderness to percussion.

On a scale of 0 (very IMprobable) to 100 (very PROBABLE), what is the probability that this patient has pyelonephritis? *

Each answer must be between 0 and 100
Only an integer value may be entered in this field.

Please write your answer here:

Your initial probability answer was:
{INSERTANS:251728X3337X27210}

A urinalysis is performed. It demonstrates 3+RBC, 1+WBC, dipstick urinalysis was positive for nitrates and leukocyte esterase.

Now what is the probability that the patient has pyelonephritis?

*****

Each answer must be between 0 and 100
Only an integer value may be entered in this field.

Please write your answer here:

© 2019 Brush JE Jr et al. JAMA Network Open.

Session 2 - Case 6 of 20

A 75 yo woman presents to the emergency department for acute onset right knee pain and swelling that awoke her from her sleep. She denies trauma to the limb, although she recalls having a heavy box fall from a shelf and land on her leg yesterday. She walks with considerable pain but is able to navigate the waiting room of the emergency department to her exam room. There is no history of fevers or chills. She denies any recent camping or tick bites. She has not noted any rashes.

Her past medical history includes podagara (gout of the first metatarsal phalynx joint) and diabetes mellitus.

She is currently taking glyburide for her diabetes and has no known allergies.

On physical examination, she is nontoxic appearing. Her temperature is 37.5 C; pulse 86 / minute; blood pressure 125/70 mmHg; respiratory rate 16 / minute. Her cardiac exam is normal. Her right knee is slightly red and warm. There is an obvious effusion. While there is pain with movement, she is able to fully flex and extend the knee, although end of range is restricted by the effusion.

On a scale of 0 (very IMprobable) to 100 (very PROBABLE), what is the probability that this patient has crystal arthropathy? *

Each answer must be between 0 and 100
Only an integer value may be entered in this field.

Please write your answer here:

© 2019 Brush JE Jr et al. JAMA Network Open.

Your initial probability answer was:
{INSERTANS:251728X3338X27213}

An arthrocentesis is performed. It demonstrates 50,0000 WBC /ml and negatively birefringent crystals.

Now what is the probability that the patient has crystal arthropathy?

*****

Each answer must be between 0 and 100
Only an integer value may be entered in this field.

Please write your answer here:

[ ]

© 2019 Brush JE Jr et al. JAMA Network Open.

Session 2 - Case 7 of 20

A 37-year-old male presents to the emergency department for evaluation of five days of a malaise with cough. He felt like he has had a fever though he does not have a thermometer at home. He is coming to the emergency department because he is now experiencing sharp anterior chest pain. The pain is worse with inspiration. He also notes that if he bends forward the pain is minimal, but if he lies flat on his back, the pain is intense. He complains of shortness of breath on exertion

His past medical history is remarkable for hypertension, for which he takes atenolol. He has no allergies. He drinks alcohol rarely, and smokes ½ ppd, though does not use any illicit drugs.

On examination, his temperature was 37.9, pulse 96, BP 150/96, and RR 15. In general, he appears tired but non-toxic, and is speaking full sentences. He has dry mucous membranes; his oropharynx has no erythema or exudate, and he has no anterior or posterior chain cervical lymphadenopathy. On chest exam, he has crackles at the right base. His heart has a regular rate and rhythm. The cardiac exam is normal. Examination of his extremities demonstrates a tender calf on the right but not obvious swelling. His skin demonstrates no rashes, petechiae, or purpura.

On a scale of 0 (very IMprobable) to 100 (very PROBABLE), what is the probability that this patient has a pulmonary embolism? *

Each answer must be between 0 and 100
Only an integer value may be entered in this field.

Please write your answer here:

© 2019 Brush JE Jr et al. JAMA Network Open.

Your initial probability answer was:
{INSERTANS:251728X3339X27216}

A quantitative D-dimer is measured. The value is greater than 500, which is reported as positive.

Now what is the probability that the patient has a pulmonary embolism?

*

Each answer must be between 0 and 100
Only an integer value may be entered in this field.

Please write your answer here:

© 2019 Brush JE Jr et al. JAMA Network Open.

Session 2 - Case 8 of 20

A 29 yo female presents complaining of pleuritic chest pain. The pain has been waxing and waning for the last 24 hours. The patient does not recall any precipitating factors, specifically trauma or upper respiratory symptoms. When the pain did not resolve on its own she sought medical attention. She noted that although she has no cough, when she walked up the stairs from the parking garage she was breathless at the top. This is unusual for her as she is active and in shape. Her biggest complaint is that she can only take tiny breaths, any deep inspiration intensifies the sharp chest pain across her right anterior chest.

Her past medical history is unremarkable. She has no history of cardiac or pulmonary disease. Her only medication is an oral contraceptive pill. She still smokes, mostly on the weekends in social situations, but is actively reducing the amount she smokers per day.

On examination, her temperature is 36.9, pulse 135, BP 107/80, and RR 26. Her chest is clear to auscultation. There is no tenderness on palpation. Her cardiac exam is unremarkable. Her right calf is obviously swollen in comparison to her other legs. On palpation of the right calf she notes that it is slightly tender, a symptom she had not noticed prior to the exam.

On a scale of 0 (very IMprobable) to 100 (very PROBABLE), what is the probability that this patient has a pulmonary embolism? *

Each answer must be between 0 and 100
Only an integer value may be entered in this field.

Please write your answer here:

© 2019 Brush JE Jr et al. JAMA Network Open.

Your initial probability answer was:
{INSERTANS:251728X3340X27219}

A quantitative D-dimer is measured. The value is greater than 500, which is reported as positive.

Now what is the probability that the patient has a pulmonary embolism?

*

Each answer must be between 0 and 100
Only an integer value may be entered in this field.

Please write your answer here:

© 2019 Brush JE Jr et al. JAMA Network Open.

Session 2 - Case 9 of 20

A 53-year-old female presents to the emergency department because of shortness of breath. The dyspnea occurred suddenly and awoke the patient from sleep. The patient also reports that she is having retrosternal chest pain, which is worse on deep breathing. The patient reports that she has been feeling generally tired for about 4 days, with a sinus congestion as well as fevers and chills. She has also had a cough for several days and had coughed up yellow-green sputum with occasional flecks of blood. The patient also reports that he is having nausea. She vomited a small amount of bile during the triage interview. She has had no recent surgery. She recently returned from a trip to Bali and complains of the lack of space in an economy seat.

Her past medical history is remarkable for a tubal ligation 13 years ago and pneumonia two years ago. She takes no medications and has no allergies. She smoked previously but stopped 8 years ago when she became pregnant with her first child.

On examination, her temperature is 38.0, pulse 116, BP 110/96, and RR 20. On auscultation of her chest, she has no adventitial breath sounds. Her heart is regular rate and rhythm, with no murmurs, gallops, or rubs. There was trace pitting edema in both of her ankles.

On a scale of 0 (very IMprobable) to 100 (very PROBABLE), what is the probability that this patient has a pulmonary embolism? *

Each answer must be between 0 and 100
Only an integer value may be entered in this field.

Please write your answer here:

© 2019 Brush JE Jr et al. JAMA Network Open.

Your initial probability answer was: {INSERTANS:251728X3341X27222}

A quantitative D-dimer is measured. The value is less than 500, which is reported as negative.

Now what is the probability that the patient has a pulmonary embolism?

*

Each answer must be between 0 and 100
Only an integer value may be entered in this field.

Please write your answer here:

© 2019 Brush JE Jr et al. JAMA Network Open.

Session 2 - Case 10 of 20

A 45-year-old female presents with a 6 day history of difficulty breathing. "It feels as though I'm breathing through a wet towel." The patient denies any chest pain. She has been using a Ventolin inhaler without significant relief for the past three days. She indicates that she has been diagnosed with asthma by her family physician without formal testing; however, her condition is so mild that she infrequently (less than once a year) requires bronchodilators. "I wonder if my puffer is useless, I got it last year and haven't touched it since."

Past medical history is only significant for asthma. The patient is a smoker but has cut down to less than a quarter package of cigarettes per day. She takes a multivitamin and a vitamin D tablet daily.

On examination, her temperature is 36.9C; pulse 106 / minute; blood pressure 100/70mmHg; and respiratory rate 22 / minute. She has equal strong pulses in all limbs. Her cardiac exam reveals no rubs, or murmurs. She has faint scattered wheezes in all lung fields. She can speak in full sentences, although she is occasionally interrupted by the need to cough and clear her throat. She has no calf tenderness or swelling.

On a scale of 0 (very IMprobable) to 100 (very PROBABLE), what is the probability that this patient has a pulmonary embolism? *

Each answer must be between 0 and 100
Only an integer value may be entered in this field.

Please write your answer here:

© 2019 Brush JE Jr et al. JAMA Network Open.

Your initial probability answer was:
{INSERTANS:251728X3342X27225}

A quantitative D-dimer is measured. The value is less than 500, which is reported as negative.

Now what is the probability that the patient has a pulmonary embolism?

*

Each answer must be between 0 and 100
Only an integer value may be entered in this field.

Please write your answer here:

© 2019 Brush JE Jr et al. JAMA Network Open.

Session 2 - Case 11 of 20

A 28-year-old male presents to the emergency department for upper abdominal pain for the past 48 hours. The pain is worst in the right upper quadrant, but also present in the epigastrium and radiates to his back. The pain has been accompanied by nausea and vomiting and seemed to flare every time he has eaten in the last day. He reports a low-grade fever at home with associated chills. He has had two similar episodes of this pain in the past month. The pain resolved spontaneously during both episodes.

On one occasion he sought medical care at an urgent care clinic. An outpatient follow-up ultrasound revealed a single, mobile gallstone.

His past medical history is remarkable for allergic rhinitis. His only medication is loratidine.

On examination, his temperature is 38°C, blood pressure is 115/60 mmHg, pulse is 105 / minute, and respiratory rate 20/minute. The patient is morbidly obese with a BMI greater than 45. He appears uncomfortable but non-toxic. His abdomen is slightly distended, with tenderness and guarding on palpation of the right upper quadrant region. When asked to breathe deeply during palpation of his right upper quadrant, the patient stops inspiration and winces. He has no rashes.

On a scale of 0 (very IMprobable) to 100 (very PROBABLE), what is the probability that this patient has cholecystitis? *

Each answer must be between 0 and 100
Only an integer value may be entered in this field.

Please write your answer here:

© 2019 Brush JE Jr et al. JAMA Network Open.

Your initial probability answer was:
{INSERTANS:251728X3343X27228}

A bedside ultrasound is performed. It demonstrates a thickened gall bladder wall, pericholcystic fluid but no obstructing stone.

Now what is the probability that the patient has cholecystitis?

*

Each answer must be between 0 and 100
Only an integer value may be entered in this field.

Please write your answer here:

© 2019 Brush JE Jr et al. JAMA Network Open.

Session 2 - Case 12 of 20

A 22-year-old male arrived at the Emergency Department complaining of sudden onset headache, most notable at the base of his head. He experienced the same kind of headache last week but it went away on its own. Today's headache started when he was vigorously working out. The patient complains that the headache is centered at the base of his head.

The patient reports that the headache led to momentary lightheadedness, forcing him to stop exercising and lie down. Within a minute, the sensation of passing out resolved.

The patient's only medication is ibuprofen. He started taking it after his first headache last week. The patient has no known drug allergies

The patient is unemployed, but he indicates that he can afford purchasing medications.

On examination, the patient looks uncomfortable. His vital signs are: pulse 115/min; blood pressure 132/86 mmHg; respiratory rate18/min; and temperature 37.6°C. There is no papilledema. He has full range of neck motion. Examination of the central nervous system reveals a grossly normal cranial nerve exam. The motor exam of the extremities reveals an up going right toe on Babinski reflex testing. Cerebellar testing is normal. No rash is present.

On a scale of 0 (very IMprobable) to 100 (very PROBABLE), what is the probability that this patient has a subarachnoid hemorrhage? *

Each answer must be between 0 and 100
Only an integer value may be entered in this field.

Please write your answer here:

© 2019 Brush JE Jr et al. JAMA Network Open.

Your initial probability answer was: {INSERTANS:251728X3344X27231}

A lumbar puncture is performed. The fluid is pink on gross inspection. Cell count demonstrates > 5000 RBC per ml.

What is the probability that this patient has a subarachnoid hemorrhage?

*

Each answer must be between 0 and 100
Only an integer value may be entered in this field.

Please write your answer here:

© 2019 Brush JE Jr et al. JAMA Network Open.

Session 2 - Case 13 of 20

A 75-year-old man presents to the ED with chest pain. He has a history of CABG x 4 about 10 years ago. He takes aspirin daily but was unable to tolerate taking statins due to muscle aches. He is retired and walks his dog daily. Over the past week, he has noticed that he gets chest pain when he walks his dog. The pain is a dull ache in his mid-chest without radiation. This morning, he was awakened with severe chest pain radiating to the left arm. He broke out in a sweat and he felt mildly nauseated. His wife called 911 and an EKG in the field showed his chronic left bundle branch block. He is now complaining of 8/10 chest pain.

PMH is remarkable for CABG in the past and gastroesophageal reflux treated with Prilosec. He also takes aspirin and ezetimide.

On examination, HR=110, BP=130/80, RR=28. He is in moderate distress due to pain. HEENT is unremarkable. Lungs are clear and cardiac exam is normal.

On a scale of 0 (very IMprobable) to 100 (very PROBABLE), what is the probability that this patient has an acute myocardial infarction? *

Each answer must be between 0 and 100
Only an integer value may be entered in this field.

Please write your answer here:

Your initial probability answer was: {INSERTANS:251728X3345X27234}

A troponin level was drawn. The value is greater than 30, which is reported as abnormal.

Now what is the probability that this patient has an acute myocardial infarction?

*****

Each answer must be between 0 and 100
Only an integer value may be entered in this field.

Please write your answer here:

© 2019 Brush JE Jr et al. JAMA Network Open.

Session 2 - Case 14 of 20

A 50-year-old woman presents to the ED with a weeklong history of chest pain. She is otherwise healthy and quite active. Over the past week, she has notices a burning in her mid-chest without radiation. This will occur during the day and usually resolves after she has something to eat. She will also experience the pain at night after she goes to bed. She will usually fall asleep and she has noticed that the pain is usually gone when she awakens in the morning. She had a prolonged episode of pain this evening and her husband insisted that she get this checked out. He called 911 and she was brought in by ambulance. She is having mild pain on arrival in the ED.

PMH is remarkable for a history of hypertension, for which she takes a diuretic.

On examination, she is comfortable. HR=90, BP=130/85, RR=16. Lungs are clear and cardiac exam reveals a regular rate and rhythm and no murmurs. The abdomen is benign.

On a scale of 0 (very IMprobable) to 100 (very PROBABLE), what is the probability that this patient has an acute myocardial infarction? *

Each answer must be between 0 and 100
Only an integer value may be entered in this field.

Please write your answer here:

Your initial probability answer was: {INSERTANS:251728X3346X27237}

A troponin level was drawn. The value is less than 30, which is reported as normal.

Now what is the probability that this patient has an acute myocardial infarction?

*****

Each answer must be between 0 and 100
Only an integer value may be entered in this field.

Please write your answer here:

© 2019 Brush JE Jr et al. JAMA Network Open.

Session 2 - Case 15 of 20

A 70-year-old woman presents to the ED with chest pain. She has a history of coronary artery disease and about 5 years ago, she had a stent placed in the right coronary artery. She also has hypertension and hyperlipidemia, well controlled on medications. Her husband developed esophageal cancer and passed away last week. She has been under considerable stress since then. Her daughter is from out of town and arrived yesterday to help her. She mentioned to her daughter that she has been having chest pain over the past week. The pain is a vague discomfort in the mid chest. It does not feel like her previous angina. It occurs throughout the day and does not change with activity. It lasts about ½ hour and resolve spontaneously. It occurred this morning and didn't seem to go away. Her daughter called 911 and she was brought to the ED. And EKG in the field prior to arrival was normal. She is now complaining of 2/10 chest pressure.

PMH is remarkable for hypertension treated with metoprolol succinate and a thiazide diuretic. She also takes atorvastatin and aspirin.

On examination, HR=90, BP=140/90, RR=20. She appears comfortable. Lungs are clear and cardiac exam is unremarkable.

On a scale of 0 (very IMprobable) to 100 (very PROBABLE), what is the probability that this patient has an acute myocardial infarction? *

Each answer must be between 0 and 100
Only an integer value may be entered in this field.

Please write your answer here:

© 2019 Brush JE Jr et al. JAMA Network Open.

Your initial probability answer was:
{INSERTANS:251728X3347X27240}

A troponin level was drawn. The value is less than 30, which is reported as normal.

Now what is the probability that this patient has an acute myocardial infarction?

*

Each answer must be between 0 and 100
Only an integer value may be entered in this field.

Please write your answer here:

© 2019 Brush JE Jr et al. JAMA Network Open.

Session 2 - Case 16 of 20

A 70-year-old woman presents to the ED with chest pain. She has a history of coronary artery disease and underwent CABG x 4 8 years ago. Since then, she has done fairly well. She was working in her yard over the weekend, raking leaves. The next day, she noted a sharp pain in her chest that has recurred several times since then. This morning, when she awoke and got out of bed, she felt the sudden onset of a dull pain in her mid-chest without radiation. It seemed to worsen as she moved around while taking a shower this morning. Her husband became concerned and called 911. She is now complaining of 2/10 dull chest pain in a localized area in the mid chest without radiation or associated symptoms.

PMH is remarkable for a history of CABG, COPD, and hyperlipidemia. She takes atorvastatin, aspirin, and uses inhaled bronchodilators.

She lives with her husband and is a retired nurse. She continues to smoke ½ ppd and drinks an occasional glass of wine.

On examination, HR=90, BO=130/80, RR=20. She appears to be in mild discomfort. Lungs are clear to auscultation. Cardiac exam reveals a regular rate and rhythm and no murmurs. There is mild chest tenderness, but she is not sure if palpation exactly reproduces the pain that she has been experiencing since yesterday.

On a scale of 0 (very IMprobable) to 100 (very PROBABLE), what is the probability that this patient has an acute myocardial infarction? *

Each answer must be between 0 and 100
Only an integer value may be entered in this field.

Please write your answer here:

© 2019 Brush JE Jr et al. JAMA Network Open.

Your initial probability answer was:
{INSERTANS:251728X3348X27243}

A troponin level was drawn. The value is less than 30, which is reported as normal.

Now what is the probability that this patient has an acute myocardial infarction?

*****

Each answer must be between 0 and 100
Only an integer value may be entered in this field.

Please write your answer here:

© 2019 Brush JE Jr et al. JAMA Network Open.

Session 2 - Case 17 of 20

An 86-year-old woman is transferred to the emergency department from her nursing home. Over the last two days she has developed a new cough, fever and has become increasingly confused. At baseline she has mild cognitive impairment but is normally able to participate in a simple conversation. Now she is lethargic and her speech is illegible. The nursing home transfer record indicates that she has been coughing up dark green sputum.

Her past medical history is remarkable for hypertension, diet-controlled diabetes, coronary artery disease, a remote stroke (which left her with left-sided paralysis) and mild dementia. She is a non-smoker and does not drink alcohol.

On physical examination, her vital signs are temperature 39.5 C; pulse 118 / minute, blood pressure 120/96 / minute; respiratory rate 30 / minute. Her cardiac exam reveals a regular rate and rhythm with no murmurs, gallops or rubs. On auscultation of her chest, she has crackles in the left base. Her oropharynx and neck exam reveal no abnormalities. Her extremities have no swelling, and there are no visible rashes.

On a scale of 0 (very IMprobable) to 100 (very PROBABLE), what is the probability that this patient has pneumonia? *

Each answer must be between 0 and 100
Only an integer value may be entered in this field.

Please write your answer here:

Your initial probability answer was:
{INSERTANS:251728X3349X27246}

A chest x-ray is performed. The radiologist interprets the chest x-ray as showing evidence of consolidation.

What is the probability that this patient has pneumonia?

*

Each answer must be between 0 and 100
Only an integer value may be entered in this field.

Please write your answer here:

© 2019 Brush JE Jr et al. JAMA Network Open.

Session 2 - Case 18 of 20

A 57-year-old man presents to the emergency department for evaluation of 5 days of a cough, productive of green sputum. He has been feeling generally weak and tired over this same time period. He complains of a sore throat and coryza. He thinks he may have a fever and notes several episodes of rigors. He has had no nausea or vomiting, and reports no rashes or joint pains. He denies chest pain. He blames his teenage daughter for giving him the virus she just recovered from.

His past medical history is unremarkable. He quickly acknowledges that he doesn't believe in medications and doesn't take them. In fact, he has never had the influenza vaccine. He has no allergies. He is a social smoker and does not use any illicit drugs.

On examination, his temperature is 39.1C, pulse 106/minute; blood pressure 110/96mmHg; and respiratory rate 25/minute.

In general, he appears tired but non-toxic, and is speaking full sentences. He has dry mucous membranes, his oropharynx has no erythema or exudate, and he has no anterior or posterior chain cervical lymphadenopathy. On chest exam, he has crackles at the right base. His cardiac exam is normal. His skin demonstrates no rashes, petechiae, or purpura.

On a scale of 0 (very IMprobable) to 100 (very PROBABLE), what is the probability that this patient has pneumonia? *

Each answer must be between 0 and 100
Only an integer value may be entered in this field.

Please write your answer here:

© 2019 Brush JE Jr et al. JAMA Network Open.

Your initial probability answer was:
{INSERTANS:251728X3350X27249}

A chest x-ray is performed. The radiologist interprets the chest x-ray as no evidence of consolidation.

What is the probability that this patient has pneumonia?

*

Each answer must be between 0 and 100
Only an integer value may be entered in this field.

Please write your answer here:

© 2019 Brush JE Jr et al. JAMA Network Open.

Session 2 - Case 19 of 20

A 30-year-old man presents to the emergency department complaining of worsening cough and shortness of breath for the last two days. His illness began 2 days prior to the cough when he started to experience malaise, and low-grade fever. When his breathing started to get worse, he increased his inhaled bronchodilator use and started an inhaled corticosteroid, thinking his asthma was flaring. He presents now because the medications are not working and he is coughing up yellow sputum.

His past medical history only includes well controlled asthma. His typical flare is caused by a viral upper respiratory infection. He is a regular smoker but has drastically reduced this week with a promise to quit because he feels so unwell.

On examination his temperature is 37.9C, pulse 116/minute; blood pressure 139/96mmHg; and respiratory rate 25/minute.

In general, he appears breathless. His cardiac exam is within normal limits. His respiratory exam reveals moderate wheezes bilaterally in all lung fields. There are no associated adventitious sounds, crackles or rales.

On a scale of 0 (very IMprobable) to 100 (very PROBABLE), what is the probability that this patient has pneumonia? *

Each answer must be between 0 and 100
Only an integer value may be entered in this field.

Please write your answer here:

© 2019 Brush JE Jr et al. JAMA Network Open.

Your initial probability answer was:
{INSERTANS:251728X3351X27252}

A chest x-ray is performed. The radiologist interprets the chest x-ray as showing evidence of consolidation.

What is the probability that this patient has pneumonia?

*

Each answer must be between 0 and 100
Only an integer value may be entered in this field.

Please write your answer here:

© 2019 Brush JE Jr et al. JAMA Network Open.

Session 2 - Case 20 of 20

A 23-year-old woman presents to the emergency department complaining of cough, sore throat and coryza for the past three days. She has not been feeling herself this week. Her boyfriend was recently seen by his family physician for a similar illness and treated with conservative measures. She notes that she can't get sick; work is busy this week. "This is the reason I got the flu shot."

Her past medical history is remarkable for mitral valve prolapse. Her only medication is an oral contraceptive pill. She smokes only in social settings but has been smoking more this week because of job stress. She is allergic to NSAIDs, and because of this she has avoided any over-the-counter medications for her symptoms.

On examination, her temperature is 37.1C; pulse 96 / minute; blood pressure 108/96mmHg; and respiratory rate 16 / minute. Her chest is resonant to percussion bilaterally. There are no crackles or adventitious sounds on bilateral auscultation. Her heart is regular with a fast rate with a 2/6 holosystolic murmur heard loudest at the apex without radiation. She has an inflamed oropharynx with bilateral tender, enlarged anterior cervical lymph nodes.

On a scale of 0 (very IMprobable) to 100 (very PROBABLE), what is the probability that this patient has pneumonia? *

Each answer must be between 0 and 100
Only an integer value may be entered in this field.

Please write your answer here:

Your initial probability answer was: {INSERTANS:251728X3352X27255}

A chest x-ray is performed. The radiologist interprets the chest x-ray as no evidence of consolidation.

What is the probability that this patient has pneumonia?

*

Each answer must be between 0 and 100
Only an integer value may be entered in this field.

Please write your answer here:

© 2019 Brush JE Jr et al. JAMA Network Open.

© 2019 Brush JE Jr et al. JAMA Network Open.

Demographics

Birth Year (YYYY) *

Only numbers may be entered in this field.

Please write your answer here:

What year of medical school are you in? *

Only numbers may be entered in this field.

Please write your answer here:

Your current medical school: *

Please choose **only one** of the following:

- Eastern Virginia Medical School
- Michael G. DeGroote School of Medicine - McMaster University
- Other

© 2019 Brush JE Jr et al. JAMA Network Open.

Last Question. Please estimate the number of patients you have seen with the following diagnoses. *

Please choose the appropriate response for each item:

	less than 5	5 - 20	more than 20
Pneumonia	○	○	○
Congestive heart failure	○	○	○
Pulmonary Embolism	○	○	○
Acute coronary syndrome	○	○	○

© 2019 Brush JE Jr et al. JAMA Network Open.

Submit your survey.
Thank you for completing this survey.

© 2019 Brush JE Jr et al. JAMA Network Open.

Test for Concept Group

1. Instructions
2. Test questions

© 2019 Brush JE Jr et al. JAMA Network Open.

EBM T2 test for video condition

There are 45 questions in this survey

Instructions

Welcome back! We very much appreciate your participation in this study.

In this session, you will be presented with a total of 20 cases.

As in the first session, you will be asked for a probability for the diagnosis for each case, after clinical and likelihood information is presented. Then, a test result will be given and you will be asked for a post-test probability.

YOU MUST COMPLETE ALL OF THE CASES IN ONE SITTING. If you don't have approximately 30 minutes at the moment, please click on the "Exit and clear survey" button at the bottom of this page and come back to it later using the same link BUT remember that the survey expires 72 hours after you received the email with the link for this session.

AFTER you complete the test you will be directed to a separate survey to complete information regarding the HONORARIUM. A new page will be opened so that we cannot link your answers to your contact information.

Thanks.

© 2019 Brush JE Jr et al. JAMA Network Open.

Session 2 - Case 1 of 20

A 28 year-old woman presents to the ED with a 3-day history of shortness of breath. She also complains of a dry cough and sore throat. Over the past 3 days, she has had mild chills but hasn't taken her temperature. For the last 3 days she has shortness of breath when she climbs the stairs of her apartment building carrying groceries. She sleeps flat at night and denies ankle edema.

Her past medical history is unremarkable. She denies hypertension and only takes birth control pills. She has no known allergies.

On examination, she is afebrile, HR=98, BP=120/80. RR=20. Her throat is slightly erythematous and she has mildly enlarged cervical lymph nodes. Lungs are clear to auscultation and her heart exam is normal.

On a scale of 0 (very IMprobable) to 100 (very PROBABLE), what is the probability that this patient has congestive heart failure? *

Each answer must be between 0 and 100
Only an integer value may be entered in this field.

Please write your answer here:

© 2019 Brush JE Jr et al. JAMA Network Open.

Your initial probability answer was:
{INSERTANS:156367X3353X27262}

A chest x-ray is performed. The radiologist interprets the x-ray as negative for pulmonary venous congestion.

Studies show that a chest x-ray showing pulmonary venous congestion is predictive of congestive heart failure with a sensitivity of 54%, specificity of 96%, and a positive likelihood ratio of 13.5 and a negative likelihood ratio of 0.5.

Now what is the probability that the patient has congestive heart failure?

*

Each answer must be between 0 and 100
Only an integer value may be entered in this field.

Please write your answer here:

© 2019 Brush JE Jr et al. JAMA Network Open.

Session 2 - Case 2 of 20

A 50-year-old African American woman presents to the ED with progressive shortness of breath. She has longstanding hypertension, obesity, and type 2 diabetes mellitus. Over the years, she developed progressive renal failure and one year ago was placed on hemodialysis. Over the past two weeks she has developed increasing dyspnea on exertion. Over the last three days she has developed sinus congestion, myalgia and a dry cough. She now has to go slowly when she climbs the stairs up to her second-story apartment. She has always slept on 2 pillows. She has noticed some increasing ankle edema lately and finds that her hands appear a bit puffy making her wedding ring feel tight.

PMH-hypertension, diabetes, end-stage renal disease on dialysis. Her medications are losartan, amlodipine, glyburide, and calcitriol.

The patient lives locally with her husband and is no longer working. She continues to smoke ½ ppd and drinks an occasional beer.

On exam, she is afebrile, HR-80, BP-170/80, RR=28. She is obese and in mild respiratory distress. There is minimal jugular venous distension. Lung exam reveals occasional scattered wheeze and cardiac exam is unremarkable except for bilateral 2+ ankle edema.

On a scale of 0 (very IMprobable) to 100 (very PROBABLE), what is the probability that this patient has congestive heart failure? *

Each answer must be between 0 and 100
Only an integer value may be entered in this field.

Please write your answer here:

© 2019 Brush JE Jr et al. JAMA Network Open.

Your initial probability answer was:
{INSERTANS:156367X3356X27270}

A chest x-ray is performed. The radiologist interprets the x-ray as negative for pulmonary venous congestion.

Studies show that a chest x-ray showing pulmonary venous congestion is predictive of congestive heart failure with a sensitivity of 54%, specificity of 96%, and a positive likelihood ratio of 13.5 and a negative likelihood ratio of 0.5.

Now what is the probability that the patient has congestive heart failure?

*

Each answer must be between 0 and 100
Only an integer value may be entered in this field.

Please write your answer here:

© 2019 Brush JE Jr et al. JAMA Network Open.

Session 2 - Case 3 of 20

A 60-year-old man presents to the ED with progressive shortness of breath. The patient lives in a nearby homeless shelter, is currently unemployed, and has a long-standing history of alcohol abuse. He continues to drink about a pint of alcohol daily. He was transferred from the homeless shelter because of severe shortness of breath and acute intoxication. He is a difficult historian but does give a history of severe shortness of breath with minimal exertion over the past month. He has trouble sleeping at night and has found that he can get some sleep if he sits up in a chair or leans over a table while sleeping. He has notices that his abdomen has become distended and he has swelling in the ankles. He denies chest pain, palpitations, or lightheadedness. He denies cough or fever.

Past medical history is remarkable for a history of hospitalizations in the past for acute alcohol intoxication, but otherwise, he has avoided contact with doctors and has no other reported past medical problems. He takes no medications.

He lives at the homeless shelter. He is divorced and unemployed, formerly working as a school custodian. He smokes a few cigarettes daily and drinks a pint of vodka daily.

On exam, HR=115, BP=90/60, RR=28. He is disheveled and smells of alcohol and appears intoxicated and acutely short of breath. He has jugular venous distention, bibasilar rales, an S3 gallop, and 3+ bilateral pitting edema. Abdominal exam reveals an enlarged liver and possible ascites.

On a scale of 0 (very IMprobable) to 100 (very PROBABLE), what is the probability that this patient has congestive heart failure? *

Each answer must be between 0 and 100
Only an integer value may be entered in this field.

Please write your answer here:

© 2019 Brush JE Jr et al. JAMA Network Open.

Your initial probability answer was: {INSERTANS:156367X3357X27273}

A chest x-ray is performed. The radiologist interprets the x-ray as positive for pulmonary venous congestion.

Studies show that a chest x-ray showing pulmonary venous congestion is predictive of congestive heart failure with a sensitivity of 54%, specificity of 96%, and a positive likelihood ratio of 13.5 and a negative likelihood ratio of 0.5.

Now what is the probability that the patient has congestive heart failure?

*

Each answer must be between 0 and 100
Only an integer value may be entered in this field.

Please write your answer here:

© 2019 Brush JE Jr et al. JAMA Network Open.

Session 2 - Case 4 of 20

A 70 year-old man presents to the ED with progressive shortness of breath. He had an inferior ST elevation myocardial infarction 5 years ago treated with emergency stenting of the right coronary artery. He is obese and has a history of obstructive sleep apnea. He uses CPAP at night. Over the past month and a half, he has noticed a marked increase in his shortness of breath. He is now short of breath when he plays golf and does routine things around the house. He sleeps flat at night using CPAP and has had mild ankle edema.

PMH is remarkable for CAD and obstructive sleep apnea, as well as hypertension and type-2 diabetes mellitus.

He lives with his wife. He quit smoking when he had his heart attack 5 years ago and drinks one mixed drink each night.

On exam, he is afebrile, HR=100, BP=150/80, RR=18. His neck is obese and the jugular veins are not visualized. Lungs reveals fine bibasilar rales. Cardiac exam reveals no extra heart sounds. He has 1+ ankle edema.

On a scale of 0 (very IMprobable) to 100 (very PROBABLE), what is the probability that this patient has congestive heart failure? *

Each answer must be between 0 and 100
Only an integer value may be entered in this field.

Please write your answer here:

© 2019 Brush JE Jr et al. JAMA Network Open.

Your initial probability answer was:
{INSERTANS:156367X3358X27276}

A chest x-ray is performed. The radiologist interprets the x-ray as negative for pulmonary venous congestion.

Studies show that a chest x-ray showing pulmonary venous congestion is predictive of congestive heart failure with a sensitivity of 54%, specificity of 96%, and a positive likelihood ratio of 13.5 and a negative likelihood ratio of 0.5.

Now what is the probability that the patient has congestive heart failure?

*

Each answer must be between 0 and 100
Only an integer value may be entered in this field.

Please write your answer here:

© 2019 Brush JE Jr et al. JAMA Network Open.

Session 2 - Case 5 of 20

A 28-year-old woman arrives in the emergency department with her roommate for acute onset flank pain. She says the pain is a constant dull ache; however, it regularly increases in intensity to become nearly unbearable before dying back down. The pain radiates into her groin. For the past three days she has fell unwell. The patient also notes discomfort with urination. Today she has noticed gross hematuria when she urinated. She has never had this pain before.

The patient has a history of urinary tract infections. She also has adult ADHD (attention deficit hyperactivity disorder) for which she self-medicates with marijuana. She is a graduate student who has taken a leave of absence to train for the national triathlon team.

On physical examination she appears uncomfortable. Occasionally, she is unable to lay still, but she will then settle complaining of a dull pain. Her temperature is 37.2C; pulse 105/minute; blood pressure 150/95mmHg; respiratory rate 20/minute. Her chest is clear to auscultation and heart sounds are normal but with a regular tachycardic rate. Her abdomen is non-tender and soft. She has left costovertebral angle tenderness to percussion.

On a scale of 0 (very IMprobable) to 100 (very PROBABLE), what is the probability that this patient has pyelonephritis? *

Each answer must be between 0 and 100
Only an integer value may be entered in this field.

Please write your answer here:

© 2019 Brush JE Jr et al. JAMA Network Open.

Your initial probability answer was:
{INSERTANS:156367X3359X27279}

A urinalysis is performed. It demonstrates 3+RBC, 1+WBC, dipstick urinalysis was positive for nitrates and leukocyte esterase.

Studies show that a dipstick urinalysis positive for nitrates and leukocyte esterase is predictive of urinary tract infection with a sensitivity of 75%, specificity of 82%, and a positive likelihood ratio of 4.2 and a negative likelihood ratio of 0.3.

Now what is the probability that the patient has pyelonephritis?

*

Each answer must be between 0 and 100
Only an integer value may be entered in this field.

Please write your answer here:

© 2019 Brush JE Jr et al. JAMA Network Open.

Session 2 - Case 6 of 20

A 75 yo woman presents to the emergency department for acute onset right knee pain and swelling that awoke her from her sleep. She denies trauma to the limb, although she recalls having a heavy box fall from a shelf and land on her leg yesterday. She walks with considerable pain but is able to navigate the waiting room of the emergency department to her exam room. There is no history of fevers or chills. She denies any recent camping or tick bites. She has not noted any rashes.

Her past medical history includes podagara (gout of the first metatarsal phalynx joint) and diabetes mellitus.

She is currently taking glyburide for her diabetes and has no known allergies.

On physical examination, she is nontoxic appearing. Her temperature is 37.5 C; pulse 86 / minute; blood pressure 125/70 mmHg; respiratory rate 16 / minute. Her cardiac exam is normal. Her right knee is slightly red and warm. There is an obvious effusion. While there is pain with movement, she is able to fully flex and extend the knee, although end of range is restricted by the effusion.

On a scale of 0 (very IMprobable) to 100 (very PROBABLE), what is the probability that this patient has crystal arthropathy? *

Each answer must be between 0 and 100
Only an integer value may be entered in this field.

Please write your answer here:

© 2019 Brush JE Jr et al. JAMA Network Open.

Your initial probability answer was:
{INSERTANS:156367X3360X27282}

An arthrocentesis is performed. It demonstrates 50,0000 WBC /ml and negatively birefringent crystals.

Studies show that a joint aspirate showing crystals is predictive of crystal arthropathy with a sensitivity of 84, specificity of 99% with a positive likelihood ratio of 84 and a negative likelihood ratio of 0.2.

Now what is the probability that the patient has crystal arthropathy?

*

Each answer must be between 0 and 100
Only an integer value may be entered in this field.

Please write your answer here:

© 2019 Brush JE Jr et al. JAMA Network Open.

Session 2 - Case 7 of 20

A 37-year-old male presents to the emergency department for evaluation of five days of a malaise with cough. He felt like he has had a fever though he does not have a thermometer at home. He is coming to the emergency department because he is now experiencing sharp anterior chest pain. The pain is worse with inspiration. He also notes that if he bends forward the pain is minimal, but if he lies flat on his back, the pain is intense. He complains of shortness of breath on exertion

His past medical history is remarkable for hypertension, for which he takes atenolol. He has no allergies. He drinks alcohol rarely, and smokes ½ ppd, though does not use any illicit drugs.

On examination, his temperature was 37.9, pulse 96, BP 150/96, and RR 15. In general, he appears tired but non-toxic, and is speaking full sentences. He has dry mucous membranes; his oropharynx has no erythema or exudate, and he has no anterior or posterior chain cervical lymphadenopathy. On chest exam, he has crackles at the right base. His heart has a regular rate and rhythm. The cardiac exam is normal. Examination of his extremities demonstrates a tender calf on the right but not obvious swelling. His skin demonstrates no rashes, petechiae, or purpura.

On a scale of 0 (very IMprobable) to 100 (very PROBABLE), what is the probability that this patient has a pulmonary embolism? *

Each answer must be between 0 and 100
Only an integer value may be entered in this field.

Please write your answer here:

© 2019 Brush JE Jr et al. JAMA Network Open.

Your initial probability answer was:
{INSERTANS:156367X3361X27285}

A quantitative D-dimer is measured. The value is greater than 500, which is reported as positive.

Studies show that a quantitative d-dimer is predictive of pulmonary embolism with a sensitivity of 96% and a specificity of 45% with a positive likelihood ratio of 1.7 and a negative likelihood ratio of 0.09.

What is the probability that this patient has a pulmonary embolism?

*

Each answer must be between 0 and 100
Only an integer value may be entered in this field.

Please write your answer here:

© 2019 Brush JE Jr et al. JAMA Network Open.

Session 2 - Case 8 of 20

A 29 yo female presents complaining of pleuritic chest pain. The pain has been waxing and waning for the last 24 hours. The patient does not recall any precipitating factors, specifically trauma or upper respiratory symptoms. When the pain did not resolve on its own she sought medical attention. She noted that although she has no cough, when she walked up the stairs from the parking garage she was breathless at the top. This is unusual for her as she is active and in shape. Her biggest complaint is that she can only take tiny breaths, any deep inspiration intensifies the sharp chest pain across her right anterior chest.

Her past medical history is unremarkable. She has no history of cardiac or pulmonary disease. Her only medication is an oral contraceptive pill. She still smokes, mostly on the weekends in social situations, but is actively reducing the amount she smokers per day.

On examination, her temperature is 36.9, pulse 135, BP 107/80, and RR 26. Her chest is clear to auscultation. There is no tenderness on palpation. Her cardiac exam is unremarkable. Her right calf is obviously swollen in comparison to her other legs. On palpation of the right calf she notes that it is slightly tender, a symptom she had not noticed prior to the exam.

On a scale of 0 (very IMprobable) to 100 (very PROBABLE), what is the probability that this patient has a pulmonary embolism? *

Each answer must be between 0 and 100
Only an integer value may be entered in this field.

Please write your answer here:

© 2019 Brush JE Jr et al. JAMA Network Open.

Your initial probability answer was: {INSERTANS:156367X3362X27288}

A quantitative D-dimer is measured. The value is greater than 500, which is reported as positive.

Studies show that a quantitative d-dimer is predictive of pulmonary embolism with a sensitivity of 96% and a specificity of 45% with a positive likelihood ratio of 1.7 and a negative likelihood ratio of 0.09.

What is the probability that this patient has a pulmonary embolism?

*

Each answer must be between 0 and 100
Only an integer value may be entered in this field.

Please write your answer here:

© 2019 Brush JE Jr et al. JAMA Network Open.

Session 2 - Case 9 of 20

A 53-year-old female presents to the emergency department because of shortness of breath. The dyspnea occurred suddenly and awoke the patient from sleep. The patient also reports that she is having retrosternal chest pain, which is worse on deep breathing. The patient reports that she has been feeling generally tired for about 4 days, with a sinus congestion as well as fevers and chills. She has also had a cough for several days and had coughed up yellow-green sputum with occasional flecks of blood. The patient also reports that he is having nausea. She vomited a small amount of bile during the triage interview. She has had no recent surgery. She recently returned from a trip to Bali and complains of the lack of space in an economy seat.

Her past medical history is remarkable for a tubal ligation 13 years ago and pneumonia two years ago. She takes no medications and has no allergies. She smoked previously but stopped 8 years ago when she became pregnant with her first child.

On examination, her temperature is 38.0, pulse 116, BP 110/96, and RR 20. On auscultation of her chest, she has no adventitial breath sounds. Her heart is regular rate and rhythm, with no murmurs, gallops, or rubs. There was trace pitting edema in both of her ankles.

On a scale of 0 (very IMprobable) to 100 (very PROBABLE), what is the probability that this patient has a pulmonary embolism? *

Each answer must be between 0 and 100
Only an integer value may be entered in this field.

Please write your answer here:

© 2019 Brush JE Jr et al. JAMA Network Open.

Your initial probability answer was: {INSERTANS:156367X3363X27291}

A quantitative D-dimer is measured. The value is less than 500, which is reported as negative.

Studies show that a quantitative d-dimer is predictive of pulmonary embolism with a sensitivity of 96% and a specificity of 45% with a positive likelihood ratio of 1.7 and a negative likelihood ratio of 0.09.

What is the probability that this patient has a pulmonary embolism?

*

Each answer must be between 0 and 100
Only an integer value may be entered in this field.

Please write your answer here:

© 2019 Brush JE Jr et al. JAMA Network Open.

Session 2 - Case 10 of 20

A 45-year-old female presents with a 6 day history of difficulty breathing. “It feels as though I’m breathing through a wet towel.” The patient denies any chest pain. She has been using a Ventolin inhaler without significant relief for the past three days. She indicates that she has been diagnosed with asthma by her family physician without formal testing; however, her condition is so mild that she infrequently (less than once a year) requires bronchodilators. “I wonder if my puffer is useless, I got it last year and haven’t touched it since.”

Past medical history is only significant for asthma. The patient is a smoker but has cut down to less than a quarter package of cigarettes per day. She takes a multivitamin and a vitamin D tablet daily.

On examination, her temperature is 36.9C; pulse 106 / minute; blood pressure 100/70mmHg; and respiratory rate 22 / minute. She has equal strong pulses in all limbs. Her cardiac exam reveals no rubs, or murmurs. She has faint scattered wheezes in all lung fields. She can speak in full sentences, although she is occasionally interrupted by the need to cough and clear her throat. She has no calf tenderness or swelling.

On a scale of 0 (very IMprobable) to 100 (very PROBABLE), what is the probability that this patient has a pulmonary embolism? *

Each answer must be between 0 and 100
Only an integer value may be entered in this field.

Please write your answer here:

© 2019 Brush JE Jr et al. JAMA Network Open.

Your initial probability answer was:
{INSERTANS:156367X3364X27294}

A quantitative D-dimer is measured. The value is less than 500, which is reported as negative.

Studies show that a quantitative d-dimer is predictive of pulmonary embolism with a sensitivity of 96% and a specificity of 45% with a positive likelihood ratio of 1.7 and a negative likelihood ratio of 0.09.

What is the probability that this patient has a pulmonary embolism? *

Each answer must be between 0 and 100
Only an integer value may be entered in this field.

Please write your answer here:

© 2019 Brush JE Jr et al. JAMA Network Open.

Session 2 - Case 11 of 20

A 28-year-old male presents to the emergency department for upper abdominal pain for the past 48 hours. The pain is worst in the right upper quadrant, but also present in the epigastrium and radiates to his back. The pain has been accompanied by nausea and vomiting and seemed to flare every time he has eaten in the last day. He reports a low-grade fever at home with associated chills. He has had two similar episodes of this pain in the past month. The pain resolved spontaneously during both episodes.

On one occasion he sought medical care at an urgent care clinic. An outpatient follow-up ultrasound revealed a single, mobile gallstone.

His past medical history is remarkable for allergic rhinitis. His only medication is loratidine.

On examination, his temperature is 38°C, blood pressure is 115/60 mmHg, pulse is 105 / minute, and respiratory rate 20/minute. The patient is morbidly obese with a BMI greater than 45. He appears uncomfortable but non-toxic. His abdomen is slightly distended, with tenderness and guarding on palpation of the right upper quadrant region. When asked to breathe deeply during palpation of his right upper quadrant, the patient stops inspiration and winces. He has no rashes.

On a scale of 0 (very IMprobable) to 100 (very PROBABLE), what is the probability that this patient has cholecystitis? *

Each answer must be between 0 and 100
Only an integer value may be entered in this field.

Please write your answer here:

© 2019 Brush JE Jr et al. JAMA Network Open.

Your initial probability answer was:
{INSERTANS:156367X3365X27297}

A bedside ultrasound is performed. It demonstrates a thickened gall bladder wall, pericholcystic fluid but no obstructing stone.

Studies show that bedside ultrasound is predictive for acute cholecystitis with a sensitivity of 88% and specificity of 80% with a positive likelihood ratio of 4.4 and negative likelihood ratio of 0.1.

Now what is the probability that the patient has cholecystitis?

*

Each answer must be between 0 and 100
Only an integer value may be entered in this field.

Please write your answer here:

© 2019 Brush JE Jr et al. JAMA Network Open.

Session 2 - Case 12 of 20

A 22-year-old male arrived at the Emergency Department complaining of sudden onset headache, most notable at the base of his head. He experienced the same kind of headache last week but it went away on its own. Today's headache started when he was vigorously working out. The patient complains that the headache is centered at the base of his head.

The patient reports that the headache led to momentary lightheadedness, forcing him to stop exercising and lie down. Within a minute, the sensation of passing out resolved.

The patient's only medication is ibuprofen. He started taking it after his first headache last week. The patient has no known drug allergies

The patient is unemployed, but he indicates that he can afford purchasing medications.

On examination, the patient looks uncomfortable. His vital signs are: pulse 115/min; blood pressure 132/86 mmHg; respiratory rate18/min; and temperature 37.6°C. There is no papilledema. He has full range of neck motion. Examination of the central nervous system reveals a grossly normal cranial nerve exam. The motor exam of the extremities reveals an up going right toe on Babinski reflex testing. Cerebellar testing is normal. No rash is present.

On a scale of 0 (very IMprobable) to 100 (very PROBABLE), what is the probability that this patient has a subarachnoid hemorrhage? *

Each answer must be between 0 and 100
Only an integer value may be entered in this field.

Please write your answer here:

© 2019 Brush JE Jr et al. JAMA Network Open.

Your initial probability answer was:
{INSERTANS:156367X3366X27300}

A lumbar puncture is performed. The fluid is pink on gross inspection. Cell count demonstrates > 5000 RBC per ml.

Studies show that a lumber puncture showing blood in the cerebrospinal fluid is predictive for subarachnoid hemorrhage with a sensitivity of 99% and a specificity of 80% with a positive likelihood ratio of 80 and a negative likelihood ratio of 0.2.

What is the probability that this patient has subarachnoid hemorrhage?

*

Each answer must be between 0 and 100
Only an integer value may be entered in this field.

Please write your answer here:

© 2019 Brush JE Jr et al. JAMA Network Open.

Session 2 - Case 13 of 20

A 75-year-old man presents to the ED with chest pain. He has a history of CABG x 4 about 10 years ago. He takes aspirin daily but was unable to tolerate taking statins due to muscle aches. He is retired and walks his dog daily. Over the past week, he has noticed that he gets chest pain when he walks his dog. The pain is a dull ache in his mid-chest without radiation. This morning, he was awakened with severe chest pain radiating to the left arm. He broke out in a sweat and he felt mildly nauseated. His wife called 911 and an EKG in the field showed his chronic left bundle branch block. He is now complaining of 8/10 chest pain.

PMH is remarkable for CABG in the past and gastroesophageal reflux treated with Prilosec. He also takes aspirin and ezetimide.

On examination, HR=110, BP=130/80, RR=28. He is in moderate distress due to pain. HEENT is unremarkable. Lungs are clear and cardiac exam is normal.

On a scale of 0 (very IMprobable) to 100 (very PROBABLE), what is the probability that this patient has an acute myocardial infarction? *

Each answer must be between 0 and 100
Only an integer value may be entered in this field.

Please write your answer here:

© 2019 Brush JE Jr et al. JAMA Network Open.

Your initial probability answer was:
{INSERTANS:156367X3367X27303}

A troponin level was drawn. The value is greater than 30, which is reported as abnormal.

Studies show that troponin testing in this setting has a sensitivity of 95%, specificity of 80% and a positive likelihood ratio of 4.75 and a negative likelihood ratio of 0.1.

Now what is the probability that this patient has an acute myocardial infarction?

*

Each answer must be between 0 and 100
Only an integer value may be entered in this field.

Please write your answer here:

© 2019 Brush JE Jr et al. JAMA Network Open.

Session 2 - Case 14 of 20

A 50-year-old woman presents to the ED with a weeklong history of chest pain. She is otherwise healthy and quite active. Over the past week, she has notices a burning in her mid-chest without radiation. This will occur during the day and usually resolves after she has something to eat. She will also experience the pain at night after she goes to bed. She will usually fall asleep and she has noticed that the pain is usually gone when she awakens in the morning. She had a prolonged episode of pain this evening and her husband insisted that she get this checked out. He called 911 and she was brought in by ambulance. She is having mild pain on arrival in the ED.

PMH is remarkable for a history of hypertension, for which she takes a diuretic.

On examination, she is comfortable. HR=90, BP=130/85, RR=16. Lungs are clear and cardiac exam reveals a regular rate and rhythm and no murmurs. The abdomen is benign.

On a scale of 0 (very IMprobable) to 100 (very PROBABLE), what is the probability that this patient has an acute myocardial infarction? *

Each answer must be between 0 and 100
Only an integer value may be entered in this field.

Please write your answer here:

© 2019 Brush JE Jr et al. JAMA Network Open.

Your initial probability answer was:
{INSERTANS:156367X3368X27306}

A troponin level was drawn. The value is less than 30, which is reported as normal.

Studies show that troponin testing in this setting has a sensitivity of 95%, specificity of 80% and a positive likelihood ratio of 4.75 and a negative likelihood ratio of 0.1.

Now what is the probability that this patient has an acute myocardial infarction?

*

Each answer must be between 0 and 100
Only an integer value may be entered in this field.

Please write your answer here:

© 2019 Brush JE Jr et al. JAMA Network Open.

Session 2 - Case 15 of 20

A 70-year-old woman presents to the ED with chest pain. She has a history of coronary artery disease and about 5 years ago, she had a stent placed in the right coronary artery. She also has hypertension and hyperlipidemia, well controlled on medications. Her husband developed esophageal cancer and passed away last week. She has been under considerable stress since then. Her daughter is from out of town and arrived yesterday to help her. She mentioned to her daughter that she has been having chest pain over the past week. The pain is a vague discomfort in the mid chest. It does not feel like her previous angina. It occurs throughout the day and does not change with activity. It lasts about ½ hour and resolve spontaneously. It occurred this morning and didn't seem to go away. Her daughter called 911 and she was brought to the ED. And EKG in the field prior to arrival was normal. She is now complaining of 2/10 chest pressure.

PMH is remarkable for hypertension treated with metoprolol succinate and a thiazide diuretic. She also takes atorvastatin and aspirin.

On examination, HR=90, BP=140/90, RR=20. She appears comfortable. Lungs are clear and cardiac exam is unremarkable.

On a scale of 0 (very IMprobable) to 100 (very PROBABLE), what is the probability that this patient has an acute myocardial infarction? *

Each answer must be between 0 and 100
Only an integer value may be entered in this field.

Please write your answer here:

© 2019 Brush JE Jr et al. JAMA Network Open.

Your initial probability answer was:
{INSERTANS:156367X3369X27309}

A troponin level was drawn. The value is less than 30, which is reported as normal.

Studies show that troponin testing in this setting has a sensitivity of 95%, specificity of 80% and a positive likelihood ratio of 4.75 and a negative likelihood ratio of 0.1.

Now what is the probability that this patient has an acute myocardial infarction?

*

Each answer must be between 0 and 100
Only an integer value may be entered in this field.

Please write your answer here:

© 2019 Brush JE Jr et al. JAMA Network Open.

Session 2 - Case 16 of 20

A 70-year-old woman presents to the ED with chest pain. She has a history of coronary artery disease and underwent CABG x 4 8 years ago. Since then, she has done fairly well. She was working in her yard over the weekend, raking leaves. The next day, she noted a sharp pain in her chest that has recurred several times since then. This morning, when she awoke and got out of bed, she felt the sudden onset of a dull pain in her mid-chest without radiation. It seemed to worsen as she moved around while taking a shower this morning. Her husband became concerned and called 911. She is now complaining of 2/10 dull chest pain in a localized area in the mid chest without radiation or associated symptoms.

PMH is remarkable for a history of CABG, COPD, and hyperlipidemia. She takes atorvastatin, aspirin, and uses inhaled bronchodilators.

She lives with her husband and is a retired nurse. She continues to smoke ½ ppd and drinks an occasional glass of wine.

On examination, HR=90, BO=130/80, RR=20. She appears to be in mild discomfort. Lungs are clear to auscultation. Cardiac exam reveals a regular rate and rhythm and no murmurs. There is mild chest tenderness, but she is not sure if palpation exactly reproduces the pain that she has been experiencing since yesterday.

On a scale of 0 (very IMprobable) to 100 (very PROBABLE), what is the probability that this patient has an acute myocardial infarction? *

Each answer must be between 0 and 100
Only an integer value may be entered in this field.

Please write your answer here:

© 2019 Brush JE Jr et al. JAMA Network Open.

Your initial probability answer was:
{INSERTANS:156367X3370X27312}

A troponin level was drawn. The value is less than 30, which is reported as normal.

Studies show that troponin testing in this setting has a sensitivity of 95%, specificity of 80% and a positive likelihood ratio of 4.75 and a negative likelihood ratio of 0.1.

Now what is the probability that this patient has an acute myocardial infarction?

*

Each answer must be between 0 and 100
Only an integer value may be entered in this field.

Please write your answer here:

© 2019 Brush JE Jr et al. JAMA Network Open.

Session 2 - Case 17 of 20

An 86-year-old woman is transferred to the emergency department from her nursing home. Over the last two days she has developed a new cough, fever and has become increasingly confused. At baseline she has mild cognitive impairment but is normally able to participate in a simple conversation. Now she is lethargic and her speech is illegible. The nursing home transfer record indicates that she has been coughing up dark green sputum.

Her past medical history is remarkable for hypertension, diet-controlled diabetes, coronary artery disease, a remote stroke (which left her with left-sided paralysis) and mild dementia. She is a non-smoker and does not drink alcohol.

On physical examination, her vital signs are temperature 39.5 C; pulse 118 / minute, blood pressure 120/96 / minute; respiratory rate 30 / minute. Her cardiac exam reveals a regular rate and rhythm with no murmurs, gallops or rubs. On auscultation of her chest, she has crackles in the left base. Her oropharynx and neck exam reveal no abnormalities. Her extremities have no swelling, and there are no visible rashes.

On a scale of 0 (very IMprobable) to 100 (very PROBABLE), what is the probability that this patient has pneumonia? *

Each answer must be between 0 and 100
Only an integer value may be entered in this field.

Please write your answer here:

© 2019 Brush JE Jr et al. JAMA Network Open.

Your initial probability answer was:
{INSERTANS:156367X3371X27315}

A chest x-ray is performed. The radiologist interprets the chest x-ray as showing evidence of consolidation.

Studies show that a chest x-ray showing consolidation is predictive for pneumonia with a sensitivity of 70% and specificity of 78% with a positive likelihood ratio of 3.2 and a negative likelihood ratio of 0.4.

What is the probability that this patient has pneumonia?

*

Each answer must be between 0 and 100
Only an integer value may be entered in this field.

Please write your answer here:

© 2019 Brush JE Jr et al. JAMA Network Open.

Session 2 - Case 18 of 20

A 57-year-old man presents to the emergency department for evaluation of 5 days of a cough, productive of green sputum. He has been feeling generally weak and tired over this same time period. He complains of a sore throat and coryza. He thinks he may have a fever and notes several episodes of rigors. He has had no nausea or vomiting, and reports no rashes or joint pains. He denies chest pain. He blames his teenage daughter for giving him the virus she just recovered from.

His past medical history is unremarkable. He quickly acknowledges that he doesn't believe in medications and doesn't take them. In fact, he has never had the influenza vaccine. He has no allergies. He is a social smoker and does not use any illicit drugs.

On examination, his temperature is 39.1C, pulse 106/minute; blood pressure 110/96mmHg; and respiratory rate 25/minute.

In general, he appears tired but non-toxic, and is speaking full sentences. He has dry mucous membranes, his oropharynx has no erythema or exudate, and he has no anterior or posterior chain cervical lymphadenopathy. On chest exam, he has crackles at the right base. His cardiac exam is normal. His skin demonstrates no rashes, petechiae, or purpura.

On a scale of 0 (very IMprobable) to 100 (very PROBABLE), what is the probability that this patient has pneumonia? *

Each answer must be between 0 and 100
Only an integer value may be entered in this field.

Please write your answer here:

© 2019 Brush JE Jr et al. JAMA Network Open.

Your initial probability answer was: {INSERTANS:156367X3372X27318}

A chest x-ray is performed. The radiologist interprets the chest x-ray as no evidence of consolidation.

Studies show that a chest x-ray showing consolidation is predictive for pneumonia with a sensitivity of 70% and specificity of 78% with a positive likelihood ratio of 3.2 and a negative likelihood ratio of 0.4.

What is the probability that this patient has pneumonia?

*

Each answer must be between 0 and 100
Only an integer value may be entered in this field.

Please write your answer here:

© 2019 Brush JE Jr et al. JAMA Network Open.

Session 2 - Case 19 of 20

A 30-year-old man presents to the emergency department complaining of worsening cough and shortness of breath for the last two days. His illness began 2 days prior to the cough when he started to experience malaise, and low-grade fever. When his breathing started to get worse, he increased his inhaled bronchodilator use and started an inhaled corticosteroid, thinking his asthma was flaring. He presents now because the medications are not working and he is coughing up yellow sputum.

His past medical history only includes well controlled asthma. His typical flare is caused by a viral upper respiratory infection. He is a regular smoker but has drastically reduced this week with a promise to quit because he feels so unwell.

On examination his temperature is 37.9C, pulse 116/minute; blood pressure 139/96mmHg; and respiratory rate 25/minute.

In general, he appears breathless. His cardiac exam is within normal limits. His respiratory exam reveals moderate wheezes bilaterally in all lung fields. There are no associated adventitious sounds, crackles or rales.

On a scale of 0 (very IMprobable) to 100 (very PROBABLE), what is the probability that this patient has pneumonia? *

Each answer must be between 0 and 100
Only an integer value may be entered in this field.

Please write your answer here:

© 2019 Brush JE Jr et al. JAMA Network Open.

Your initial probability answer was:
<u>{INSERTANS:156367X3373X27321}</u>

A chest x-ray is performed. The radiologist interprets the chest x-ray as showing evidence of consolidation.

Studies show that a chest x-ray showing consolidation is predictive for pneumonia with a sensitivity of 70% and specificity of 78% with a positive likelihood ratio of 3.2 and a negative likelihood ratio of 0.4.

What is the probability that this patient has pneumonia?

*

Each answer must be between 0 and 100
Only an integer value may be entered in this field.

Please write your answer here:

© 2019 Brush JE Jr et al. JAMA Network Open.

Session 2 - Case 20 of 20

A 23-year-old woman presents to the emergency department complaining of cough, sore throat and coryza for the past three days. She has not been feeling herself this week. Her boyfriend was recently seen by his family physician for a similar illness and treated with conservative measures. She notes that she can't get sick; work is busy this week. "This is the reason I got the flu shot."

Her past medical history is remarkable for mitral valve prolapse. Her only medication is an oral contraceptive pill. She smokes only in social settings but has been smoking more this week because of job stress. She is allergic to NSAIDs, and because of this she has avoided any over-the-counter medications for her symptoms.

On examination, her temperature is 37.1C; pulse 96 / minute; blood pressure 108/96mmHg; and respiratory rate 16 / minute. Her chest is resonant to percussion bilaterally. There are no crackles or adventitious sounds on bilateral auscultation. Her heart is regular with a fast rate with a 2/6 holosystolic murmur heard loudest at the apex without radiation. She has an inflamed oropharynx with bilateral tender, enlarged anterior cervical lymph nodes.

On a scale of 0 (very IMprobable) to 100 (very PROBABLE), what is the probability that this patient has pneumonia? *

Each answer must be between 0 and 100
Only an integer value may be entered in this field.

Please write your answer here:

© 2019 Brush JE Jr et al. JAMA Network Open.

Your initial probability answer was:
{INSERTANS:156367X3374X27324}

A chest x-ray is performed. The radiologist interprets the chest x-ray as no evidence of consolidation.

Studies show that a chest x-ray showing consolidation is predictive for pneumonia with a sensitivity of 70% and specificity of 78% with a positive likelihood ratio of 3.2 and a negative likelihood ratio of 0.4.

What is the probability that this patient has pneumonia?

*

Each answer must be between 0 and 100
Only an integer value may be entered in this field.

Please write your answer here:

© 2019 Brush JE Jr et al. JAMA Network Open.

Demographics

Birth Year (YYYY) *

Only numbers may be entered in this field.

Please write your answer here:

What year of medical school are you in? *

Only numbers may be entered in this field.

Please write your answer here:

Your current medical school: *

Please choose **only one** of the following:

- Eastern Virginia Medical School
- Michael G. DeGroote School of Medicine - McMaster University
- Other

© 2019 Brush JE Jr et al. JAMA Network Open.

Last Question. Please estimate the number of patients you have seen with the following diagnoses. *

Please choose the appropriate response for each item:

	less than 5	5 - 20	more than 20
Pneumonia	○	○	○
Congestive heart failure	○	○	○
Pulmonary Embolism	○	○	○
Acute coronary syndrome	○	○	○

© 2019 Brush JE Jr et al. JAMA Network Open.

Submit your survey.
Thank you for completing this survey.

© 2019 Brush JE Jr et al. JAMA Network Open.
