# Supplementary material for: Effect of Teaching Bayesian Methods Using Learning by Concept vs Learning by Example on Medical Students’ Ability to Estimate Probability of a Diagnosis: A Randomized Clinical Trial
Source: JAMA Netw Open. 2019 Dec 20;2(12):e1918023. doi: 10.1001/jamanetworkopen.2019.18023 (PMC7027434; doi:10.1001/jamanetworkopen.2019.18023)
Supplement: Supplement 3. — Data Sharing Statement [file jamanetwopen-2-e1918023-s003.pdf]

Data Sharing Statement

Brush, Jr. Effect of Teaching Bayesian Methods Using Learning by Concept vs Learning by Example on Medical Students' Ability to Estimate Probability of a Diagnosis. *JAMA Netw Open*. Published December 20, 2019. 10.1001/jamanetworkopen.2019.18023

Data
Data available: No

Additional Information
Explanation for why data not available: The data were not made available because, given the limited capacity of the group performing this educational clinical trial, the investigators did not have the capacity to maintain ongoing open availability of the source data.
